# Glycan-Silica Nanoparticles as Effective Inhibitors for Blocking Virus Infection

**DOI:** 10.1021/acsami.4c15918

**Published:** 2025-02-05

**Authors:** Carmen Pérez-Alonso, Fátima Lasala, Laura Rodríguez-Pérez, Rafael Delgado, Javier Rojo, Javier Ramos-Soriano

**Affiliations:** † Glycosystems Laboratory, Instituto de Investigaciones Químicas (IIQ), CSIC − Universidad de Sevilla, Av. Américo Vespucio 49, Seville 41092, Spain; ‡ Laboratorio de Microbiología Molecular Instituto de Investigación Hospital 12 de Octubre (imas12), 28041 Madrid, Spain; § Departamento de Química Orgánica, Facultad de Química, Universidad Complutense, 28040 Madrid, Spain

**Keywords:** silica nanoparticles, carbohydrates, bionanomaterials, DC-SIGN, L-SIGN, viral infection

## Abstract

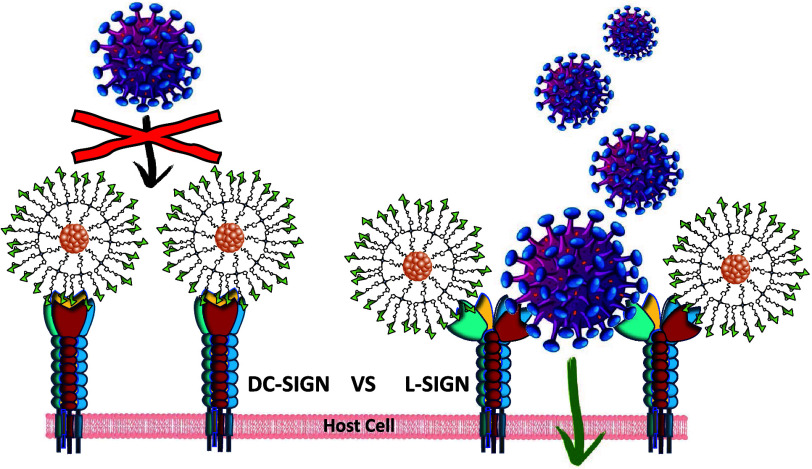

Small solid silica nanoparticles (SiNPs) have been used for multivalent carbohydrate presentation in DC-/L-SIGN-mediated viral infection models. Glycosylated SiNPs (glycoSiNPs) were fully characterized by different experimental techniques, including NMR, DLS, TGA, FTIR, and XPS, which confirmed their chemical structures. As a proof-of-concept, the capacity of glycoSiNPs to interact with Concanavalin A (ConA), a model lectin, using DLS binding experiments and UV−vis turbidimetry assays was analyzed. Their antiviral activity was assessed in a cellular assay using an artificial Ebola virus, demonstrating the potent inhibition of DC-SIGN-mediated infection. Notably, glycoSiNPs functionalized with a trivalent Manα1,2Man glycodendron exhibited the strongest inhibitory activity, with an IC_50_ of 135 ng/mL and a 170-fold lower efficiency in blocking L-SIGN-mediated viral infection. These findings suggest that glycoSiNPs present a promising approach for developing antiviral agents that selectively target the DC-SIGN pathway over the L-SIGN one.

## Introduction

Carbohydrates stand as ubiquitous biomolecules, playing crucial roles in many physiological and pathological processes, including inflammation, cell differentiation, fertilization, infection, tumor progression and metastasis, etc.^1^ Their functionality often relies on interactions with specific receptors such as lectins. Despite the high selectivity of these carbohydrate–lectin interactions, their inherent weakness prompts nature to employ a multivalent strategy. By involving multiple copies of both the carbohydrate ligand and the receptor, simultaneous interactions occur, resulting in enhanced avidity and selectivity of the recognition process.^2^ Numerous examples in the literature illustrate a diverse range of supports employed as platforms for the artificial multivalent presentation of sugars.^3^ These multifaceted systems have served as invaluable tools, providing pertinent insights into the binding processes and finding applications in both biological and biomedical contexts.

A C-type lectin receptor (CLR) that has recently attracted attention from the scientific community due to its crucial role in infection and immunomodulation processes is the Dendritic Cell-Specific ICAM-3 Grabbing Nonintegrin (DC-SIGN).^4^ This lectin mainly recognizes fucosylated and mannosylated oligosaccharides in a calcium-dependent manner and is expressed on the surface of dendritic cells (DCs) as homotetramers.^5^ DC-SIGN engages in high-affinity interactions with glycosylated envelope glycoproteins (gp120 of HIV, GP1 of Ebola virus, etc.), which present numerous copies of high-mannose glycans. Pathogens, including these viruses, exploit DC-SIGN as the gateway for cellular entry and infection.^6^ Indeed, this multivalent interaction serves as the fundamental event, facilitating the internalization of the virus into cells, marking the initial step in the infection process. Inhibiting this recognition process holds the key to halting the progression of these pathogens in the early stages of infection. To achieve this aim, it is necessary to develop carbohydrate multivalent systems as highly efficient inhibitors capable of competing for this receptor and stop the viral entrance.^7^

Among the diverse scaffolds used for a multivalent presentation of sugars, examples including metal nanoparticles, polymers, dendrimers, peptides and proteins, fullerene and other carbon nanoforms, calixarenes, cyclodextrins, and nanogels, among others,^3,8^ have been extensively employed for the preparation of glycoconjugates as antivirals. Silica nanoparticles (SiNPs) constitute a relatively unexplored class of biocompatible and unconventional scaffolds for developing multivalent glycoconjugates with potential biological applications, with only a few examples reported in the literature.^9−15^ SiNPs provide a distinct advantage due to their ease of preparation and precise size control.^16^ Furthermore, their low toxicity and cost render them as an ideal platform for the multivalent presentation of sugars. SiNPs exist in two primary forms: mesoporous and solid. Mesoporous SiNPs (MSNs) possess cavities capable of effectively storing molecules, making them widely used for transporting cargo. These nanoparticles can be decorated with ligands on the surface, enabling them especially suitable for applications such as targeted delivery systems.^17−21^ On the other hand, solid SiNPs, smaller than MSNs, also have the capability of being functionalized with ligands at their surface. Despite these capabilities, there are limited examples of the functionalization of solid SiNPs with carbohydrates at the surface.^22−25^ Notably, this approach has largely been unexplored in the field of biomedical applications.

For all of the above considerations, we designed and prepared highly efficient multivalent inhibitors for DC-SIGN-mediated viral infections by synthesizing mannosylated glycoderivatives built around a biocompatible small solid SiNPs scaffold. Based on previous studies that highlighted the use of α-mannose-α-1,2-mannose (Manα1,2Man or diMan) enhanced the affinity for DC-SIGN by 3−4 times in comparison with the monosaccharide α-mannose (Man),^26,27^ these glycoSiNPs were decorated with both Man and Manα1,2Man or trivalent dendrons of these carbohydrates to compare their capacity to block DC-SIGN and therefore to inhibit pathogen infection. In these studies, we have found that glycoSiNPs functionalized with Manα1,2Man trivalent glycodendrons are potent inhibitors of Ebola viral infection (IC_50_ = 135 ng/mL). None of the glycoSiNPs exhibited cytotoxic effects within the concentration range investigated in the infection experiments.

## Results and Discussion

**Scheme 1:**
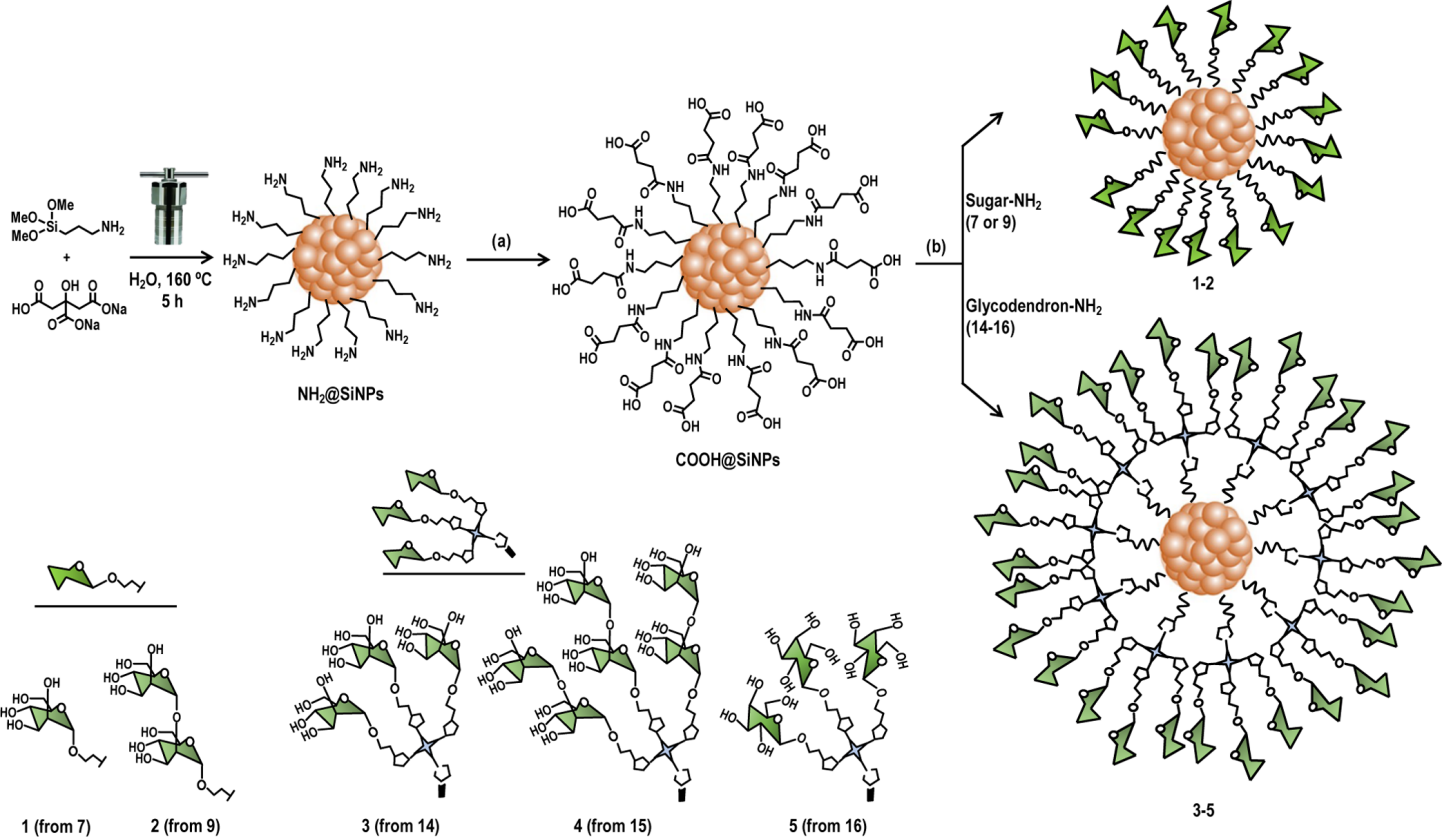
General Procedure for the Synthesis of GlycoSiNPs Decorated with Mannose **1** (Man@SiNPs), Disaccharide Manα1,2Man **2** (diMan@SiNPs) and Mannose **3** (Man_3_@SiNPs), Manα1,2Man **4** (diMan_3_@SiNPs), and Galactose **5** (Gal_3_@SiNPs) Trivalent Glycodendrons *Reagents and Conditions*: (a) Succinic Anhydride, H_2_O, MW 60 °C, 1 h; (b) **7**–**9** or **14**–**16**, HATU, DIPEA, DMF, r.t., Overnight.

**Figure 1:**
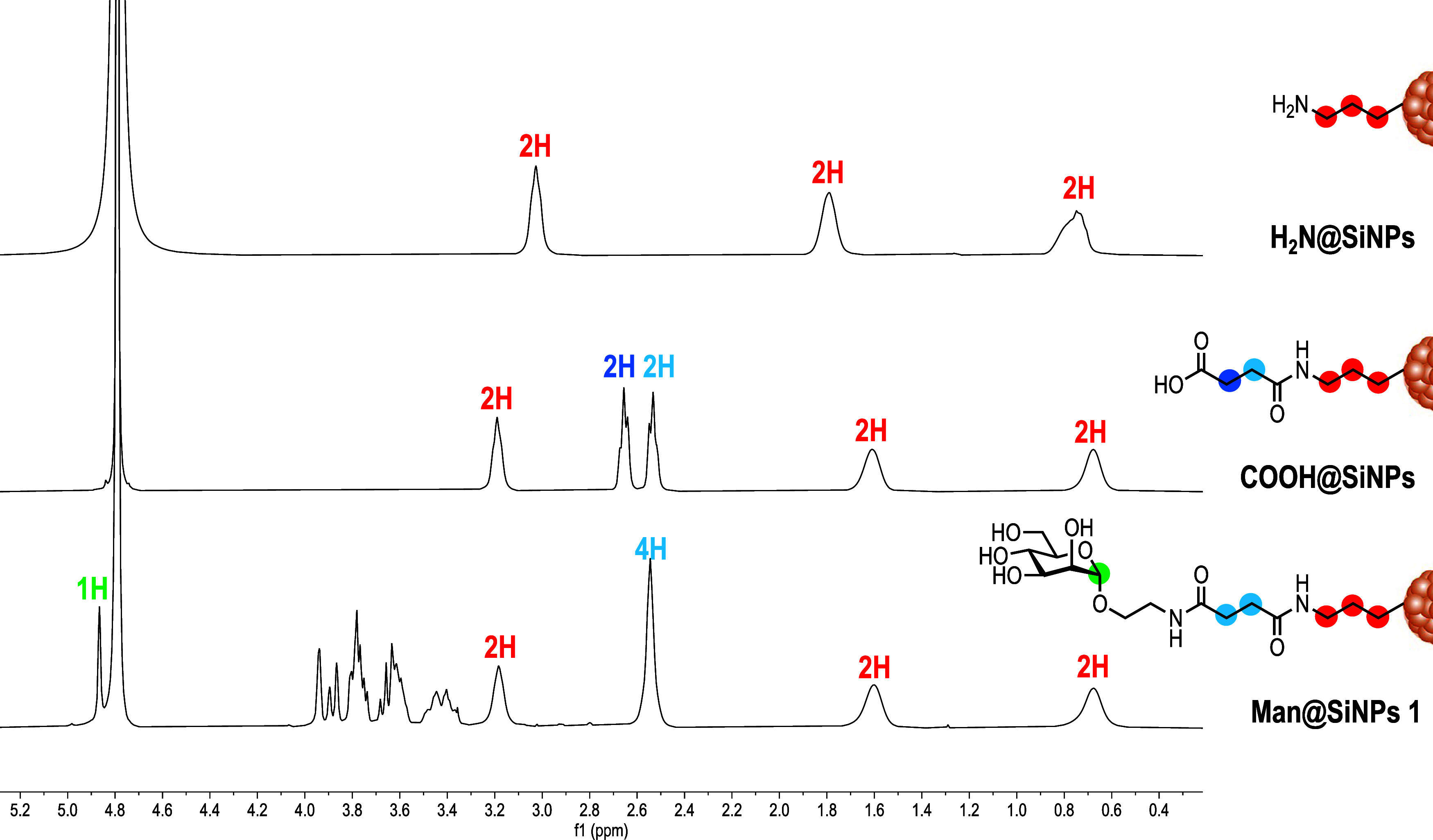
^1^H NMR spectra of NH_2_@SiNPs, COOH@SiNPs, and Man@SiNPs **1**, from top to bottom, respectively. Protons in red correspond to APTMS moieties, in blue to CH_2_ of succinyl groups, and in green to the anomeric proton of mannose residues.

The synthesis of silica-based glyconanoparticles (glycoSiNPs) **1**-**5** was carried out as depicted in Scheme 1. Initially, water-dispersible amine-terminated silicon-based nanoparticles (NH_2_@SiNPs) were synthesized on a scale of grams from (3-aminopropyl)trimethoxysilane (APTMS) and trisodium citrate under hydrothermal conditions, following a previously reported method by De Cola *et al.*(28,29) As reported, residual citric acid was observed on the surface of the pristine NH_2_@SiNPs using ^1^H NMR (Figure S1). To remove this residual citric acid and prevent potential issues in subsequent reactions, the SiNPs were treated with a 1 M NaOH solution and then purified by size-exclusion chromatography using a Sephadex G25. The absence of signals corresponding to the residual citric acid was confirmed by ^1^H NMR spectrum (Figure 1). Next, amine-terminated SiNPs were reacted with succinic anhydride under basic conditions and microwave irradiation at 60 °C for 1 h to yield carboxylic-acid-modified SiNPs (COOH@SiNPs). After purification by Sephadex G25, the complete functionalization of the particles was confirmed by integrating the signals in the ^1^H NMR spectrum (Figure 1). This spectrum exhibited two signals for the succinyl moiety (2H each) and three signals corresponding to the CH_2_ groups of the APTMS residue (2H each).

**Scheme 2:**
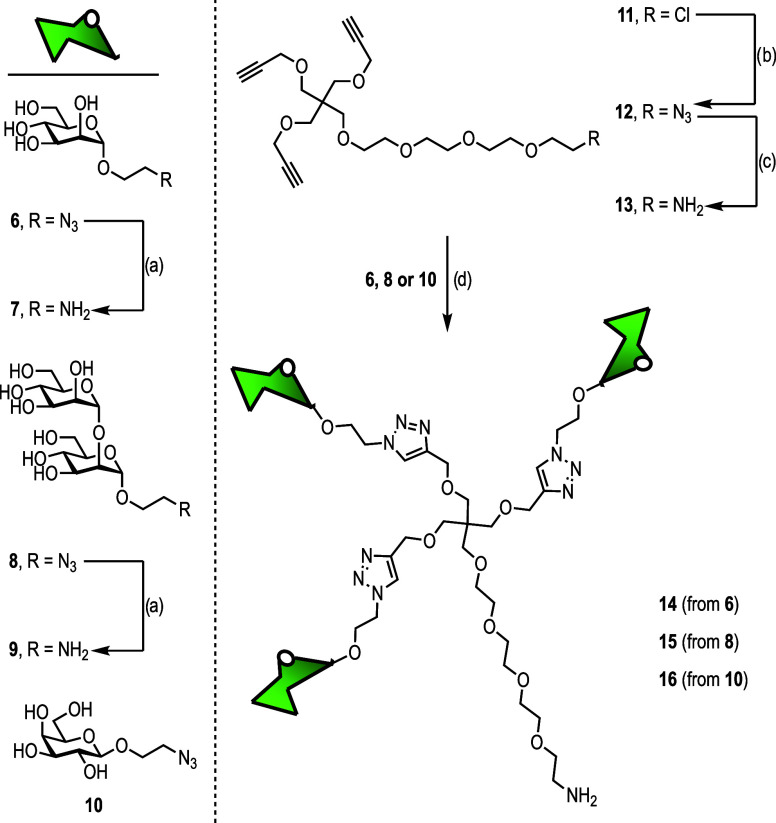
General Procedure for the Synthesis of 1-Aminoethyl Sugars **7** and **9** and Amine Trivalent Glycodendrons Decorated with Mannose **14**, Disaccharide Manα1,2Man **15**, and Galactose **16** *Reagents and Conditions*: (a) H_2_, Pd/C, MeOH/HCl, r.t, 30 min; (b) NaN_3_, KI, DMF, 60 °C, Overnight; (c) PPh_3_, H_2_O, THF, r.t., Overnight; (d) CuSO_4_·5H_2_O, TBTA, NaAsc, MW 60 °C, 30 min.

With COOH@SiNPs in hand, we synthesized 1-aminoethyl mannose **7**(30) and Manα1,2Man disaccharide **9** by hydrogenation at atmospheric pressure of 1-azidoethyl mannoside **6**(30) and mannobioside **8**,^31^ respectively (Scheme 2). Both azido compounds **6** and **8** were also conjugated to [2-[2-(2-aminoethoxy)ethoxy]ethoxy]ethoxymethyl trikis(2-propyniloxy-methyl)methane (**13**) by a CuAAC reaction to generate the corresponding amine-bearing trivalent glycodendrons **14**–**15**, respectively, in moderate to very good yields after Sephadex LH20 purification (see ESI for full experimental details). For comparison purposes, the preparation of trivalent glycodendrons decorated with galactose **16** was also carried out. The CuAAC reactions were addressed using CuSO_4_, sodium ascorbate, and tris[(1-benzyl-1*H*-1,2,3-triazol-4-yl)methyl]amine (TBTA). Prior to purification, all solutions were processed with Quadrasil MP resin to eliminate any residual copper that might affect the biological tests. These glycodendrons were satisfactorily characterized by both NMR (Figures S4−S15) and MS.

To obtain multivalent glycoSiNPs **1**–**5**, the surface of acid-coated SiNPs was successfully conjugated to amine derivatives **7**–**9** or **14**−**16** via HATU-mediated coupling (Scheme 1). Following the amide bond formation, purification of these glyconanomaterials was achieved by Sephadex G25 to remove unreacted carbohydrates and byproducts such as tetramethylurea and 1-hydroxy-7-azabenzotriazole (HOAt) from the HATU coupling reagent. The resulting pure glyconanoparticles were characterized using spectroscopic, thermal, and microscopic techniques.

Evidence of the complete functionalization of COOH@SiNPs with the corresponding carbohydrates was initially obtained by NMR, providing a quantitative estimation of 100% covalent functionalization on all glycoSiNPs. Despite being nanoparticles of low polydispersity, both ^1^H and ^13^C NMR spectra of glycoSiNPs **1**–**5** are remarkably simple (Figures S16−S25). In the ^1^H NMR spectra, the signals of the CH_2_ groups of succinyl moieties are observed to be equivalent (δ ≈ 2.5 ppm) due to the amidation reaction, in contrast to those of COOH@SiNPs where these signals appear as two multiplets (δ ≈ 2.7 and 2.5 ppm). In essence, after functionalization with carbohydrates, the signal of CH_2_ bound to carboxylic groups revealed an upfield shift from 2.7 to 2.5 ppm. Moreover, the integration of this signal and its comparison with the signals from carbohydrate or glycodendron moieties revealed a perfect correlation, confirming complete functionalization. For instance, analysis of integrals of signals corresponding to CH_2_ of the succinyl groups and the anomeric proton for glycoSiNPs **1**–**2** (Figures 1 and S18), and the anomeric proton or triazole ring signals for glycoSiNPs **3**–**5** (Figures S20, S22 and S24), revealed ratios of 4:1 and 4:3, respectively.

**Figure 2:**
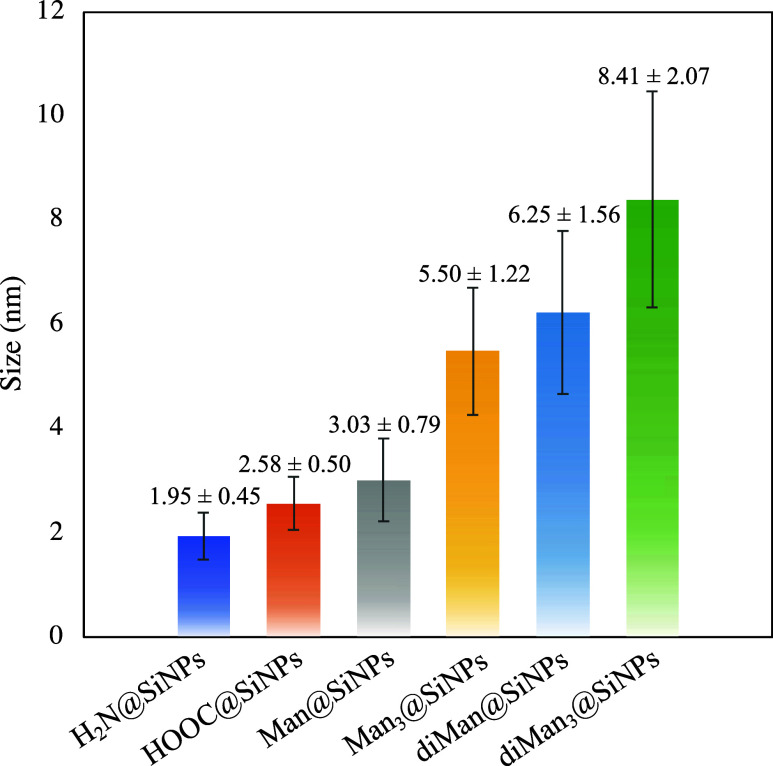
Hydrodinamic diameters of NH_2_@SiNPs, COOH@SiNPs, Man@SiNPs **1**, diMan@SiNPs **2**, Man_3_@SiNPs **3**, diMan_3_@SiNPs **4**, and Gal_3_@SiNPs **5**, respectively, determined by the DLS technique.

The surface charge of all SiNPs was characterized using ζ potential (Table S1), while their hydrodynamic diameter (Figures 2 and S26) was evaluated through dynamic light scattering (DLS). The hydrodynamic diameter measurements provided valuable insights into the success of each reaction step.

In fact, we observed an increase in the hydrodynamic diameters as the SiNPs were functionalized, with values of 1.95 ± 0.45 nm for NH_2_@SiNPS, 2.58 ± 0.50 nm for COOH@SiNPs, 3.03 ± 0.79 nm for Man@SiNPs **1** decorated with mannose, and 5.50 ± 1.22 nm for the corresponding Man_3_@SiNPs **3** functionalized with the trivalent mannose glycodendron (Figure 2). Similarly, an expected increase can be observed from diMan@SiNPs **2** (6.25 ± 1.56 nm) decorated with Manα1,2Man disaccharide to those coated with the corresponding trivalent glycodendron diMan_3_@SiNPs **4** (8.41 ± 2.07 nm).

**Figure 3:**
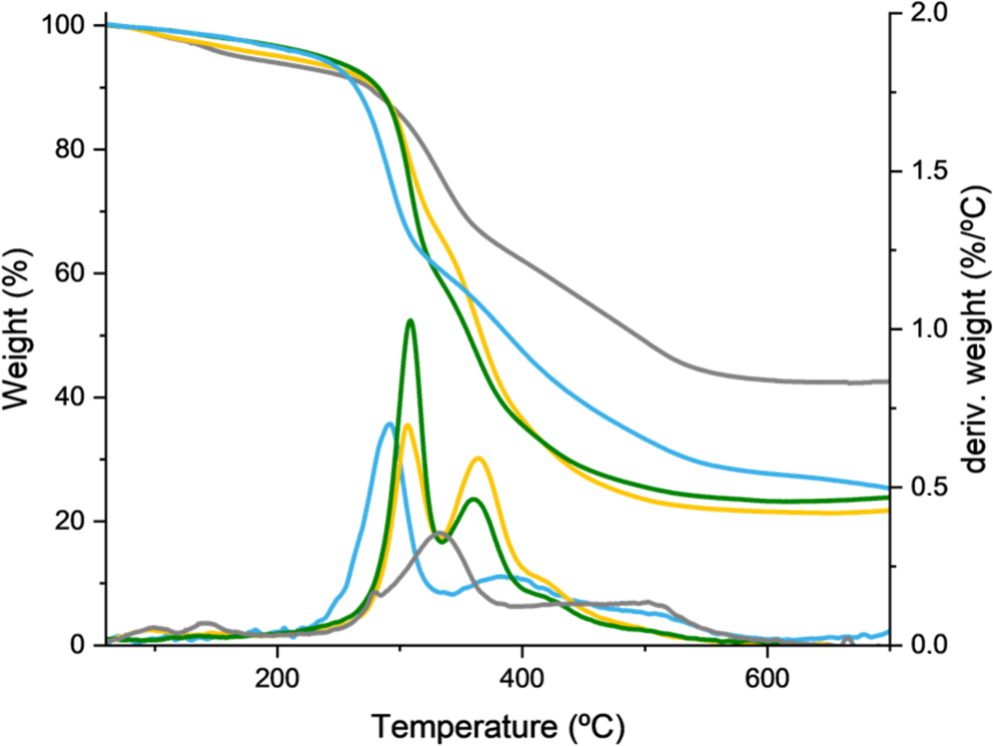
TGA analysis of Man@SiNPs **1** (gray), diMan@SiNPs **2** (blue), Man_3_@SiNPs **3** (yellow), and diMan_3_@SiNPs **4** (green).

To further corroborate the complete functionalization of glycoSiNPs **1**–**5**, we recorded the TGA thermograms. As expected, a significantly higher degree of weight loss was obtained for the glycodendron-coated SiNPs **3**–**4** compared to glycoSiNPs **1**–**2** (Figure 3). This finding could be rationalized according to the molecule size and the number of carbohydrates anchored to the nanoparticle scaffold. More specifically, the thermal decomposition of the mannose groups in glycoSiNPs **4**, bearing the trivalent glycodendron **15**, was 75%, compared to 67% in **2**, a nanoparticle functionalized with disaccharide. The same trend was observed for **1** and **3** where the increase of weight loss goes from 52% for **1** to 77% for **3**. All samples present two weight loss contributions due to the organic functionalization and the scaffold, which remains intact, attributed to the SiNPs itself. Moreover, no weight loss around 200 °C of carboxylic acid groups of COOH@SiNPs (Figure S27) is detected for any of the samples, confirming the total covalent functionalization with the glycoderivatives. The reproducibility of the present covalent grafting was studied by comparing the mannose **3** and galactose **5** glycodendron-coated SiNPs. As seen in Figure S28, trivalent glycoSiNPs **3** and **5** behave in a similar manner with an average of 75% loss of weight.

**Figure 4:**
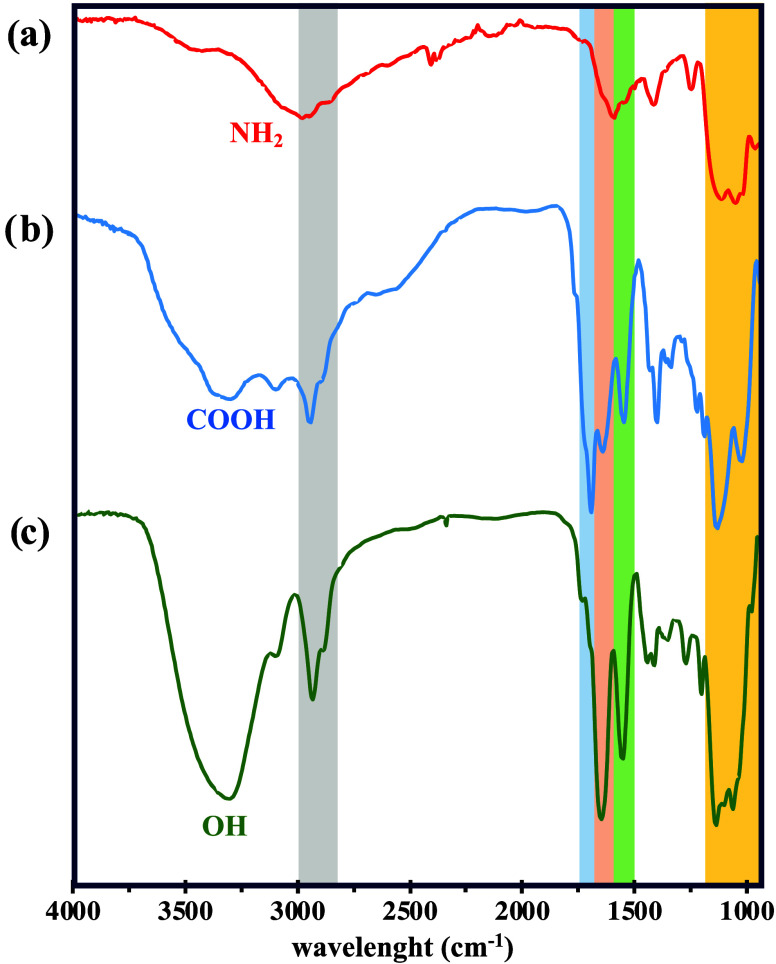
FTIR characterization of (a) pristine NH_2_@SiNPs (red), (b) COOH@SiNPs (blue), and (c) Man@SiNPs **1** (green) decorated with mannose residues.

Fourier-transform infrared spectroscopy (FTIR) has been performed for identifying functional groups present on the surface of the intermediates and final glycoconjugates (Figures 4 and S29). Notably, typical peaks for silicon oxide vibration modes occurring between 1150 and 1000 cm^−1^ were observed for all SiNPs. Additional evidence supporting the amide linkage comes from the observation of two sharp bands at 1700 and 1640 cm^−1^, attributed to the C=O acid vibration and C=O stretching of the amide carbonyl, supporting the conjugation of succinic anhydride. On the contrary, after conjugation of carbohydrates and glycodendrons on the COOH@SiNPs, only one sharp band at 1640 cm^−1^ was observed. Moreover, the peak at 1550 cm^−1^ is attributed to C−N stretching and N−H bending from the amide group, which is absent in the precursor NH_2_@SiNPs. FTIR spectra of glycoSiNPs **1**–**5** show a broad peak centered around 3300 cm^−1^, attributed to vibrations of the −OH groups of the carbohydrate moieties (Figure 4). This is in contrast to the two bands at ≈3280 and 3075 cm^−1^ for −OH of the carboxylic acid groups (COOH@SiNPs) and a broad peak at 3012 cm^−1^ for N−H stretching from the amine groups (NH_2_@SiNPs). Additional features observed near 2930 cm^−1^ are assigned to the C−H stretching vibrations of the alkane chains.

All of these observed features, identified using different techniques, confirm that the NH_2_@SiNPs are indeed functionalized with acid groups (COOH@SiNPs) and subsequently capped with sugar molecules to produce the desired glycoSiNPs.

**Figure 5:**
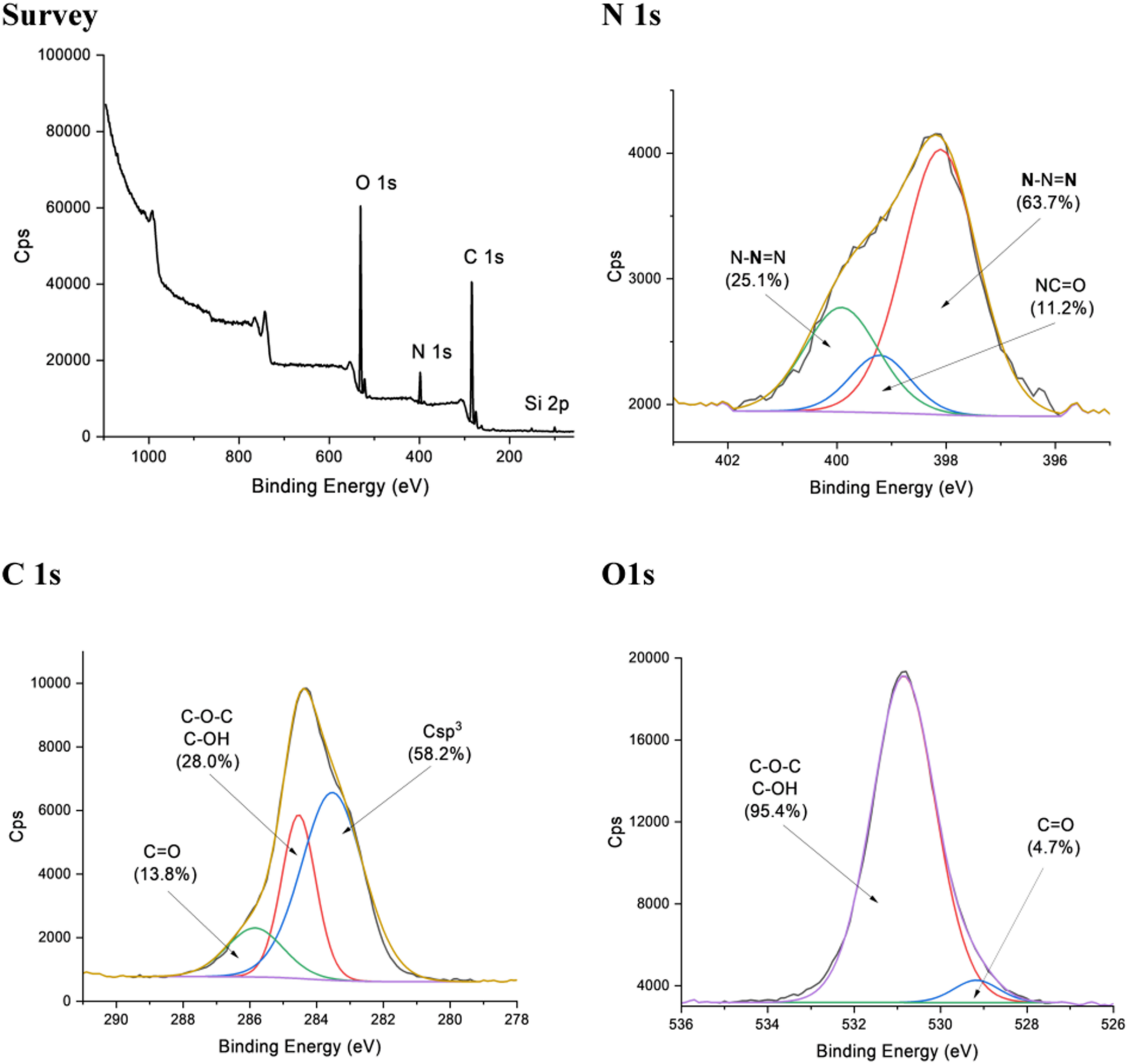
XPS analysis of **3**: survey spectra and the detailed deconvolution of the high resolution of N 1s, C 1s, and O 1s.

Additional insights into the elemental composition of glycoSiNPs were obtained by X-ray photoelectron spectroscopy (XPS). The survey spectra of glycoSiNPs **1**–**5** display the spectroscopic signatures of C, O, N, and Si (Figures 5 and S30). However, a detailed semiquantitative analysis of the four elements mentioned analyzed with XPS reveals differences in their relative abundances (Table 1). As expected, the Si atomic percentage of the core of the glycoSiNPs decreases drastically when increasing the complexity (mono- or disaccharide) and number (monovalent or trivalent glycodendron) of carbohydrates anchored on the SiNPs shell, reducing its percentage from 6.7% in **1** to 1% in **4**. But more interesting is the analysis of the high-resolution spectra of the different elements. The deconvolution of the C 1s energy-level signals was performed using three Gaussian−Lorentzian curves, which corresponded to the different carbon atoms (C sp^2^/C sp^3^, C=O, and C−O/C−OH) present in all glycoSiNPs (Figures 5 and S31). The deconvolution of the N 1s energy-level signal (Figure S32) for glycoSiNPs **3**–**5** leads to three peaks. Analyzing the organic structure of this glycoSiNP, three oxidized states of N are found corresponding to three deconvolutions. The first peak located at higher binding energy is assigned to an N atom of the triazole ring, while the other two N atoms generate the second peak, which is positioned 1 eV lower.^32^ The minor contribution with the lowest binding energy corresponds to the N of the amide groups. As expected, no peak of the free azide groups at 405.0 eV was found. In contrast, glycoSiNPs **1**–**2** were fitted with only one contribution at 398.6 eV because only the amide group is presented on their structures. The nature of the oxygen-containing groups was also analyzed with the O 1s high-resolution spectra. Figures 5 and S33 show the deconvolution of the O 1s peak for all glycoSiNPs with the following bands: carbonyl oxygen atoms in amides at ∼530 eV with minor contribution and O atoms in OH or ethers at ∼531 eV with the largest deconvolution band.^33^

**Table 1:** XPS Relative Abundance and Position (eV, in Brackets) for GlycoSiNPs **1–5**

glycoSiNP	% C 1s	% N 1s	% O 1s	% Si 2p
**1**	60.14 (284.6)	6.70 (398.6)	26.43 (531.6)	6.72 (101.6)
**2**	56.25 (284.6)	5.86 (398.6)	33.63 (531.6)	4.26 (101.6)
**3**	64.67 (284.6)	7.90 (398.6)	26.75 (530.6)	1.17 (100.6)
**4**	65.80 (284.6)	6.30 (398.6)	26.90 (530.6)	0.99 (100.6)
**5**	65.13 (284.6)	7.92 (399.6)	25.73 (531.6)	1.23 (99.6)

Preliminary binding studies were conducted using Concanavalin A (ConA) as a tetravalent (at physiological pH) model protein to analyze the ability of glycoSiNPs **1**–**4** to be recognized by lectins and determine the multivalent effect. Galactosylated Gal_3_@SiNPs **5** were used as a negative control since this lectin recognizes mannoses. To establish a correct comparison between different nanoparticles, we first estimated the amount of sugar in the glycoSiNPs using the sulfuric acid-UV method^34^ after performing a calibration curve (Figure S34). According to this method, glycoSiNPs **1**–**2** prepared from simple mono- and disaccharides, respectively, contain between 20 and 22% (w/w) of sugar. A similar result was obtained for the glycoSiNPs **3**–**5** (25, 29, and 25%, respectively) prepared from the much bulkier dendritic blocks. These values are summarized in Table 2.

**Table 2:** Carbohydrate Content^a^ Determined for 1 mg of GlycoSiNPs **1–5**

glycoSiNP	%carbohydrate^a^	carbohydrate (μg/mg)^a^
**1**	20	205.6
**2**	22	210.5
**3**	25	265.8
**4**	29	305.1
**5**	25	276.5

a Content of mannose in **1**, Manα1,2Man disaccharide in **2**, mannose glycodendron in **3**, Manα1,2Man disaccharide in **4**, and galactose glycodendron in **5**.

**Figure 6:**
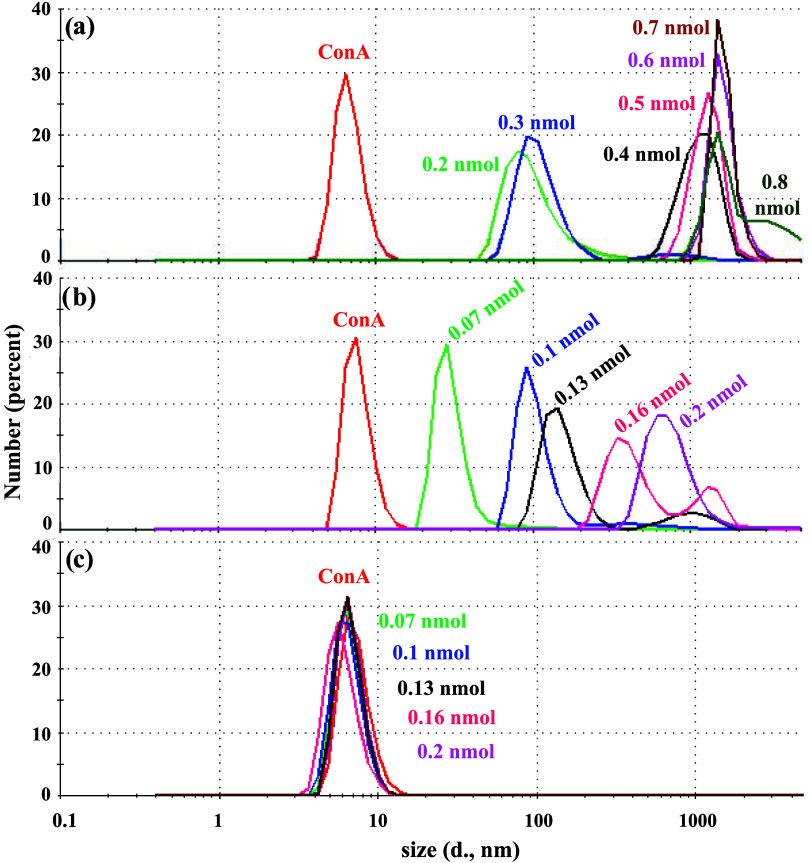
DLS number size histograms of titration of ConA with (a) Man@SiNPs **1**, (b) Man_3_@SiNPs **3**, and (c) Gal_3_@SiNPs **5** at different concentrations. ([ConA lectin] = 0.45 mg/mL, buffer: 0.1 M Tris-HCl pH 7.2, 0.9 M KCl, 1 mM MnCl_2_, and 1 mM CaCl_2_).

Binding studies were conducted by DLS and UV spectroscopy. The size of homotetramer ConA at pH 7.2 was measured by DLS (6.80 ± 1.42 nm; Figure S35) and subsequently monitored its agglutination, resulting from cross-linking of the tetrameric ConA by glycoSiNPs, achieved through the addition of increasing amounts of different glycoSiNPs (Figures 6 and S36−S39, Table S2).

As depicted in Figure 6, DLS binding experiments showed larger particles as soon as very small amounts of glycoSiNPs were added to ConA. These results revealed an increased binding capacity of the glycoSiNPs with enhanced multivalency glycoSiNPs **3** (Man_3_@SiNPs) and **4** (diMan_3_@SiNPs) in comparison with the corresponding glycoSiNPs **1** (Man@SiNPs) and **2** (diMan@SiNPs), respectively, decorated with the simple mono- and disaccharide. For instance, Man_3_@SiNPs **3** rapidly aggregated with ConA, forming large species (>1000 nm) from 436 ng (0.2 nmol of mannose) (Figure 6b). In contrast, for the lower valency glycoSiNPs, a 1.5-fold higher concentration of Man@SiNPs **1** (630 ng, 0.7 nmol of mannose) was necessary to observe such a significant effect (Figure 6a). A similar multivalent effect was observed for diMan@SiNPs **2** and the analogous decorated with the corresponding trivalent glycodendron, diMan_3_@SiNPs **4** (Figures S37 and S39). These findings showed that increasing the valency has a significant impact on the capacity of clustering, as previously described for other multivalent glycosystems by our research group.^35−39^ As expected, no aggregation was detected for the control Gal_3_@SiNPs **5** (Figure 6c), proving the specific lectin-binding properties of the mannosylated glycoSiNPs (i.e., the carbohydrate on the SiNPs retains its affinity and selectivity). Owing to the reversible nature of the interaction, the aggregates were entirely dissolved upon the addition of an α-d-mannose saturated solution (Figures S36−S39).

**Figure 7:**
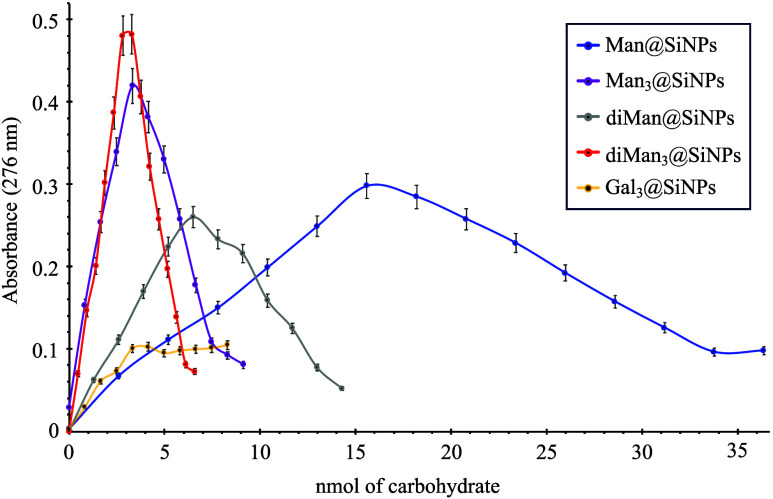
UV−vis turbidimetry assays as a function of per-sugar basis (nmol) ([ConA lectin] = 0.45 mg/mL, buffer: 0.1 M Tris-HCl pH 7.2, 0.9 M KCl, 1 mM MnCl_2_, and 1 mM CaCl_2_).

A similar trend was observed in UV−vis turbidimetry assays (Figure 7, Tables S3 and S4), conducted on per-sugar basis (i.e., accounting for the carbohydrate nmol of different glycosystems). In this case, an increase in absorbance at 276 nm was noted upon the formation of aggregates between the lectin and the glycoSiNPs or even precipitation associated with a significant reduction of the absorbance peak, which was visible within seconds after the addition of glycoSiNPs **1**–**4** to ConA (Figure S40). The suspended aggregates gradually precipitated out of the solution. It is important to note that the presence of the Manα1,2Man disaccharide (diMan@SiNPs **2**) significantly increased the binding activity (higher increase in the absorbance) when compared with the monosaccharide (Man@SiNPs **1**). Moreover, the addition of Man_3_@SiNPs **3** and diMan_3_@SiNPs **4** to a ConA solution induced the formation of large aggregates at lower concentrations compared to those of glycoSiNPs **1** and **2**. Again, minimal changes in the absorbance could be observed for galactosylated Gal_3_@SiNPs **5**, showing no signs of aggregation. The reversibility of the interaction was once more confirmed by the addition of an α-d-mannose saturated solution, which resulted in the complete elimination of the turbidity (Figure S40).

Interestingly, glycoSiNPs **1**–**5** exhibited exceptional stability in solution or the solid state for at least 1.5 years and could be freeze-dried for storage, thus expanding their applicability as potential therapeutics.

Once these silica glyconanoparticles were prepared and characterized, as well as validated to interact with ConA, they were deployed as inhibitors in a viral infection model. The activity of the glycoSiNPs **1**–**4** as inhibitors of cell *cis*-infection by Ebola virus (EBOV) was addressed.

### Biological Studies

DC-SIGN and L-SIGN are C-type lectin receptors specialized in recognizing carbohydrate molecular patterns associated with pathogens, mainly binding to mannosylated oligosaccharides.^40^ Both related homotetrameric lectins share a very high degree of sequence homology, with 82% overall similarity in the full-length protein^41,42^ and up to 72% similarity within the carbohydrate recognition domain (CRD). Despite this sequence conservation, the four CRDs are not identical, displaying distinct topologies (adopting different conformations) and dynamics. Such structural variations have a significant impact on certain aspects of the recognition event of multivalent glycoconjugates.^43,44^ While both receptors share structural homology, their biological functions diverge significantly. DC-SIGN, predominantly expressed on immature DCs, modulates the immune response by recognizing pathogen-associated carbohydrate patterns. In contrast, L-SIGN, found in human liver sinusoidal endothelial cells and human lung in type II alveolar cells, is not directly involved in immunity. However, both DC-SIGN and L-SIGN are important receptors that are crucial in facilitating viral infections, including HIV, Zika, and Ebola viruses, by mediating viral entry into host cells.^3^ In this context, the development of potent glycoconjugated viral inhibitors, such as our glycoSiNPs, offers clear advantages over other antiviral strategies.^45^ By effectively blocking the entry of virals into host cells, these inhibitors can significantly reduce the likelihood of viral mutations and the development of resistance. Moreover, their therapeutic efficacy is less likely to be compromised by such mutations, providing a potentially long-lasting solution to viral infections.^46^

The efficiency of glycoSiNPs **1**–**4** to inhibit DC- and L-SIGN-mediated infection of EBOV was performed in a *cis*-infection assay using pseudotyped viral particles endowed with EBOV glycoprotein GP1 (EBOV-GP). As positive control of the infection process, infection with Vesicular Stomatitis Virus envelope glycoprotein (VSV-G) pseudotyped lentiviruses, which is independent of the presence of both DC- or L-SIGN receptors, was assessed. As a negative control, Gal_3_@SiNPs **5** with galactose instead of mannose were included in the experiments as this carbohydrate is not recognized by either DC-SIGN or L-SIGN.

**Figure 8:**
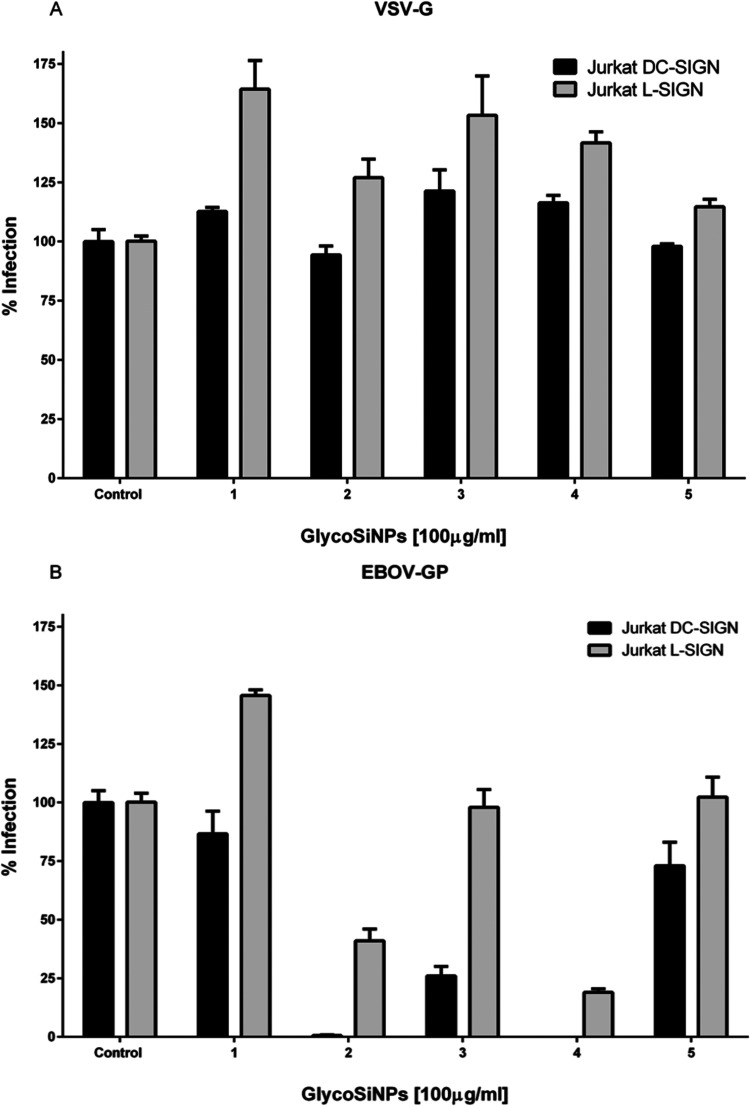
Effect of glycoSiNPs **1**–**5** on infection of (a) VSV-G (control) and on (b) EBOV-GP pseudoviruses on Jurkat-DC-SIGN (black bars) or Jurkat-L-SIGN (gray bars) cells.

For this purpose, Jurkat cells expressing DC-SIGN or L-SIGN lectin were incubated for 30 min with each glycoSiNPs **1**–**4** for a final concentration of 100 μg/mL and then infected with VSV-G or EBOV-GP pseudotyped lentiviruses. None of the glycoSiNPs **1**–**4** was capable of inhibiting infection of the VSV-G pseudotypes (Figure 8a). Infection levels (RLU) are very similar to or without each nanoparticle, which indicates that none of the glycoSiNPs **1**–**4** at this concentration (100 ug/mL) exhibited a significant cytotoxic effect, enabling the investigation of their potential biological function role in preventing viral infections.

In contrast, diMan@SiNPs **2** and diMan_3_@SiNPs **4** inhibited very efficiently the direct entry of EBOV-GP pseudotypes, both in cells expressing DC- or L-SIGN lectin at 100 μg/mL (Figure 8b). Man_3_@SiNPs **3** only shows inhibitory activity against EBOV infection in DC-SIGN cells, whereas Man@SiNPs **1** shows no inhibitory activity against EBOV infection in both DC- or L-SIGN cells. These results also revealed the carbohydrate-dependent inhibition effect, as previously observed by our group with other multivalent glycoconjugates.^3,7,8^ As expected, Gal_3_@SiNPs **5** have no effect on virus entry, for either VSV or EBOV, further demonstrating the specific lectin-binding properties of the mannosylated glycoSiNPs, as previously observed with ConA.

Once the capacity of these glycoSiNPs to inhibit the infection process was demonstrated, we studied the inhibition process at different concentrations to obtain the IC_50_ values (Table 3 and Figure S41). So, cells were cultured in media containing increasing concentrations of glycoSiNPs **2**–**4** (from 0 to 100 μg/mL) for 30 min and then infected with EBOV pseudotyped viral particles. After 48 h, virus infection was assessed and presented as a percentage of inhibition with respect to the control.

**Table 3:** IC_50_ (ng/mL) Values for GlycoSiNPs **2-3**

glycoSiNP	J-DC-SIGN	J-L-SIGN
**2**	919 (648−1304)	69,227 (24,283−197,356)
**3**	55,946 (12,189−256,795)	∼130,870 (very wide)
**4**	135 (93−197)	22,960 (19,894−26,497)

The three glycoSiNPs **2**–**4** showed higher efficiency on Jurkat DC-SIGN as compared with Jurkat L-SIGN (Table 3). GlycoSiNPs **4** (diMan_3_@SiNPs), with mannobioses in a dendrimeric presentation, show the best IC_50_ value (135 ng/mL) on Jurkat-DC-SIGN and exhibit a 170-fold higher inhibitory ability over Jurkat-L-SIGN (22,960 ng/mL). The analogous with a monovalent presentation, diMan@SiNPs **2**, has an intermediate IC_50_ value (919 ng/mL for Jurkat-DC-SIGN and 69,227 ng/mL for Jurkat-L-SIGN), and finally, Man_3_@SiNPs **3** with a dendrimeric presentation of monosaccharide mannose show the highest IC_50_ values on both Jurkat-DC-SIGN and L-SIGN. As previously observed with ConA, these results revealed an increased inhibitory capacity for the glycoSiNPs with enhanced multivalency (diMan_3_@SiNPs **4**) in comparison with the corresponding diMan@SiNPs **2**. A significantly greater inhibitory activity was also observed when comparing SiNPs functionalized with the disaccharide diMan (glycoSiNPs **4**) to those decorated with the corresponding monosaccharide Man (glycoSiNPs **3**).

**Figure 9:**
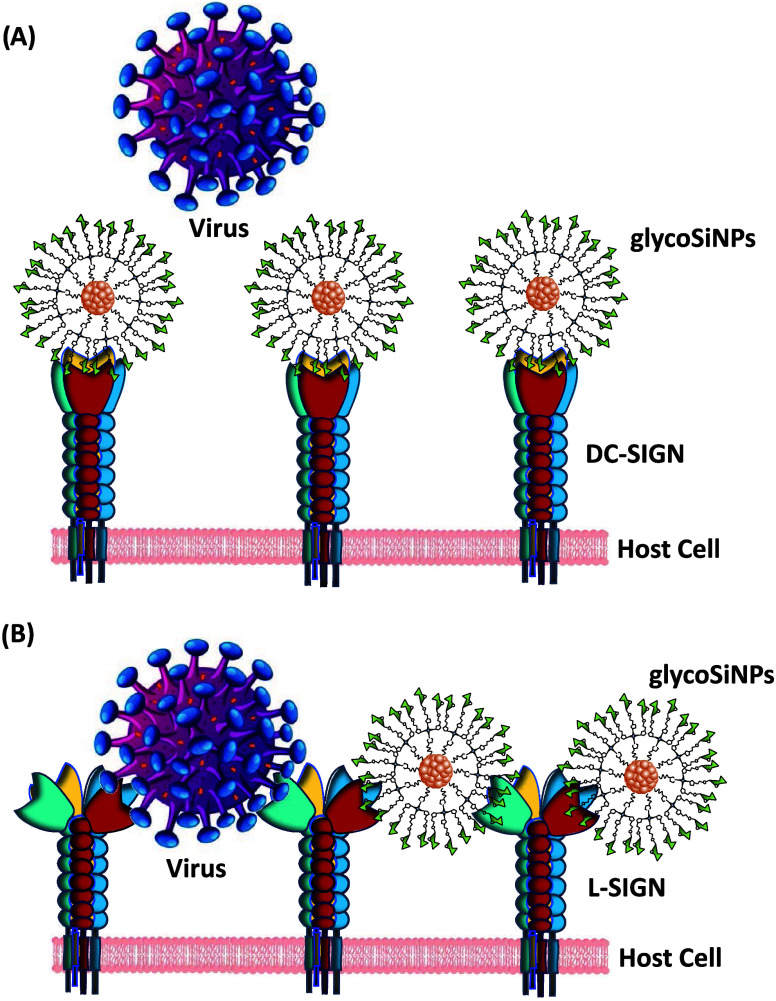
Representation of the proposed interaction between glycoSiNPs and (a) the four CRDs of DC-SIGN, inhibiting DC-SIGN-dependent viral infection and (b) cross-linking with two of the four CRDs of L-SIGN, allowing the virus to interact with the unblocked binding sites of L-SIGN in L-SIGN-dependent viral infection model.

The observed differences in IC_50_ values could likely be attributed to the distinct spacing and structural arrangement of the two tetrameric lectins. As mentioned previously, their CRDs, and consequently their binding sites, are oriented differently in space: DC-SIGN’s binding sites are positioned facing upward, while those of L-SIGN are oriented more laterally, increasing the distance between adjacent and opposing sites (L-SIGN: 60 and 80 Å vs DC-SIGN: 40 and 60 Å, respectively). Taking these considerations into account, we propose that each glycoSiNP could bind simultaneously to all four binding sites of DC-SIGN, effectively inhibiting its capacity to interact with viral surface EBOV-GPs and thereby preventing viral entry (Figure 9a). In the case of L-SIGN, each glycoSiNP could interact with only two of the four binding sites, possibly through a cross-linking mode of interaction. The remaining unoccupied sites could serve as footholds for the virus to bind to EBOV-GPs, facilitating cellular uptake and infection (Figure 9b). A similar mechanism has been previously proposed in studies using Au- or QD-conjugated glycans, as reported by Zhou and colleagues.^44,47^

## Conclusions

We have carried out the synthesis of less-explored multivalent glycoconjugates based on small SiNPs as scaffolds, in which monovalent and/or trivalent carbohydrates of different natures (Man, diMan, or Gal) have been covalently connected. Characterization of these glycoSiNPs has been performed using NMR, DLS, TGA, FTIR, and XPS. As a proof-of-concept, the interaction of glycoSiNPs with Concanavalin A (ConA), a model lectin, was analyzed using DLS binding experiments and UV−vis turbidimetry assays.

As DC/L-SIGN lectins are two of the receptors involved in EBOV entry into the cells, using the new multivalent silica-based mannosylated glycoconjugates to block these receptors presents a novel strategy to inhibit the viral infection process. Their effectiveness in blocking DC/L-SIGN-mediated viral infections by EBOV has been tested in a cellular experimental assay. Mannosylated glycoSiNPs showed specific lectin-binding properties. They only inhibited direct entry of the Ebola virus through DC/L-SIGN lectins but not the virus control (VSV) whose entry into the cell is independent of these lectins. The three glycoSiNPs **2**–**4** showed higher efficiency on Jurkat DC-SIGN as compared with Jurkat L-SIGN. This can be attributed to a topologically more favorable interaction between these glycomaterials with the CRDs on DC-SIGN lectin than with L-SIGN. It is important to highlight that the presence of diMan significantly enhances the biological activity compared to Man. GlycoSiNPs **4**, with a higher number of mannobioside units ((diMan)_3_), shows the best IC_50_ value (135 ng/mL) for Ebola virus infection on Jurkat-DC-SIGN. Moreover, diMan_3_@SiNPs **4** showed a 170-fold higher efficiency in blocking DC-SIGN versus L-SIGN-mediated viral infection. Interestingly, all glycoSiNPs show no cytotoxicity up to a concentration of 100 μg/mL and were capable of inhibiting the infection process in a receptor-, mannose-, and multivalent-dependent way.

Owing to their low-/nontoxicity, excellent biocompatibility, and ease of preparation with precise size control, the glycoSiNPs are perfectly suited for a broad spectrum of applications, including the creation of novel therapeutic strategies targeting deadly viral infections based on the blockade of carbohydrate receptors and immune disorders (allergy, cancer, autoimmune diseases, etc.).

## Materials and Methods

### Chemistry

2-Azidoethyl-α-d-mannopyranoside (**6**) and 2-aminoethyl-α-d-mannopyranoside (**7**),^30^ 2-azidoethyl-β-d-galactopyranoside (**10**),^48^ 2-azidoethyl α-d-mannopyranosyl-(1 → 2)-α-d-mannopyranoside (**8**),^31^ [2-[2-(2-chloroethoxy)ethoxy]ethoxy]ethoxymethyl trikis(2-propyniloxy-methyl)methane (**11**),^35^ and **NH**_**2**_**@SiNPs**(28,29) were prepared according to previously reported procedures in the literature.

### COOH@SiNPs

To a solution of **NH**_**2**_**@SiNPs** (50 mg) in H_2_O (1.5 mL) in a sealed microwave vial, Et_3_N (100 μL) and succinic anhydride (50 mg) were added. The reaction mixture was heated in a microwave oven (60 °C, 1 h), quenched with 1 M HCl (0.15 mL), and purified by Sephadex G25 (H_2_O/MeOH 9:1), yielding **COOH@SiNPs** (152 mg) as a white amorphous solid. ^1^H NMR (400 MHz, D_2_O) *δ*: 3.19 (m, 2H, Hc), 2.66 (m, 2H, He), 2.53 (m, 2H, Hd), 1.61 (m, 2H, Hb), 0.68 (m, 2H, Ha); ^13^C NMR (100 MHz, D_2_O) *δ*: 176.7 (CO_acid_), 174.0 (CO_amide_), 41.8 (Cc), 30.5 (Ce), 29.7 (Cd), 22.3 (Cb), 9.2 (Ca).

#### 2-Aminoethyl α-d-mannopyranosyl-(1 → 2)-α-d-mannopyranoside **(9)**

A solution of 2-azidoethyl α-d-mannopyranosyl-(1 → 2)-α-d-mannopyranoside (**8**) (50 mg, 0.12 mmol) in 1 M HCl/MeOH (4 mL, 1:9) was hydrogenated (P = 1 atm, r.t., 30 min) using Pd/C (10%, 5 mg). Then, the reaction mixture was filtered through Celite and washed with MeOH. After solvent removal, compound **9** (56 mg, 99%) was isolated as a yellowish oil. ^1^H NMR (400 MHz, D_2_O) *δ*: 5.15 (s, 1H, H-1_B_), 5.05 (s, 1H, H-1_A_), 4.09−3.62 (m, 14H, H-2_A_, H-2_B_, H-3_A_, H-3_B_, H-4_A_, H-4_B_, H-5_A_, H-5_B_, H-6_A_, H-6_B_, OC*H*_2_CH_2_NH_2_), 3.28 (m, 2H, OCH_2_C*H*_2_NH_2_); ^13^C NMR (100 MHz, D_2_O) *δ*: 102.2 (C-1_B_), 98.3 (C-1_A_), 78.5 (C-2_A_), 73.2 (C-5_A_), 72.9 (C-5_B_), 70.2 (C-3_B_), 69.9 (C-2_B_), 66.9 (C-4_A_), 66.9 (C-4_B_), 63.5 (O*C*H_2_CH_2_NH_2_), 61.2 (C-6_B_), 60.9 (C-6_A_), 39.1 (OCH_2_*C*H_2_NH_2_); ESI-MS: *m*/*z* calcd. for C_14_H_27_NO_11_: 385.2; found: 386.2 [M + H]^+^; ESI-HRMS *m*/*z* calcd. for C_14_H_28_NO_11_ [M + H]^+^: 386.1657; found: 386.1650.

#### [2-[2-(2-Azidoethoxy)ethoxy]ethoxy]ethoxymethyl trikis(2-propyniloxy-methyl)methane **(12)**

To a solution of [2-[2-(2-chloroethoxy)ethoxy]ethoxy]ethoxymethyl trikis(2-propyniloxy-methyl)methane (**11**) (200 mg, 0.45 mmol) in DMF (2 mL), sodium azide (145.5 mg, 2.24 mmol) and KI (14.9 mg, 0.09 mmol) were added. The reaction mixture was stirred (r.t., 60 °C, overnight), and the solvent was evaporated. The crude was diluted (EtOAc), washed (H_2_O), dried (MgSO_4_), filtered, and concentrated. Through purification by silica gel column chromatography (EtOAc/*n*-hexane, 1:2), compound **12** (155 mg, 76%) was furnished as a colorless oil. ^1^H NMR (400 MHz, CDCl_3_) *δ:* 4.10 (d, 6H, ^4^*J*_H,CCH_ = 2.4, OC*H*_*2*_CCH), 3.70−3.57 (m, 14H, CH_2_O, OC*H*_*2*_CH_2_N_3_), 3.51 (s, 6H, CCH_2_O), 3.46 (s, 2H, CC*H*_*2*_OCH_2_CH_2_), 3.39 (t, 2H, ^3^*J*_H,H_ = 5.0, OCH_2_C*H*_*2*_N_3_), 2.41 (m, 3H, ^4^*J*_H,CCH_ = 2.4, CCH); ^13^C NMR (100 MHz, CDCl_3_) *δ:* 80.2 (*C*CH), 74.2 (C*C*H), 71.2, 70.9, 70.8, 70.6, 70.3, 70.0 (O*C*H_2_CH_2_N_3_), 69.9 (C*C*H_2_OCH_2_CH_2_), 69.0 (C*C*H_2_O), 58.6 (O*C*H_2_CCH), 50.6 (OCH_2_*C*H_2_N_3_), 44.9 (*C*CH_2_O); ESI-MS: *m*/*z* calcd. for C_22_H_33_N_3_O_7_: 451.2; found: 474.4 [M + Na]^+^; FAB-HRMS *m*/*z* calcd. for C_22_H_33_N_3_O_7_Na [M + Na]^+^: 474.2211; found: 474.2210.

#### [2-[2-(2-Aminoethoxy)ethoxy]ethoxy]ethoxymethyl trikis(2-propyniloxy-methyl)methane **(13)**

To a solution of azide **12** (160 mg, 0.35 mmol) and PPh_3_ (232 mg, 0.89 mmol) in THF (2 mL), H_2_O (7 μL) was added. The mixture was stirred (r.t., overnight), and the solvent was removed. Through purification by silica gel column chromatography (CH_2_Cl_2_/MeOH/Et_3_N 100:0:0.1% → 20:1:1%), the amine derivative **13** (136 mg, 91%) was furnished as a yellowish oil. ^1^H NMR (400 MHz, CDCl_3_) *δ*: 4.12 (s, 6H, OC*H*_2_CCH), 3.71−3.50 (m, 20H, CH_2_O, OC*H*_2_CH_2_NH_2_, CCH_2_O), 3.45 (s, 2H, CC*H*_*2*_OCH_2_CH_2_), 2.87 (t, 2H, ^3^*J*_H,H_ = 5.0, OCH_2_C*H*_2_NH_2_), 2.4 (s, 3H, CCH); ^13^C NMR (100 MHz, CDCl_3_) *δ*: 80.2 (*C*CH), 74.2 (C*C*H), 73.1 (O*C*H_2_CH_2_NH_2_), 71.3, 70,4, 69.9 (C*C*H_2_OCH_2_CH_2_), 69.2 (C*C*H_2_O), 58.8 (O*C*H_2_CCH), 45.1 (*C*CH_2_O), 41.8 (OCH_2_*C*H_2_NH_2_); ESI-MS: *m*/*z* calcd. for C_22_H_35_NO_7_: 425.2; found: 426.3 [M + H]^+^, 448.3 [M + Na]^+^; FAB-HRMS: *m*/*z* calcd. for C_22_H_36_NO_7_ [M + H]^+^: 426.2486; found: 426.2471.

#### Glycodendron **14**

Following the general procedure (see ESI) and using 2-azidoethyl-α-d-mannopyranoside (**6**) (210 mg, 0.85 mmol) and compound **13** (100 mg, 0.24 mmol), glycodendron **14** (86 mg, 53%) was obtained as a yellowish solid. ^1^H-RMN (400 MHz, CD_3_OD) *δ*: 8.01 (s, 3H, H_triazole_), 4.75 (s, 3H, H-1), 4.65 (m, 6H, OCH_2_C*H*_*2*_N_triazole_), 4.57 (s, 6H, OCH_2_C_triazole_), 4.14 (m, 3H, OC*H*HCH_2_N_triazole_), 3.90 (m, 3H, OCH*H*CH_2_N_triazole_), 3.83−3.75 (m, 6H, H-2, H-6a), 3.74−3.43 (m, 31H, H-4, H-5, H-6b, CCH_2_O, CC*H*_*2*_OCH_2_CH_2_, CH_2_O, OC*H*_*2*_CH_2_NH_2_), 3.27 (m, 3H, H-3), 3.16 (m, 2H, OCH_2_C*H*_*2*_NH_2_); ^13^C NMR (100 MHz, CD_3_OD) *δ*: 146.2 (C_triazole_), 125.8 (CH_triazole_), 101.7 (C-1), 74.9 (C-3), 72.5 (C-5), 72.1, 71.9 (C-2), 71.5, 71.4, 71.3, 71.1, 70.7, 70.1, 68,3 (C-4), 67.9 (O*C*H_2_CH_2_NH_2_), 66.7 (O*C*H_2_CH_2_N_triazole_), 65.3 (O*C*H_2_C_triazole_), 62.8 (C-6), 51.3 (OCH_2_*C*H_2_N_triazole_), 46.6 (*C*CH_2_O), 40.7 (OCH_2_*C*H_2_NH_2_); ESI-MS: *m*/*z* calcd. for C_46_H_80_N_10_O_25_: 1172.9; found: 1171.9 [M-H]^−^.

#### Glycodendron **15**

Following the general procedure (see ESI) and using 2-azidoethyl α-d-mannopyranosyl-(1 → 2)-α-d-mannopyranoside (**8**) (100 mg, 0.24 mmol) and compound **13** (29 mg, 67 μmol), glycodendron **15** (90 mg, 81%) was obtained as a yellowish solid. ^1^H NMR (400 MHz, D_2_O) *δ*: 8.06 (s, 3H, H_triazole_), 5.06 (s, 3H, H-1_A_), 4.97 (s, 3H, H-1_B_), 4.66 (s, 6H, OCH_2_C*H*_*2*_N_triazole_), 4.58 (s, 6H, OCH_2_C_triazole_), 4.13−4.02 (m, 6H, H-2_B_, OC*H*HCH_2_N_triazole_), 3.93 (3H, m, OCH*H*CH_2_N_triazole_), 3.90−3.53 (m, 44H, H-6_A_, H-6_B_, H-5_A_, H-4_A_, H-4_B_, H-3_A_, H-3_B_, H-2_A_, OCH_2_, OC*H*_*2*_CH_2_NH_2_), 3.46 (s, 6H, CCH_2_O), 3.41 (s, 2H, CC*H*_*2*_OCH_2_CH_2_), 3.22 (m, 2H, OCH_2_C*H*_*2*_NH_2_), 3.05 (m, 3H, H-5_B_); ^13^C NMR (100 MHz, D_2_O) *δ*: 145.8 (C_triazole_), 126.9 (CH_triazole_), 103.8 (C-1_B_), 99.5 (C-1_A_), 80.1 (C-2_A_), 74.7 (C-5_A_), 74.3 (C-5_B_), 72.0, 71.8 (C-3_B_), 71.5 (C-2_B_), 71.4 (C-3_A_), 71.1, 71.0, 70.6 (C*C*H_2_OCH_2_CH_2_), 69.8 (C*C*H_2_O) 68.3 (C-4_A_), 68.0 (C-4_B_), 67.8 (O*C*H_2_CH_2_NH_2_), 67.1 (O*C*H_2_CH_2_N_triazole_), 65.0 (O*C*H_2_C_triazole_), 62.6 (C-6_B_), 62.1 (C-6_A_), 51.5 (OCH_2_*C*H_2_N_triazole_), 46.2 (*C*CH_2_O), 40.6 (OCH_2_*C*H_2_NH_2_); ESI-MS: *m*/*z* calcd. for C_64_H_110_N_10_O_40_: 1658.7; found: 1657.9 [M-H]^−^.

#### Glycodendron **16**

Following the general procedure (see ESI) and using 2-azidoethyl-β-d-galactopyranoside (**10**) (300 mg, 1.20 mmol) and compound **13** (142 mg, 0.33 mmol), glycodendron **16** (121 mg, 73%) was obtained as a yellowish solid. ^1^H-RMN (400 MHz, CD_3_OD) *δ*: 8.12 (s, 3H, H_triazole_), 4.66 (s, 6H, OCH_2_C*H*_*2*_N_triazole_), 4.54 (s, 6H, OCH_2_C_triazole_), 4.33−4.21 (m, 6H, H-1, OC*H*HCH_2_N_triazole_), 4.02 (m, 3H, OCH*H*CH_2_N_triazole_), 3.84 (m, 3H, H-3), 3.79−3.43 (m, 37H, H-4, H-5, H-2, H-6, CCH_2_O, CC*H*_*2*_OCH_2_CH_2_, OCH_2_, OC*H*_*2*_CH_2_NH_2_), 3.14 (m, 2H, OCH_2_C*H*_*2*_NH_2_); ^13^C NMR (100 MHz, CD_3_OD) *δ*: 145.8(C_triazole_), 126.3 (CH_triazole_), 105.1 (C-1), 76.7 (C-5), 74.8 (C-2), 72.3 (C-4), 72.1, 71.3, 71.1, 70.7, 70.2 (C-3), 70.0, 69.1 (O*C*H_2_CH_2_N_triazole_), 67.8 (O*C*H_2_CH_2_NH_2_), 65.2 (O*C*H_2_C_triazole_), 62.5 (C-6), 51.7 (OCH_2_*C*H_2_N_triazole_), 46.5 (*C*CH_2_O), 40.7 (OCH_2_*C*H_2_NH_2_); ESI-MS: *m*/*z* calcd. for C_46_H_80_N_10_O_25_: 1172.9; found: 1171.9 [M-H]^−^.

#### Man@SiNPs **1**

To a solution of 2-aminoethyl mannose **7** (260 mg, 1 mmol) and DIPEA (533 μL, 3 mmol) in dry DMF (3.4 mL), a solution of **COOH@SiNPs** (60 mg) and HATU (590 mg, 1.5 mmol) in dry DMF (3.4 mL) was dropwise added. The mixture was stirred (r.t., overnight). After solvent removal, the resulting crude was purified by Sephadex G25 (H_2_O/MeOH 9:1), yielding Man@SiNPs **1** (120 mg) as a dark yellow amorphous solid. ^1^H NMR (400 MHz, D_2_O) *δ*: 4.87 (s, 1H, H-1), 3.94 (m, 1H, H-2), 3.88 (m, 1H, H-6a) 3.81−3.74 (m, 3H, H-6b, OC*H*HCH_2_NHCO, H-3) 3.68−3.60 (m, 3H, OCH*H*CH_2_NHCO, H-5, H-4), 3.43 (m, 2H, OCH_2_C*H*_*2*_NHCO), 3.18 (m, 2H, Hc), 2.54 (m, 4H, Hd, He), 1.60 (m, 2H, Hb), 0.67 (m, 2H, Ha); ^13^C NMR (100 MHz, D_2_O) *δ*: 174.6 (CO), 99.7 (C-1), 72.8 (C-5), 70.5 (C-3), 70.0 (C-2), 66.7 (C-4), 65.8 (O*C*H_2_CH_2_NHCO), 60.9 (C-6), 41.7 (Cc), 38.9 (OCH_2_*C*H_2_NHCO), 31.2 (Cd, Ce), 22.3(Cb), 9.8 (Ca).

#### diMan@SiNPs **2**

To a solution of 2-aminoethyl Manα1,2Man **9** (56 mg, 0.13 mmol) and DIPEA (71 μL, 0.4 mmol) in dry DMF (0.5 mL), a solution of **COOH@SiNPs** (8 mg) and HATU (78 mg, 0.20 mmol) in dry DMF (0.5 mL) was dropwise added. The mixture was stirred (r.t., overnight). After solvent removal, the resulting crude product was purified by Sephadex G25 (H_2_O/MeOH 9:1), yielding diMan@SiNPs **2** (20 mg) as a dark yellow amorphous solid. ^1^H NMR (400 MHz, D_2_O) *δ*: 5.11 (s, 1H, H-1_A_), 5.02 (s, 1H, H-1_B_), 4.7 (s, 1H, H-2_A_), 3.96−3.59 (m, 13H, H-2_B_, H-3_B_, H-3_A_, H-4_B,_ H-4_A_, H-5_B_, H-5_A_, H-6_B_ H-6_A_, OC*H*_*2*_CH_2_NHCO), 3.41 (m, 2H, OCH_2_C*H*_*2*_NHCO), 3.17 (m, 2H, Hc), 2.59 (m, 4H, Hd, He), 1.59 (m, 2H, Hb), 0.67 (m, 2H, Ha); ^13^C NMR (100 MHz, D_2_O) *δ*: 174.2 (CO), 102.2 (C-1_B_), 98.3 (C-1_A_), 78.51 (C-2_B_), 73.2 (C-5_A_), 72.9 (C-5_B_), 70.3 (C-3_A_), 70.1 (C-3_B_), 69.9 (C-2_A_), 66.9 (C-4_A_, C-4_B_), 65.9 (O*C*H_2_CH_2_NHCO), 61.9 (C-6_B_), 60.9 (C-6_A_), 41.9 (Cc), 39.0 (OCH_2_*C*H_2_NHCO), 31.2 (Cd, Ce), 22.4 (Cb), 10.2 (Ca).

#### Man_3_@SiNPs **3**

To a solution of glycodendron **14** (129 mg, 0.1 mmol) and DIPEA (57 μL, 0.32 mmol) in dry DMF (0.5 mL), a solution of **COOH@SiNPs** (8 mg) and HATU (63 mg, 0.16 mmol) in dry DMF (0.5 mL) was dropwise added. The mixture was stirred (r.t., overnight). After solvent removal, the resulting crude product was purified by Sephadex G25 (H_2_O/MeOH 9:1), yielding Man_3_@SiNPs **3** (44 mg) as a dark yellow amorphous solid. ^1^H NMR (400 MHz, D_2_O) *δ*: 8.05 (s, 3H, H_triazole_), 4.66 (m, 6H, OCH_2_C*H*_*2*_N_triazole_), 4.56 (s, 6H, OCH_2_C_triazole_), 4.10 (m, 3H, OC*H*HCH_2_N_triazole_), 3.92 (m, 3H, OCH*H*CH_2_N_triazole_), 3.86 (m, 3H, H-2), 3.78−3.53 (m, 28H, H-4, H-5, H-6, CC*H*_*2*_OCH_2_CH_2_, CH_2_O, O*CH*_*2*_CH_2_NHCO), 3.47−3.35 (m, 8H, CCH_2_O, OCH_2_C*H*_*2*_NHCO), 3.16 (m, 2H, Hc), 3.09 (3H, m, H-3), 2.52 (m, 4H, Hd, He), 1.59 (m, 2H, Hb), 0.66 (m, 2H, Ha); ^13^C NMR (100 MHz, D_2_O) *δ*: 174.4 (CO), 144.3 (C_triazole_), 125.3 (CH_triazole_), 99.5 (C-1), 72.8 (C-3), 70.5 (C-5), 69.9, 69.6 (C-2), 69.5, 69.4, 69.2, 68.8, 68.3 (C-4), 65.4 (O*C*H_2_CH_2_N_triazole_), 63.5 (O*C*H_2_C_triazole_), 60.7 (C-6), 50.0 (OCH_2_*C*H_2_N_triazole_), 44.8 (*C*CH_2_O), 41.8 (Cc), 38.9 (OCH_2_*C*H_2_NHCO), 31.2 (Cd, Ce), 22.5 (Cb), 9.7 (Ca).

#### diMan_3_SiNPs **4**

To a solution of glycodendron **15** (85 mg, 50 μmol) and DIPEA (27 μL, 150 μmol) in dry DMF (375 μL), a solution of **COOH@SiNPs** (4 mg) and HATU (29 mg, 75 μmol) in dry DMF (375 μL) was dropwise added. The mixture was stirred (r.t., overnight). After solvent removal, the resulting crude product was purified by Sephadex G25 (H_2_O/MeOH 9:1), yielding diMan_3_@SiNPs **4** (30 mg) as a dark yellow amorphous solid. ^1^H NMR (400 MHz, D_2_O) *δ*: 8.06 (s, 3H, H_triazole_), 5.07 (s, 3H, H-1_A_), 4.98 (s, 3H, H-1_B_), 4.71−4.53 (m, 12H, OCH_2_C*H*_*2*_N_triazole_, OCH_2_C_triazole_), 4.14−4.03 (m, 6H, H-2_B_, OC*H*HCH_2_N_triazole_), 3.98−3.54 (m, 47H, H-2_A_, OCH*H*CH_2_N_triazole_, H-5_A_, H-4_A_, H-4_B,_ H-3_A,_ H-3_B_ H-6_A_, H-6_B_, OCH_2_, OCH_2_C*H*_*2*_NHCO), 3.52−3.16 (m, 10H, CCH_2_O, OCH_2_C*H*_*2*_NHCO, CC*H*_*2*_OCH_2_CH_2_), 3.17 (m, 2H, Hc), 3.05 (m, 3H, H-5_A_), 2.52 (m, 4H, Hd, He), 1.59 (m, 2H, Hb), 0.65 (m, 2H, Ha); ^13^C NMR (100 MHz, D_2_O) *δ*: 174.5 (CO), 144.3 (C_triazole_), 125.4 (CH_triazole_), 102.3 (C-1_B_), 99.5 (C-1_A_), 98.0, 78.6 (C-2_A_), 73.2 (C-5_A_), 72.8 (C-5_B_), 70.5, 70.4, 70.3 (C-3_B_), 70.1 (C-2_B_), 69.9 (C-3_A_), 69.6, 69.5 (C*C*H_2_OCH_2_CH_2_), 69.4 (C*C*H_2_O), 69.1, 68.9, 68.3 (C-4_B_, C-4_A_), 66.9, 66.5 (O*C*H_2_CH_2_NH_2_), 66.4, 65.6 (O*C*H_2_CH_2_N_triazole_), 65.4 (O*C*H_2_C_triazole_), 63.6, 61.1 (C-6_B_), 60.6 (C-6_A_), 50.0 (OCH_2_*C*H_2_N_triazole_), 44.8 (*C*CH_2_O), 41.8 (Cc), 38.9 (OCH_2_*C*H_2_NHCO), 31.2 (Cd, Ce), 22.1 (Cb), 8.9 (Ca).

#### Gal_3_@SiNPs **5**

To a solution of glycodendron **16** (129 mg, 0.1 mmol) and DIPEA (57 μL, 0.32 mmol) in dry DMF (0.5 mL), a solution of **COOH@SiNPs** (8 mg) and HATU (63 mg, 0.16 mmol) in dry DMF (0.5 mL) was dropwise added. The mixture was stirred (r.t., overnight). After solvent removal, the resulting crude product was purified by Sephadex G25 (H_2_O/MeOH 9:1), yielding Gal_3_@SiNPs **5** (34 mg) as a dark yellow amorphous solid. ^1^H NMR (400 MHz, D_2_O) *δ*: 8.07 (s, 3H, H_*t*riazole_), 4.65 (m, 6H, OCH_2_C*H*_*2*_N_triazole_), 4.54 (s, 6H, OCH_2_C_triazole_), 4.36 (dd, 3H, ^3^*J*_H1,H2_ = 7.7 Hz, H-1), 4.27 (m, 3H, OC*H*HCH_2_N_triazole_), 4.09 (m, 3H, OCH*H*CH_2_N_triazole_), 3.90 (m, 3H, H-4), 3.78−3.31 (m, 39H, H-3, H-5, H-2, H-6, CCH_2_O, CC*H*_*2*_OCH_2_CH_2_, OCH_2_, OCH_2_C*H*_*2*_NHCO), 3.16 (m, 2H, Hc), 2.52 (m, 4H, Hd, He), 1.59 (m, 2H, Hb), 0.65 (m, 2H, Ha); ^13^C NMR (100 MHz, D_2_O) *δ*: 174.6 (CO), 144.1 (C_triazole_), 125.6 (CH_triazole_), 103.1 (C-1), 75.1 (C-5), 72.7 (C-4), 70.6 (C-2), 69.6, 69.5, 69.4, 69.1, 68.9, 68.5 (C-3), 68.3, 68.0 (O*C*H_2_CH_2_N_triazole_), 63.6 (O*C*H_2_C_triazole_), 60.9 (C-6), 50.3 (OCH_2_*C*H_2_N_triazole_), 44.3 (*C*CH_2_O), 41.8 (Cc), 38.9 (OCH_2_*C*H_2_NHCO), 31.2 (Cd, Ce), 22.5 (Cb), 9.7 (Ca).

## Supporting Information Available

Spectral and other characterization data for all compounds and glyconanomaterials; additional experimental details, materials, and methods, including photographs of the experimental setup; additional figures of related biological studies (PDF)
