# Supplementary material for: Glycan-Silica Nanoparticles as Effective Inhibitors for Blocking Virus Infection
Source: ACS Appl Mater Interfaces. 2025 Feb 5;17(7):10292–304. doi: 10.1021/acsami.4c15918 (PMC11881045; doi:10.1021/acsami.4c15918)
Supplement: Supplementary file 1 — am4c15918_si_001.pdf [file am4c15918_si_001.pdf]

Supporting Information

Glycan-silica nanoparticles as effective inhibitors for blocking virus infection

Carmen Pérez-Alonso,[a] Fátima Lasala,[b] Laura Rodríguez-Pérez,[c] Rafael Delgado,[b,*] Javier Rojo,[a,*] Javier Ramos-Soriano[a,*]

[a]*Glycosystems Laboratory Instituto de Investigaciones Químicas (IIQ) CSIC – Universidad de Sevilla, Av. Américo Vespucio 49, 41092 Seville, Spain.*

[b]*Laboratorio de Microbiología Molecular Instituto de Investigación Hospital 12 de Octubre (imas12), 28041 Madrid, Spain.*

[c]*Departamento de Química Orgánica, Facultad de Química, Universidad Complutense, 28040 Madrid, Spain.*

*Corresponding authors: rafael.delgado@salud.madrid.org; javier.rojo@iiq.csic.es; fj.ramos@iiq.csic.es

1.	General ………..……………………………………………………………...…..	S2
2.	General procedure for synthesis of glycodendrons **14-16**..…………………………	S2
3.	^{1}H and ^{13}C NMR spectra……………………………………………………….	S3
4.	Zeta potential…………………………………………………………………..	S16
5.	DLS…………………………………………………………………………….	S16
6.	TGA analysis…………………………………………………………………..	S17
7.	Infrared………………………………………………………………………...	S18
8.	XPS analysis…………………………………………………………………...	S19
9.	Determination of the amount of carbohydrates in the glycoSiNPs by UV-Vis……..	S23
10.	Lectin (ConA) binding assays by DLS……………………………………………	S24
11.	Lectin (ConA) binding assays by turbidimetry assays………………………………	S27
12.	Biological assays……………………………………………………………….	S30
13.	Reference..……………………………………………………………………..	S32

1. General

Reagents and solvents were purchased as reagent grade and used without further purification. H_2O was purified with a Milli-Q purification system from Millipore (18.3 Ω). Thin layer chromatography (TLC) analyses were performed on silica gel 60 F_{254} precoated on aluminium plates (Merck), with detection by UV light (λ = 254 or 365 nm) and charred with Moustain, potassium permanganate or anisaldehyde as development reagents. Column chromatography was carried out on silica gel 60 (0.040–0.063 mm or 0.015–0.04 mm, Merck) or by Sephadex LH20 or G25 (GE Healthcare, Barcelona, Spain) gel filtration. ^{1}H and ^{13}C NMR spectra were obtained for solutions in deuterated solvents (indicated in each case) at 298K on Bruker DRX400 MHz NMR spectrometer with solvent peaks as reference. All chemical shifts were reported in ppm (δ) and coupling constants (J) are reported in Hertz (Hz). All the assignments were confirmed by one- and two-dimensional NMR experiments (COSY and HSQC). Electrospray mass spectra (ESI MS) were registered at the Mass Spectrometry Service of the Institute for Chemical Research (IIQ, CSIC-US) using an Elute UHPLC system coupled to a Bruker Amazon SL spectrometer instrument. High resolution mass spectra (HR MS) were carried out at Mass Spectrometry Service of CITIUS (University of Seville) using a Thermo Scientific Orbitrap Elite. Microwave irradiation experiments were performed using a Biotage© Initiator EXP US Microwave Reactor System Robot Sixty/Eight apparatus. The temperature in the sealed reaction vessel was monitored by an external surface sensor. IR measurements was performed in a JASCO FT/IR-6200 IRT-5000 and platinum ATR Bruker tensor 27. DLS and zeta potential experiments was carried out in a Malvern Nano ZS (Malvern Instruments, U.K.) operating at 633 nm with a 173º scattering angle. Measurements were carried out three times and made in a quartz 10 mm path cuvette for size analysis and in a semi-micro 10 mm path-length PMMA cuvettes maintained at 25ºC for turbidimetry assays. For zeta potential data a special electrode cuvette was used. UV-Vis for carbohydrate amount determination and turbidimetry assays was done in a UV-Vis spectrometer Jasco V-650 at 25°C using a 1cm path quart cuvette for both measurements. TGA analyses were carried out under nitrogen in a TA/TGA-Q500 apparatus. The sample (~0.5 mg) was introduced inside a platinum crucible and equilibrated at 90 ºC followed by a 10 ºC min^{-1} ramp between 90 and 1000 ºC. XPS analysis was carried out using a SPECS GmbH (PHOIBOS 150 9MCD) spectrometer operating in the constant analyzer energy mode. A non-monochromatic aluminium X-ray source (1486.61 eV) was used with a power of 200 W and voltage of 12 kV. Pass energies of 75 and 25 eV were used for acquiring both survey and high-resolution spectra, respectively. Survey data were acquired from kinetic energies of 1487-400 eV with an energy step of 1 eV and 100 ms dwell time per point. The high-resolution scans were taken around the emission lines of interest with 0.1 eV steps and 100 ms dwell time per point. SpecsLab Version 2.48 software was used for spectrometer control and data handling. The semi-quantitative analysis was performed from the C 1s (284.6 eV) signal. The samples were introduced as pellets of 8 mm diameter.

2. General procedure for synthesis of glycodendrons 14-16.

To a solution of azide derivative **6**, **8** or **10** (3.6 eq.) and [2-[2-(2-aminoethoxy)ethoxy]ethoxy]ethoxymethyl trikis(2-propyniloxy-methyl)methane (**13**) (1 eq.) in H_2O/DMSO (1:1) in a sealed microwave vial, $CuSO_4 \cdot 5H_2O$ (0.3 eq), TBTA (0.6 eq.) and sodium ascorbate (0.9 eq.) were added. The solution was heated at 60 ºC in a microwave oven for 30 min. A metal scavenger resin, QuadrasilMP, was added to the reaction solution and stirred for 15 min at room temperature. The mixture was filtered and the resulting solution was purified by size-exclusion chromatography (Sephadex LH-20, MeOH 100%), furnishing the corresponding glycodendron **14-16**.

3. ^{1}H and ^{13}C NMR spectra

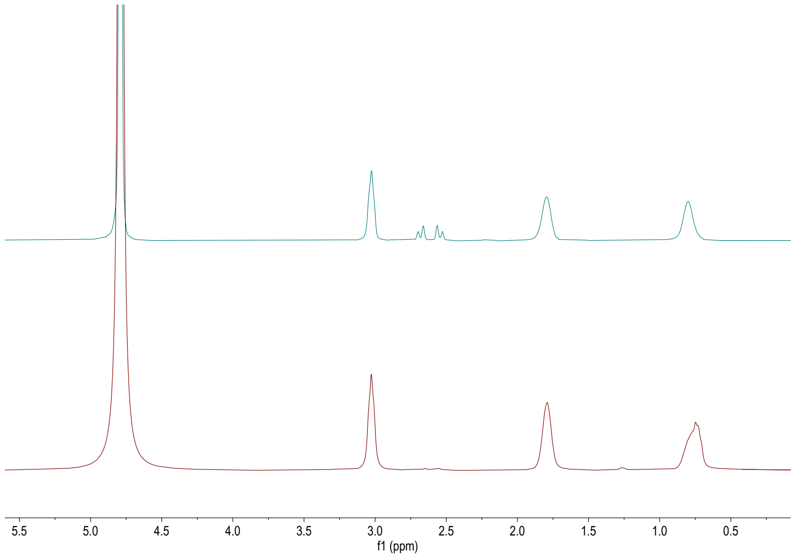

Figure S1. ^{1}H NMR spectrum of compound **NH_2@SiNPs** (D_2O, 400 MHz) before (top) and after (down) treatment with 1M NaOH solution.

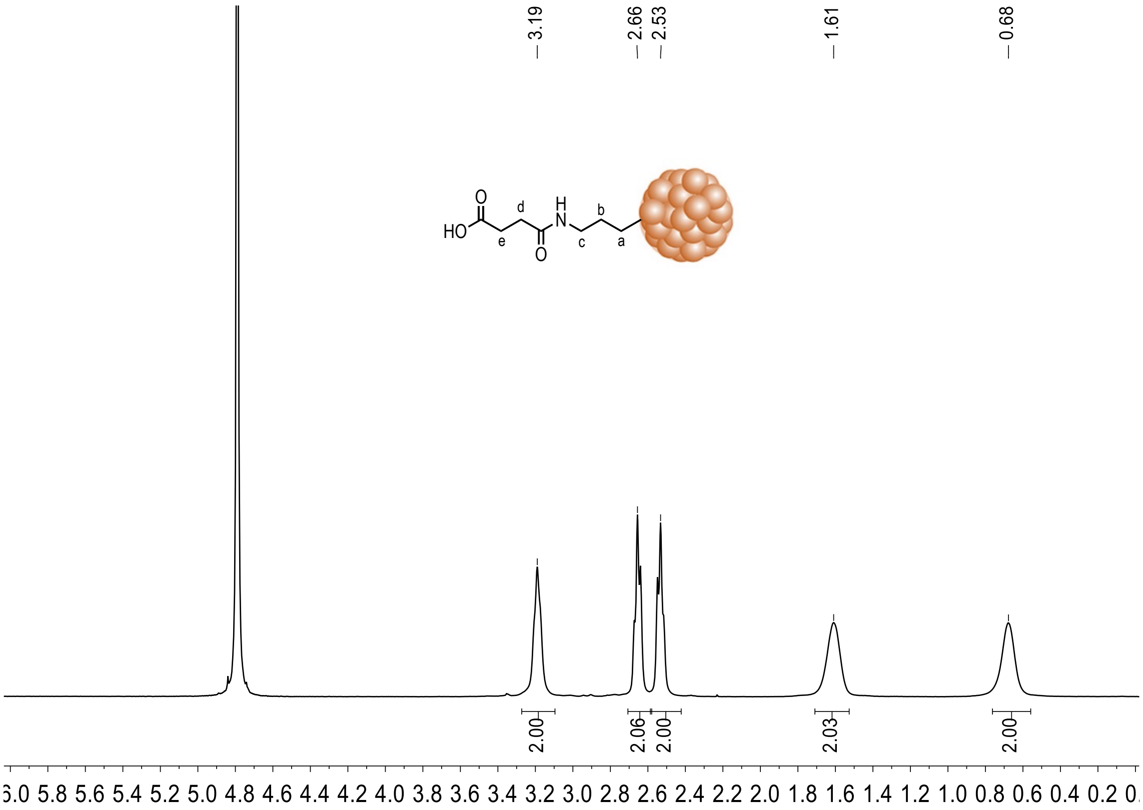

Figure S2. ^{1}H NMR spectrum of **COOH@SiNPs** (D_2O, 400 MHz).

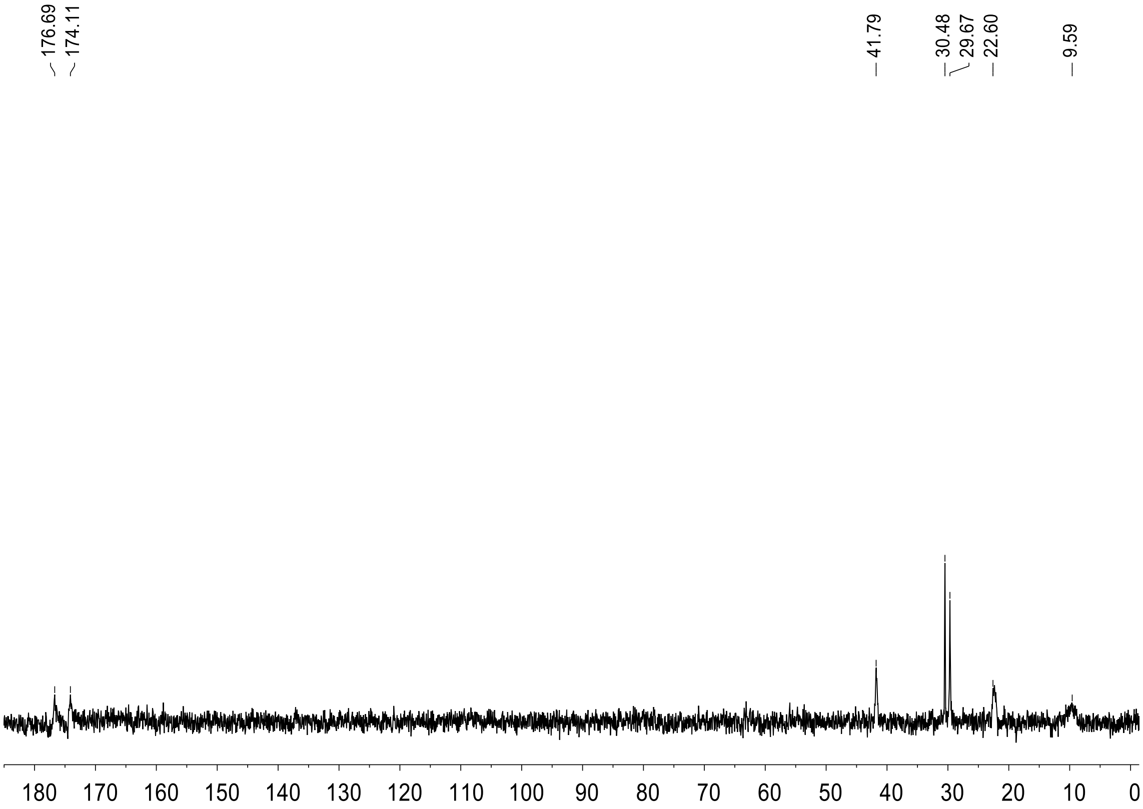

Figure S3. ^{13}C NMR spectrum of **COOH@SiNPs** (D_2O, 100 MHz)

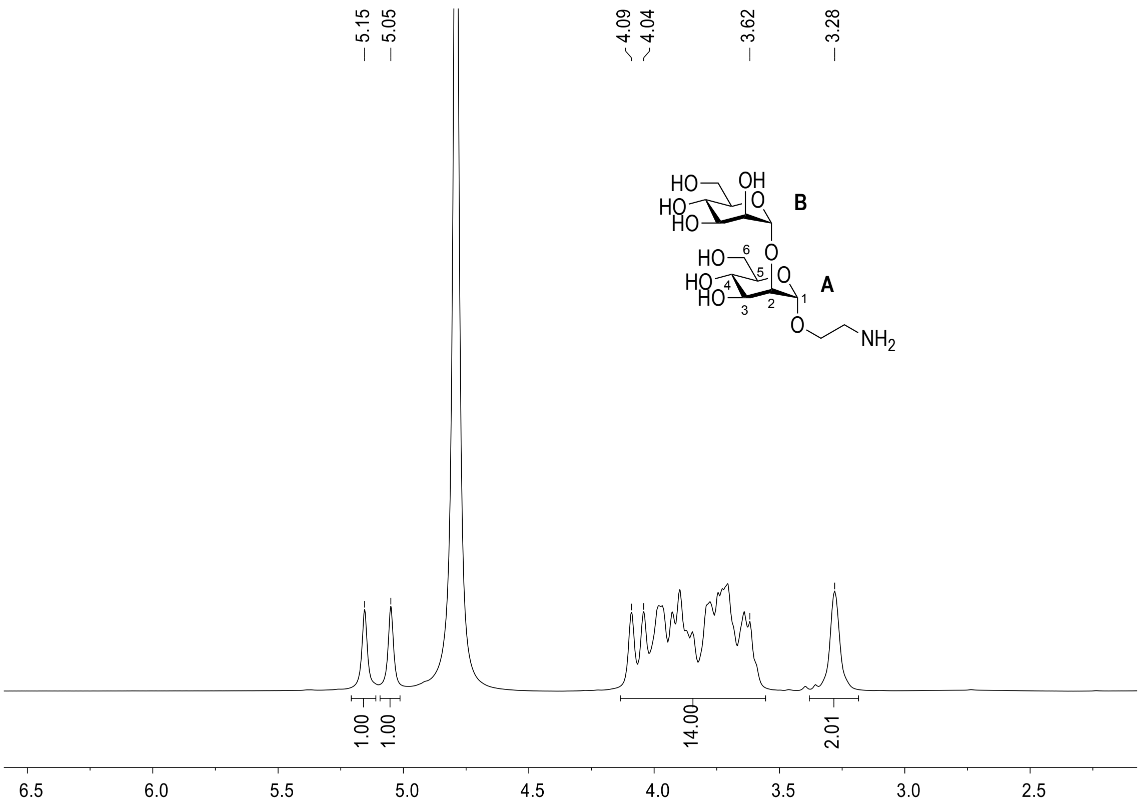

Figure S4. ^{1}H NMR spectrum of compound **9** (D_2O, 400 MHz).

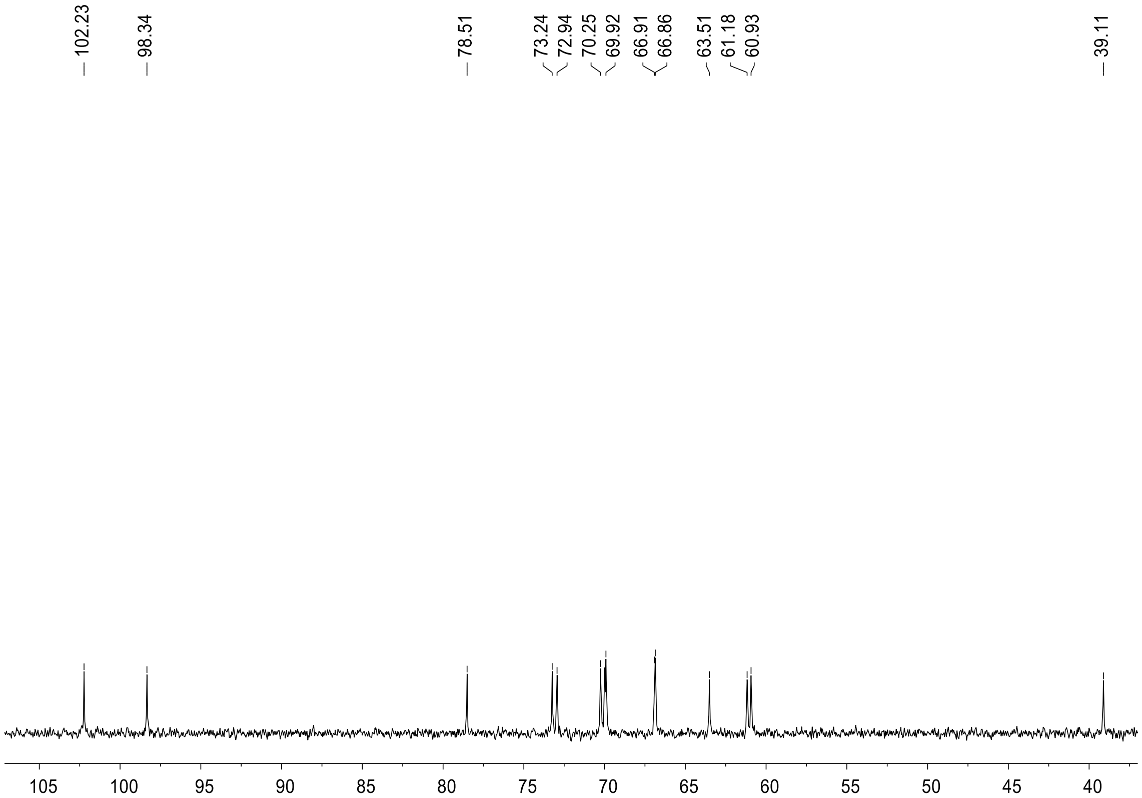

Figure S5. ^{13}C NMR spectrum of compound **9** (D_2O, 100 MHz).

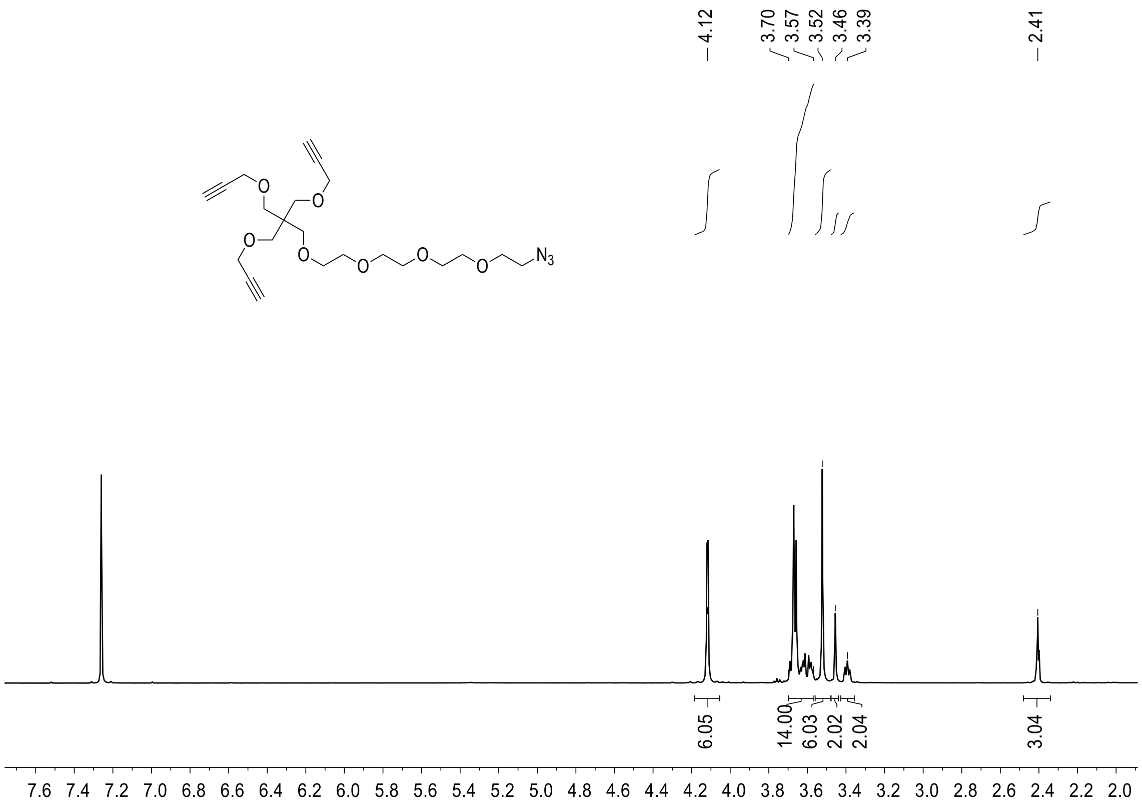

Figure S6. ^{1}H NMR spectrum of compound **12** ($CDCl_3$, 400 MHz).

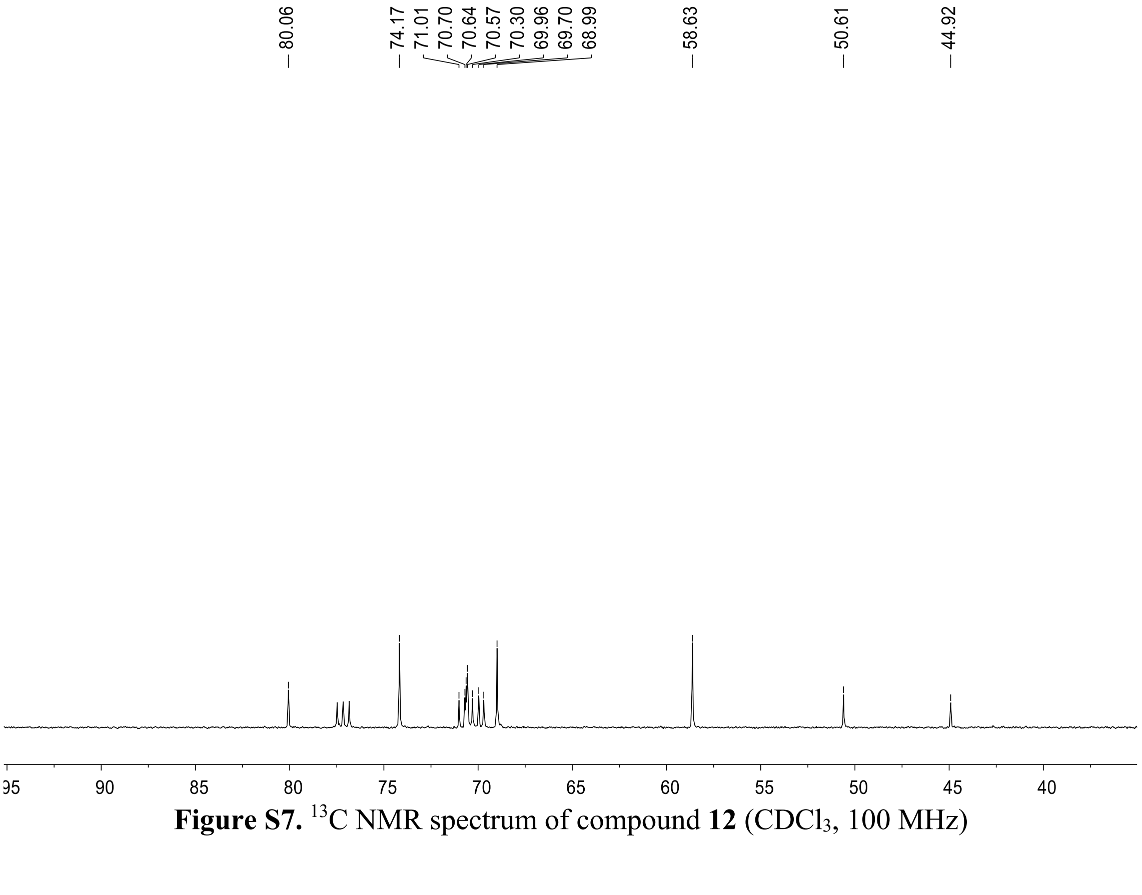

Figure S7. ^{13}C NMR spectrum of compound **12** ($CDCl_3$, 100 MHz)

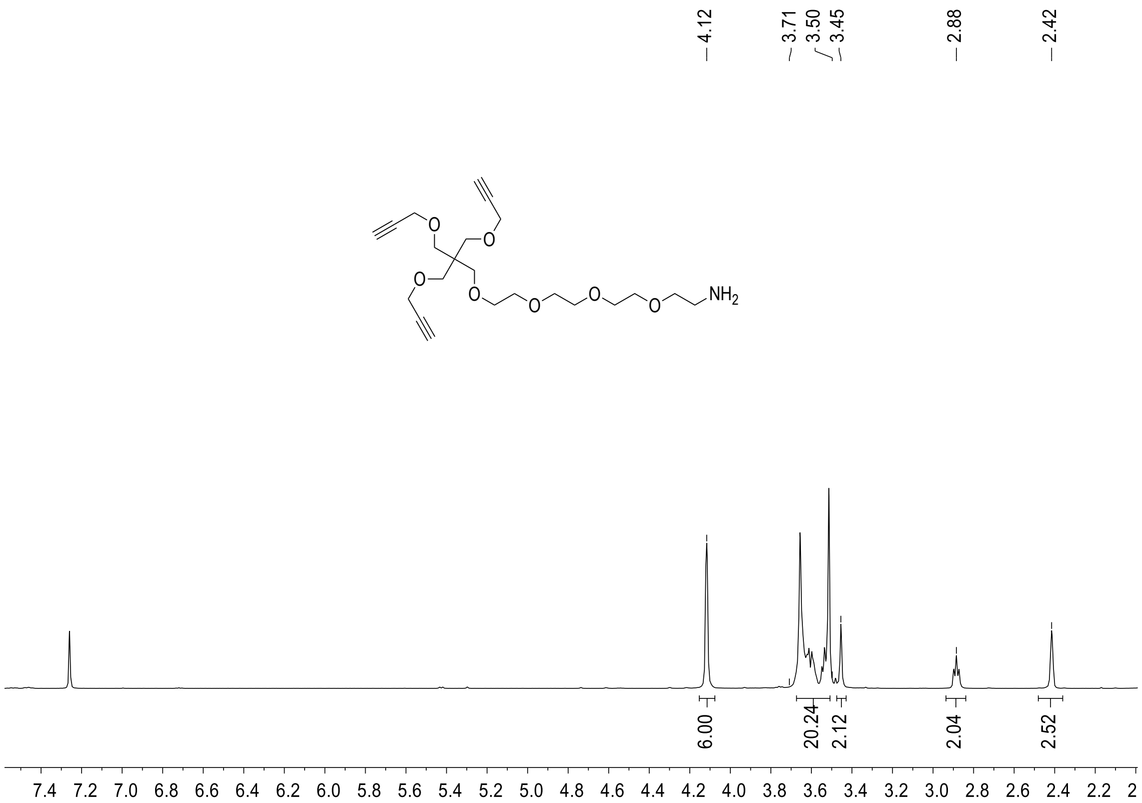

Figure S8. ^{1}H NMR spectrum of compound **13** ($CDCl_3$, 400 MHz).

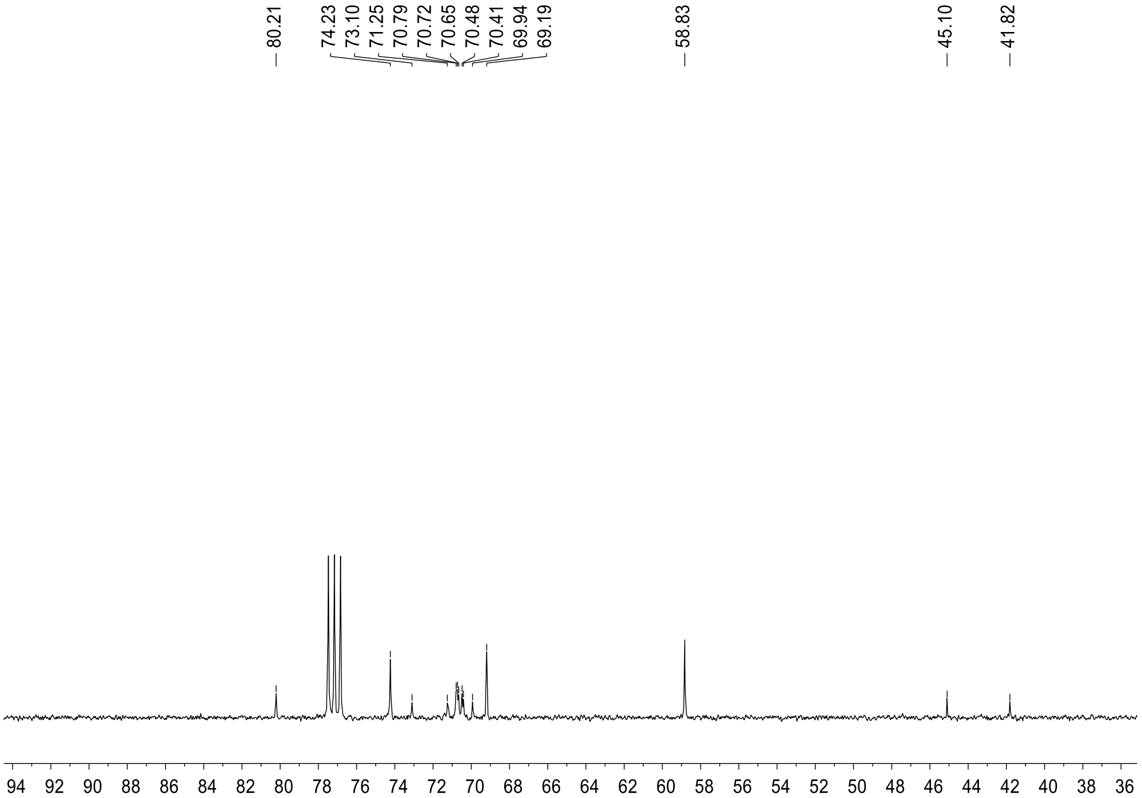

Figure S9. ^{13}C NMR spectrum of compound **13** ($CDCl_3$, 400 MHz).

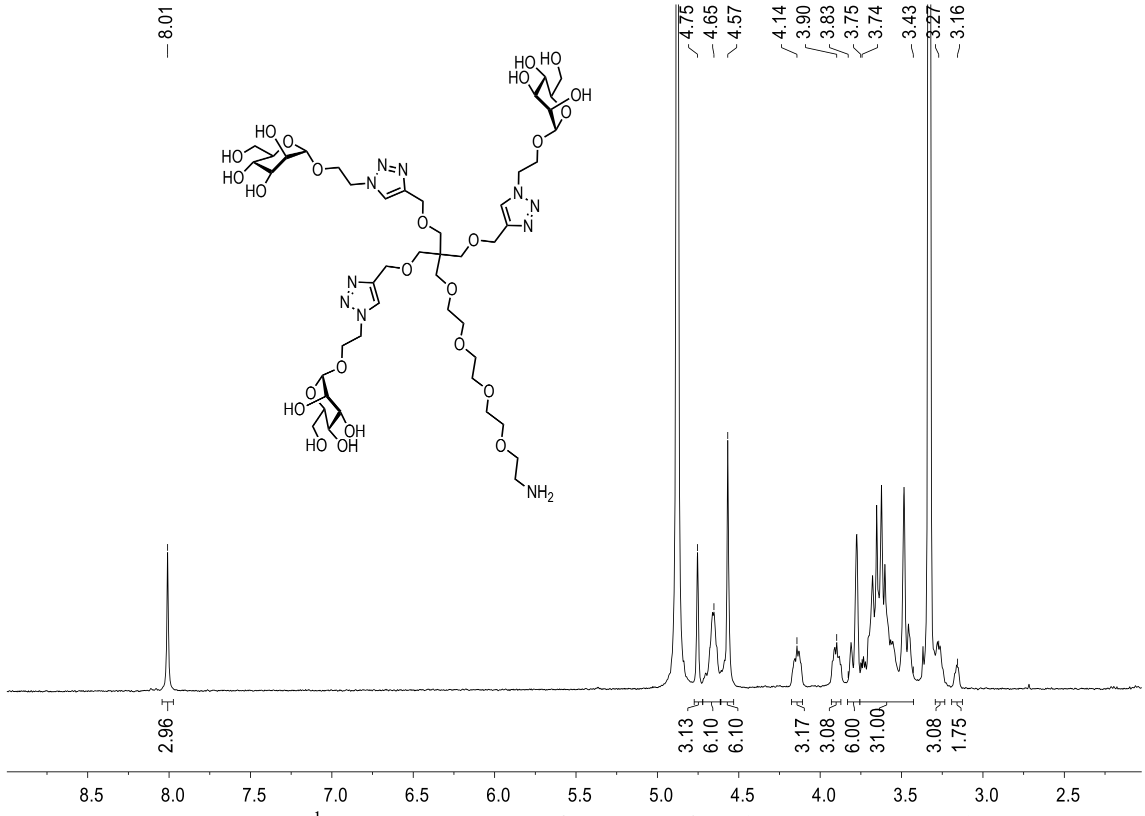

Figure S10. ^{1}H NMR spectrum of compound **14** (CD_3OD, 400 MHz).

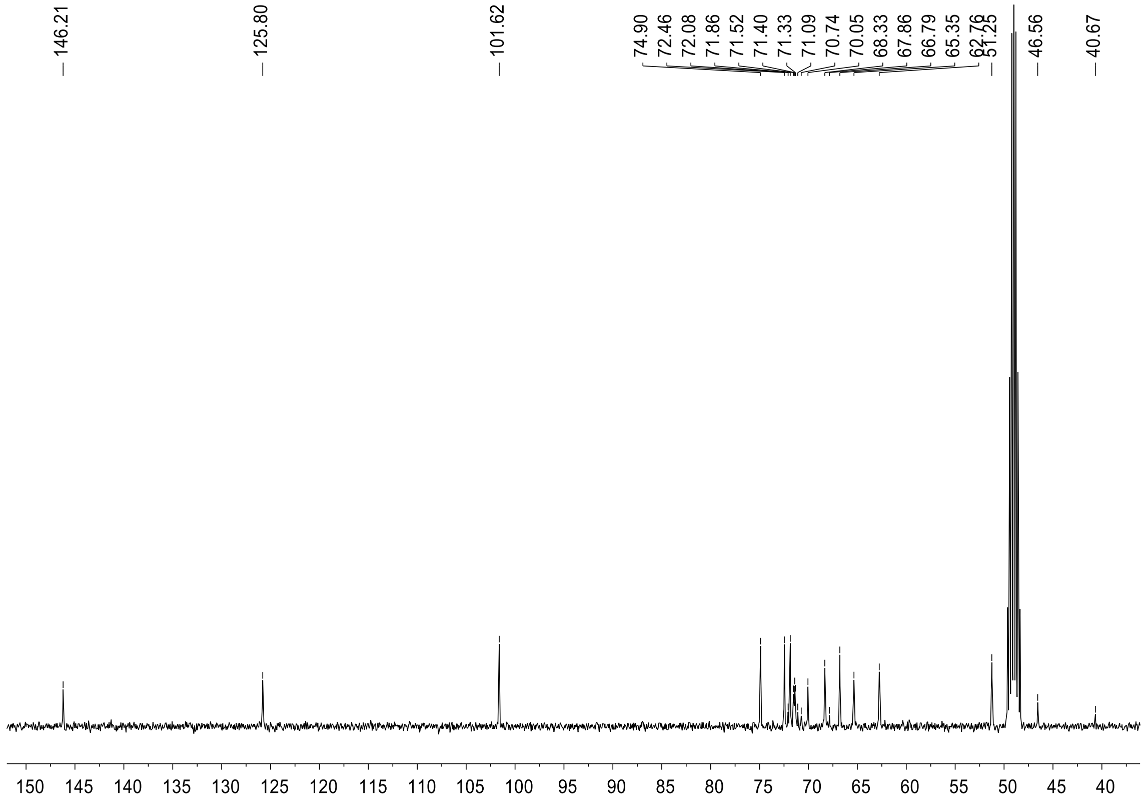

Figure S11. ^{13}C NMR spectrum of compound **14** (CD_3OD, 100 MHz).

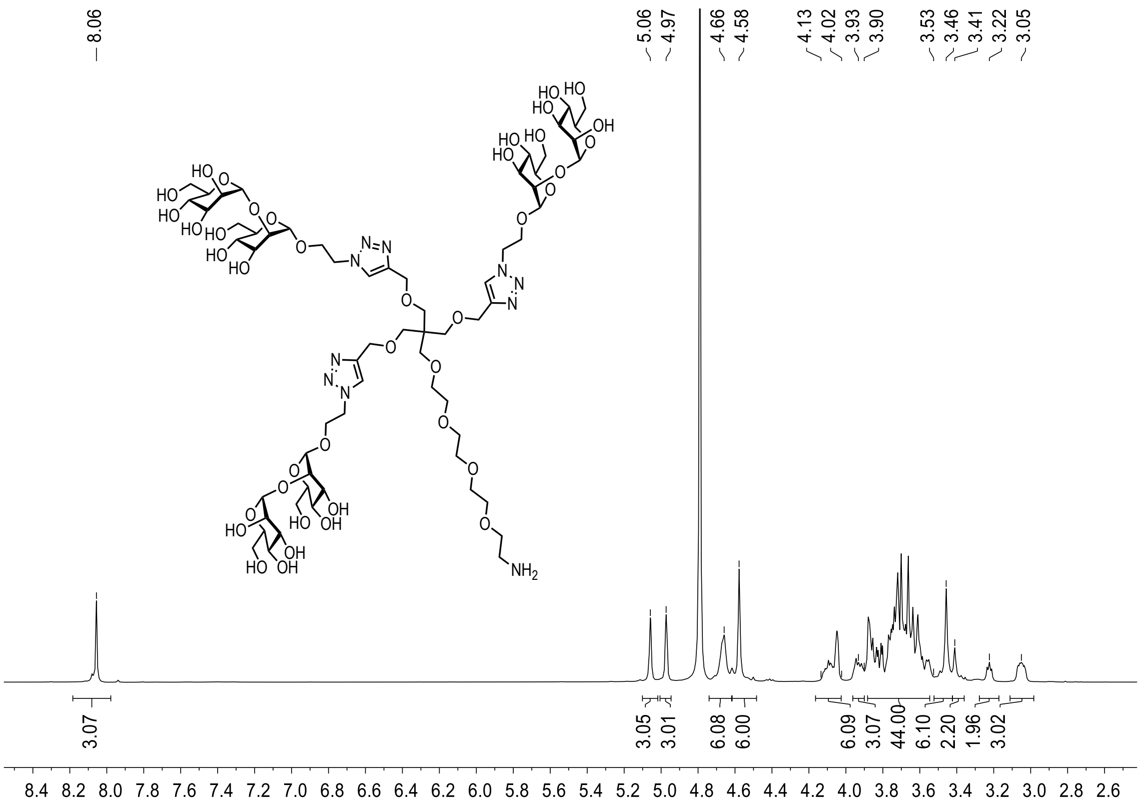

Figure S12. ^{1}H NMR spectrum of compound **15** (D_2O, 400 MHz).

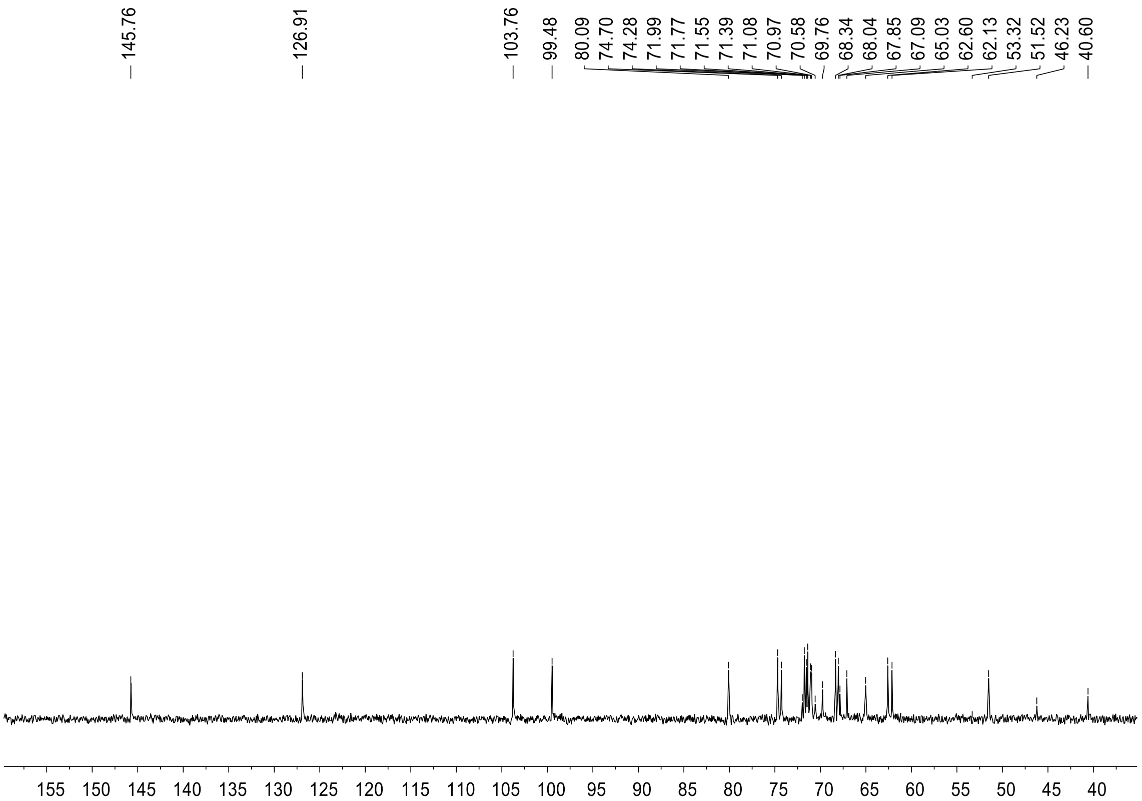

Figure S13. ^{13}C NMR spectrum of compound **15** (D_2O, 100 MHz).

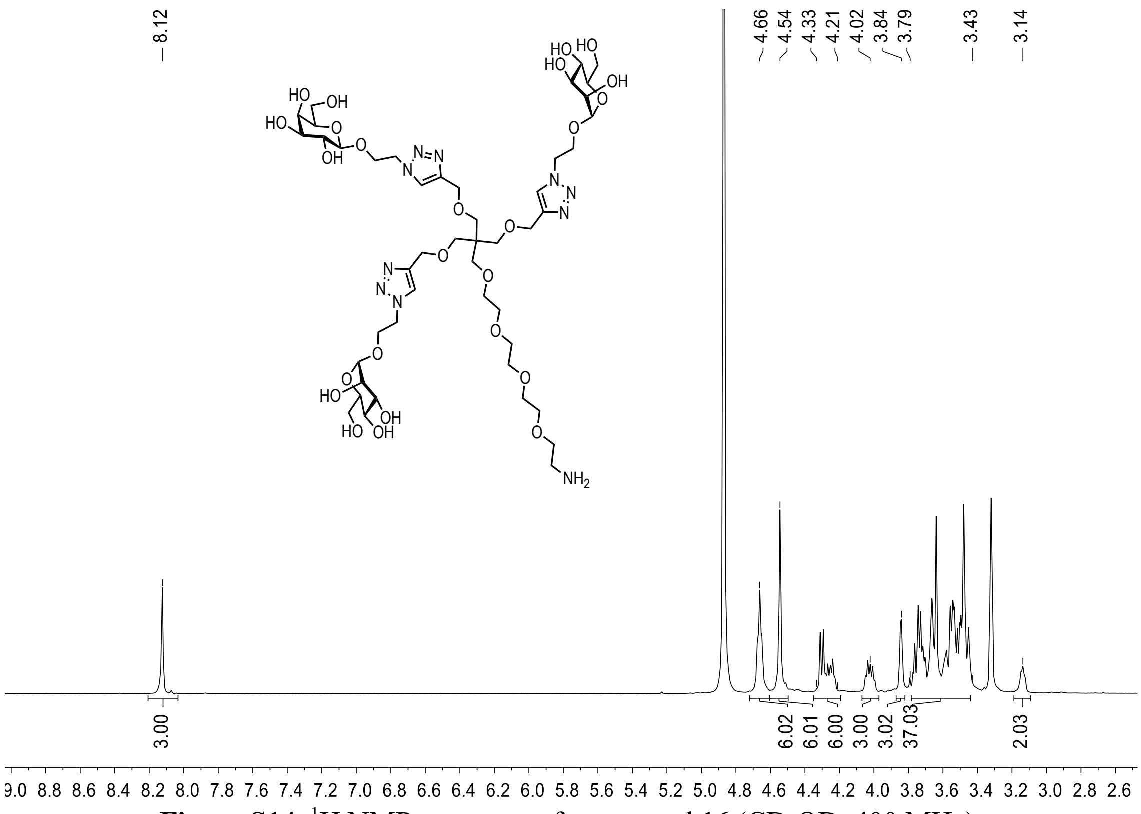

Figure S14. ^{1}H NMR spectrum of compound **16** (CD_3OD, 400 MHz).

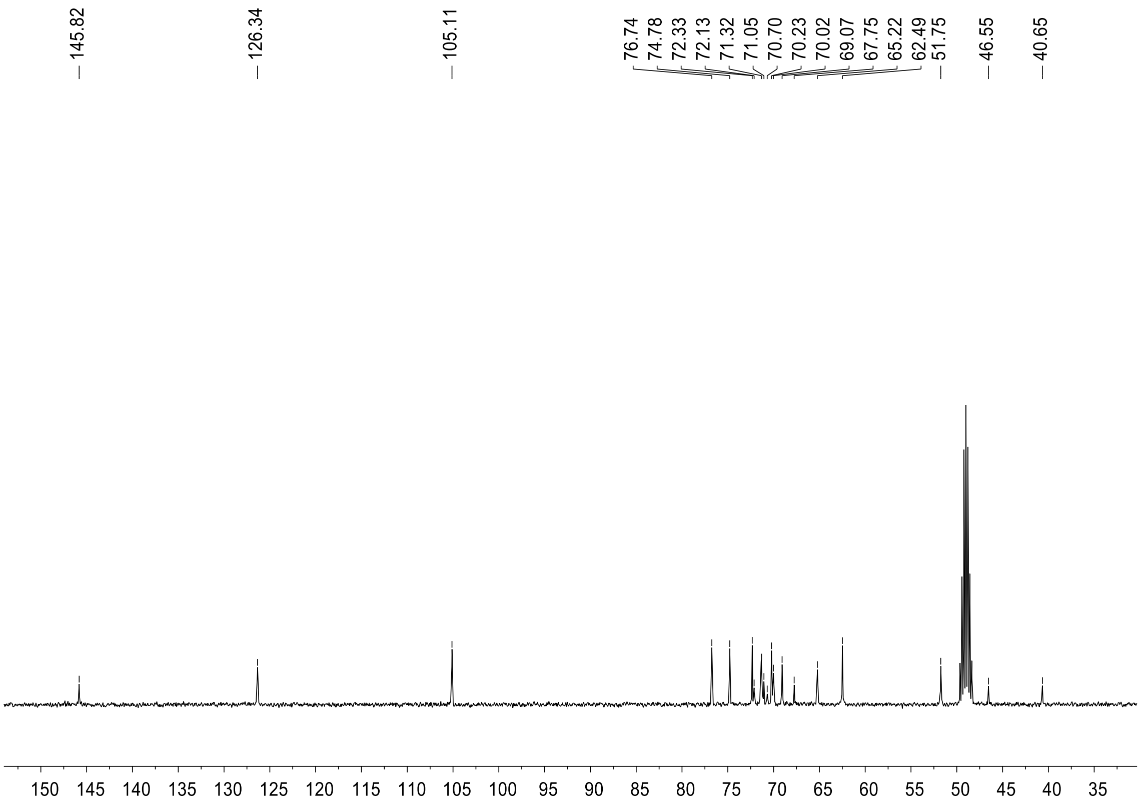

Figure S15. ^{13}C NMR spectrum of compound **16** (CD_3OD, 100 MHz).

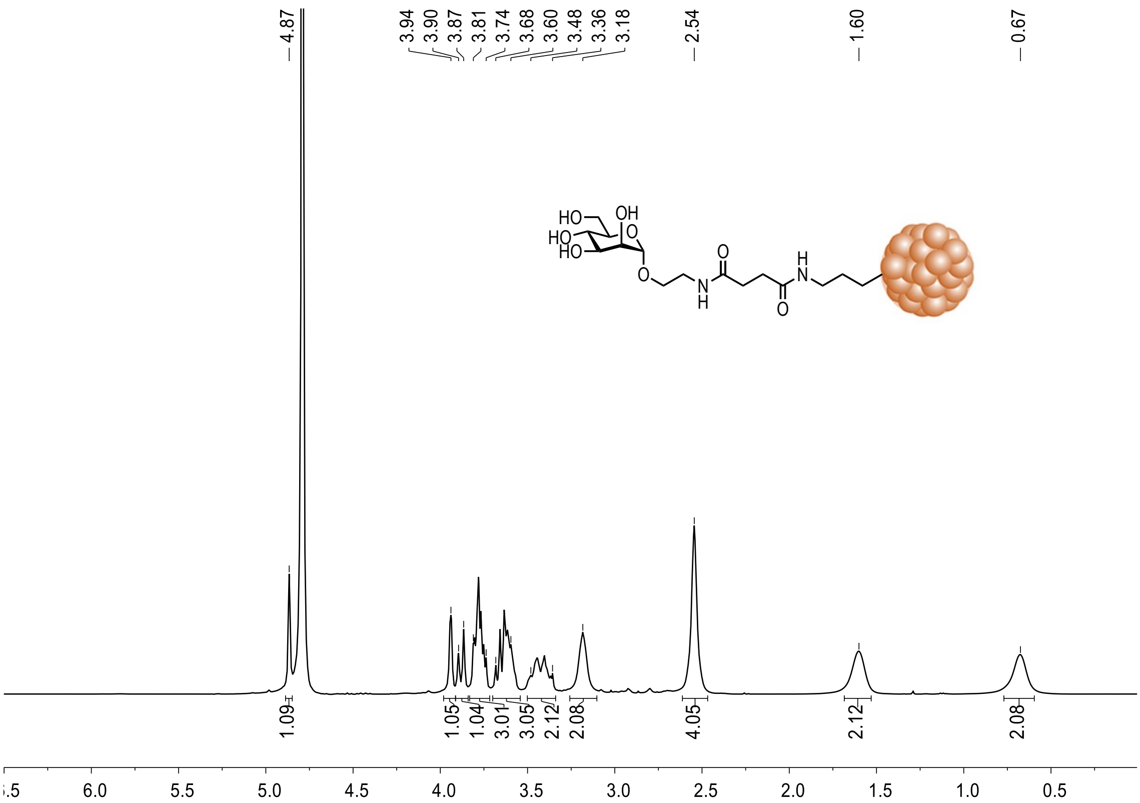

Figure S16. ^{1}H NMR spectrum of Man@SiNPs **1** (D_2O, 400 MHz).

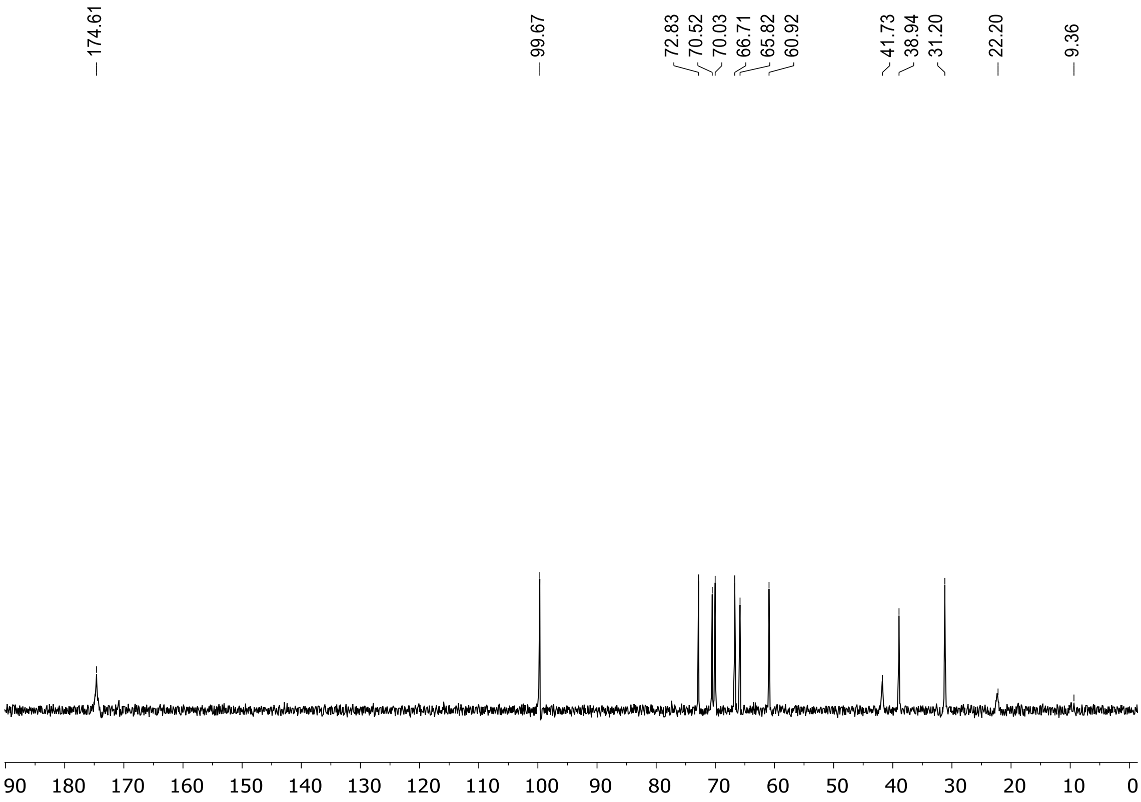

Figure S17. ^{13}C NMR spectrum of Man@SiNPs **1** (D_2O, 100 MHz).

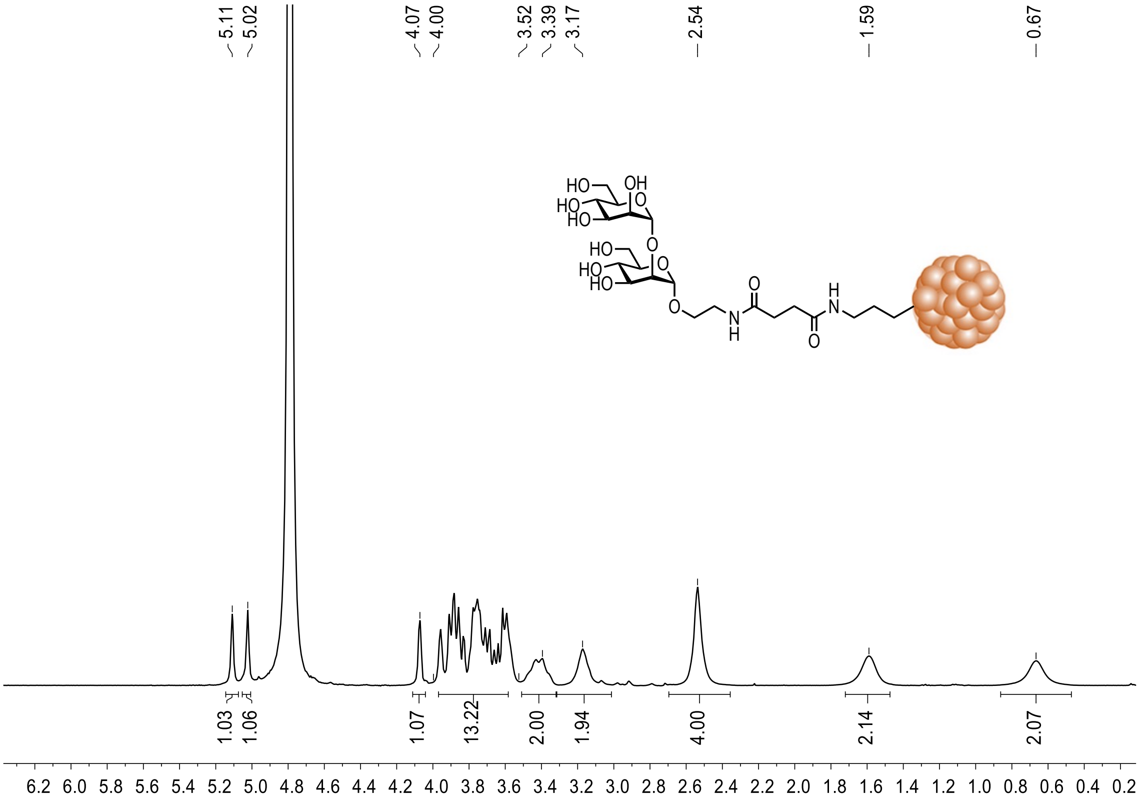

Figure S18. ^{1}H NMR spectrum of diMan@SiNPs **2** (D_2O, 400 MHz).

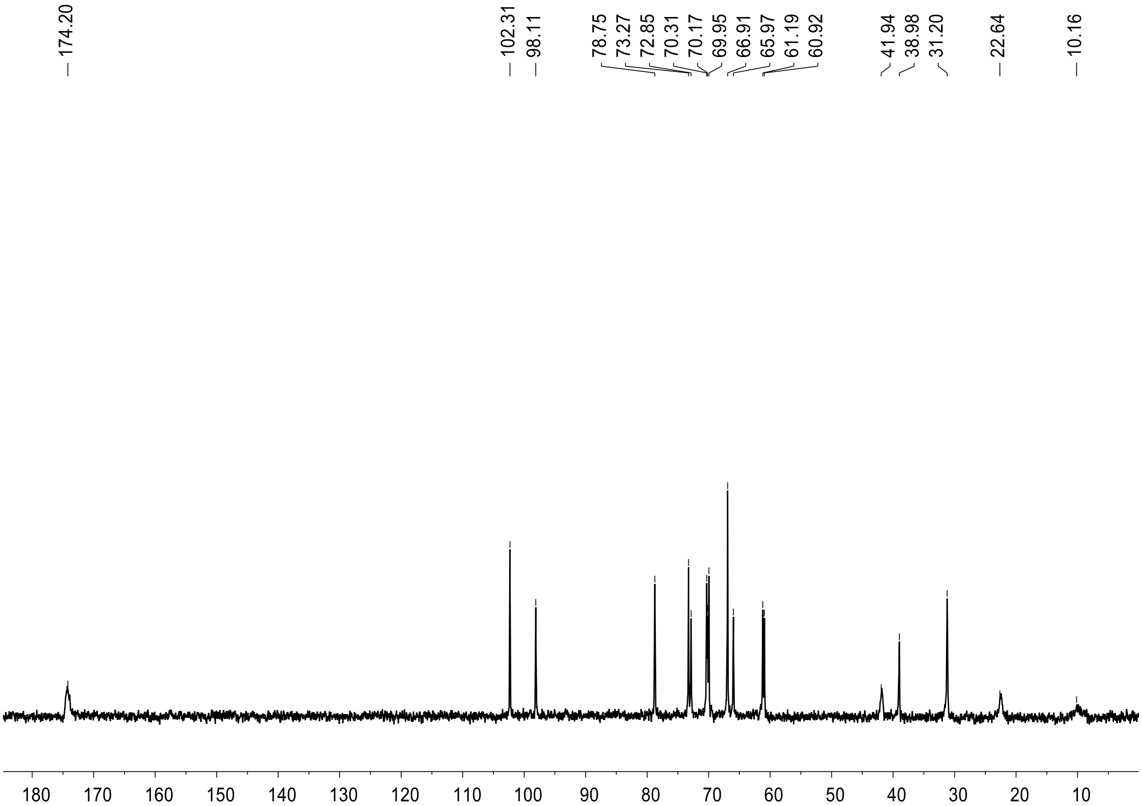

Figure S19. ^{13}C NMR spectrum of diMan@SiNPs **2** (D_2O, 100 MHz).

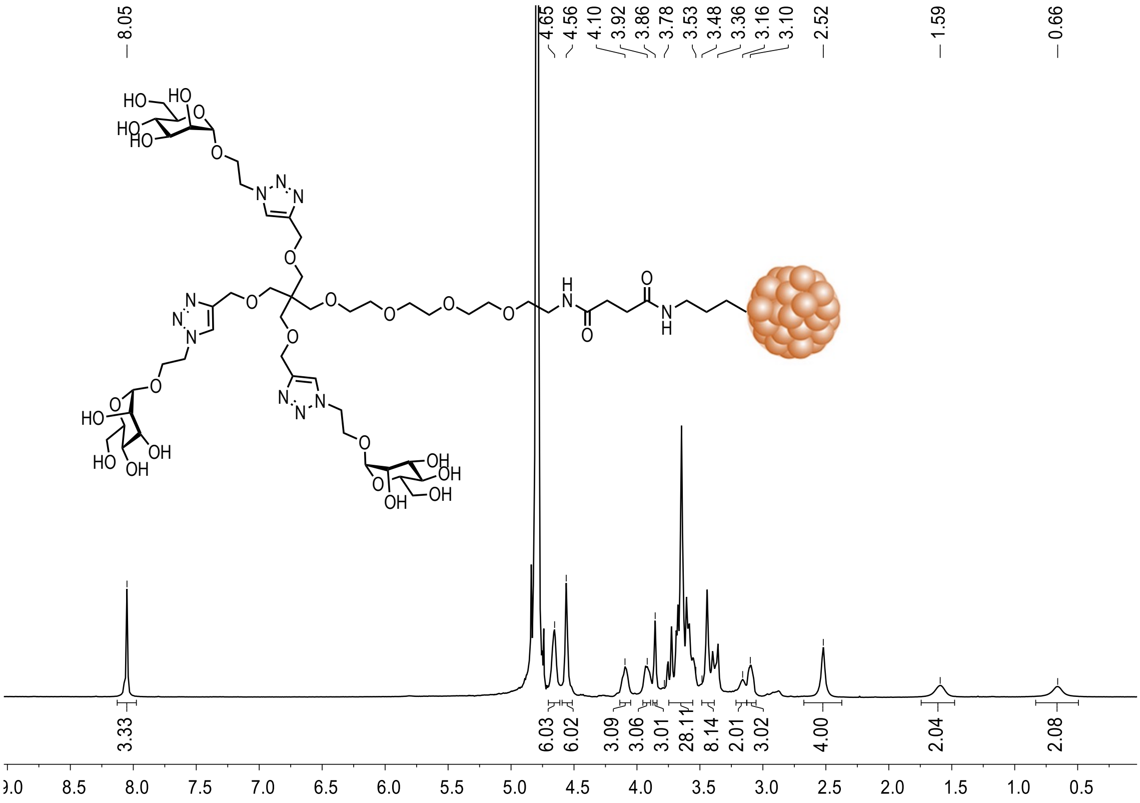

Figure S20. ^{1}H NMR spectrum of Man_3@SiNPs **3** (D_2O, 400 MHz).

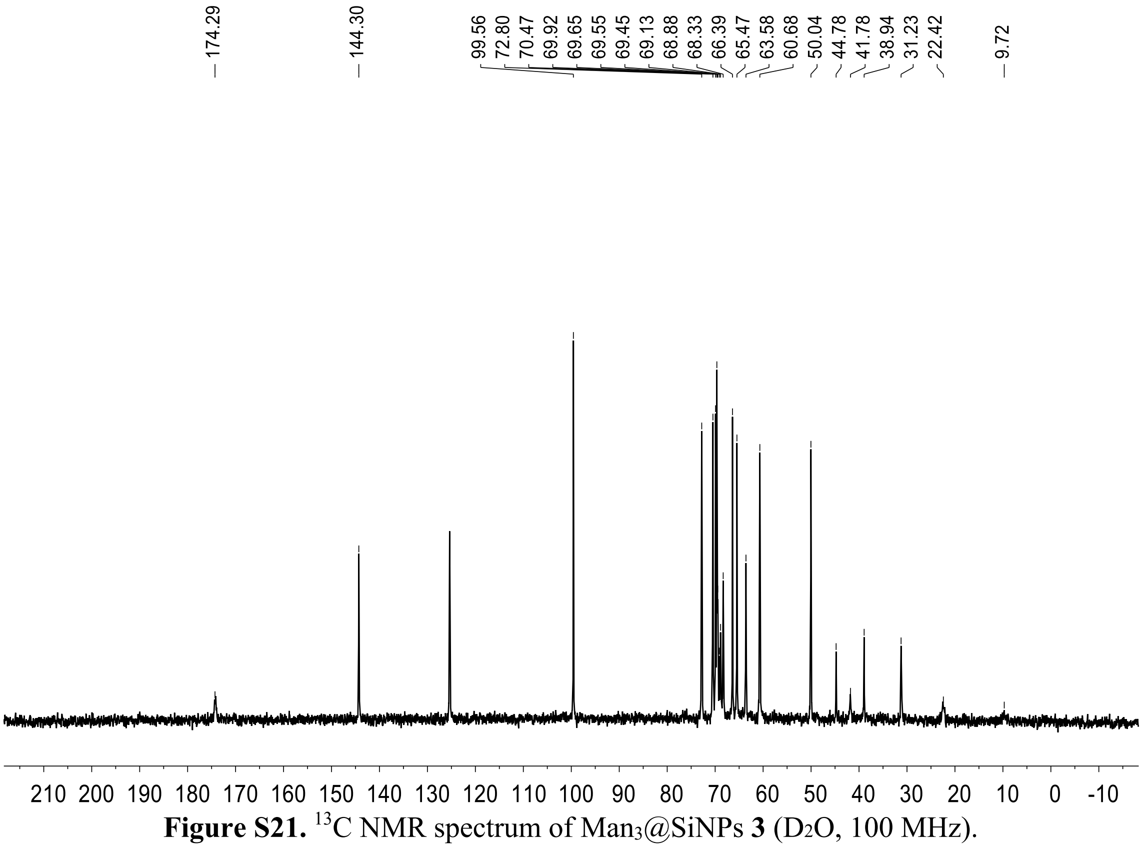

Figure S21. ^{13}C NMR spectrum of Man_3@SiNPs **3** (D_2O, 100 MHz).

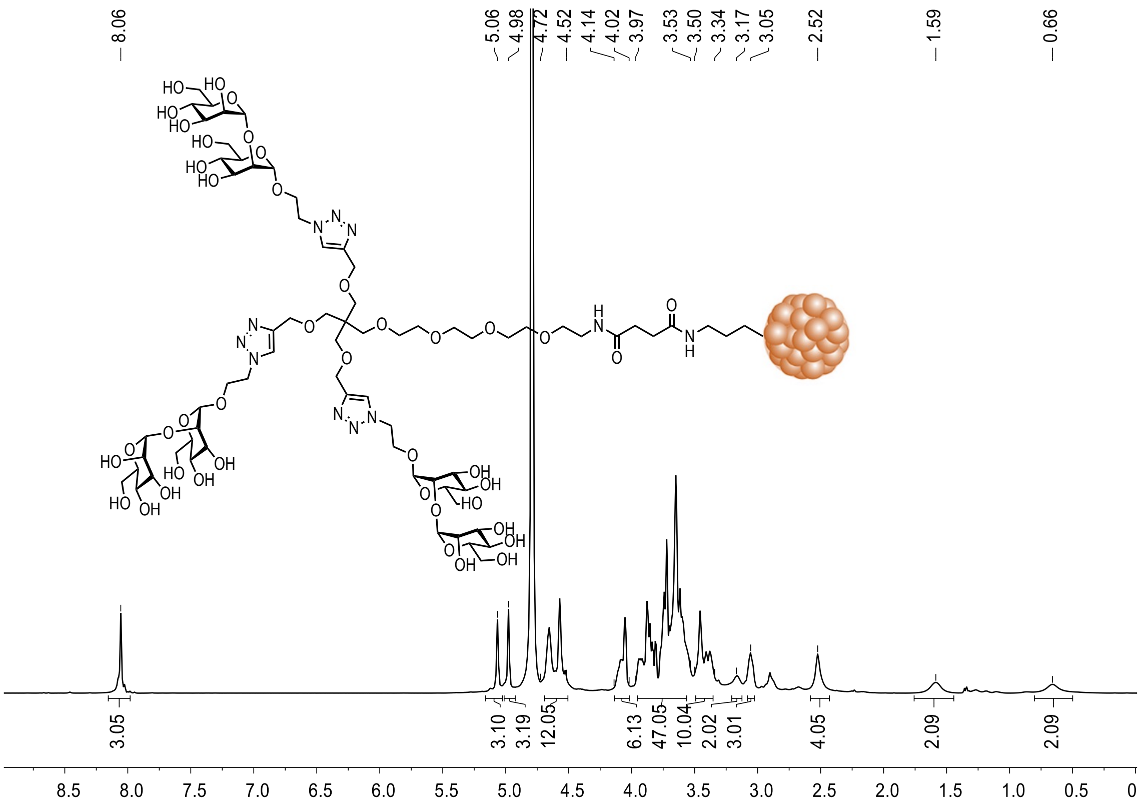

Figure S22. ^{1}H NMR spectrum of diMan$_3$@SiNP **4** (D_2O, 400 MHz).

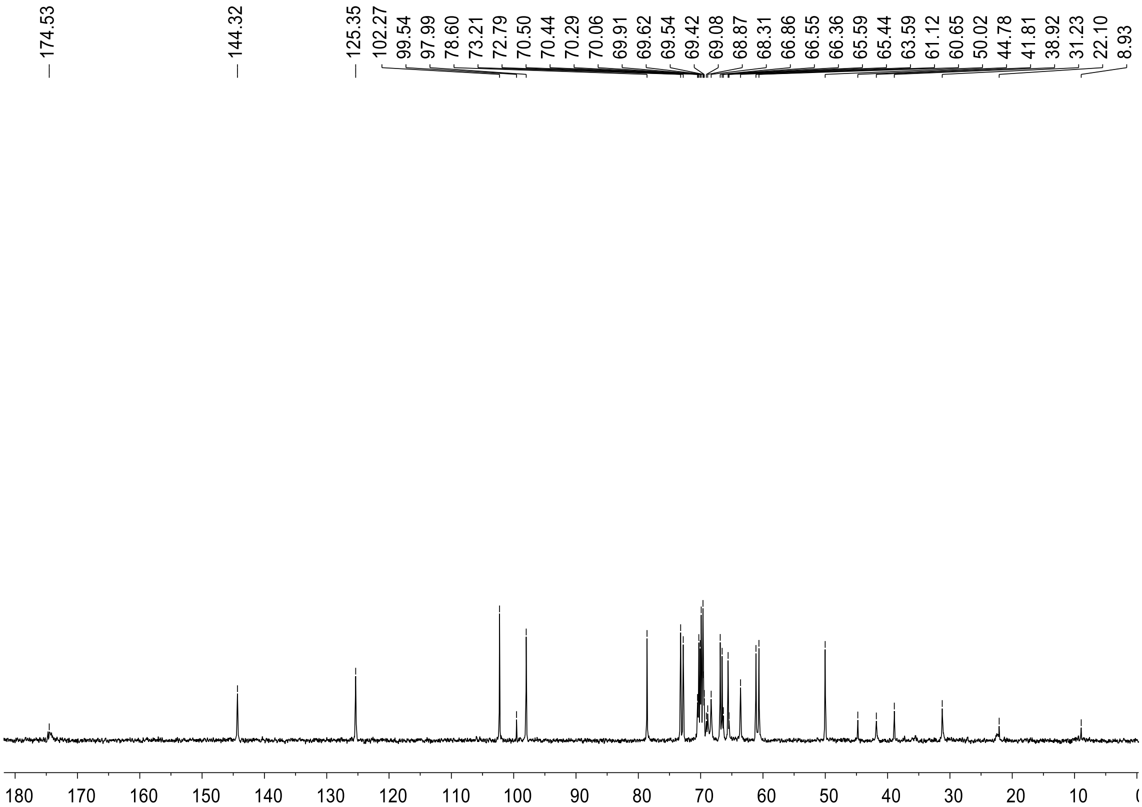

Figure S23. ^{13}C NMR spectrum of diMan$_3$@SiNPs **4** (D_2O, 100 MHz).

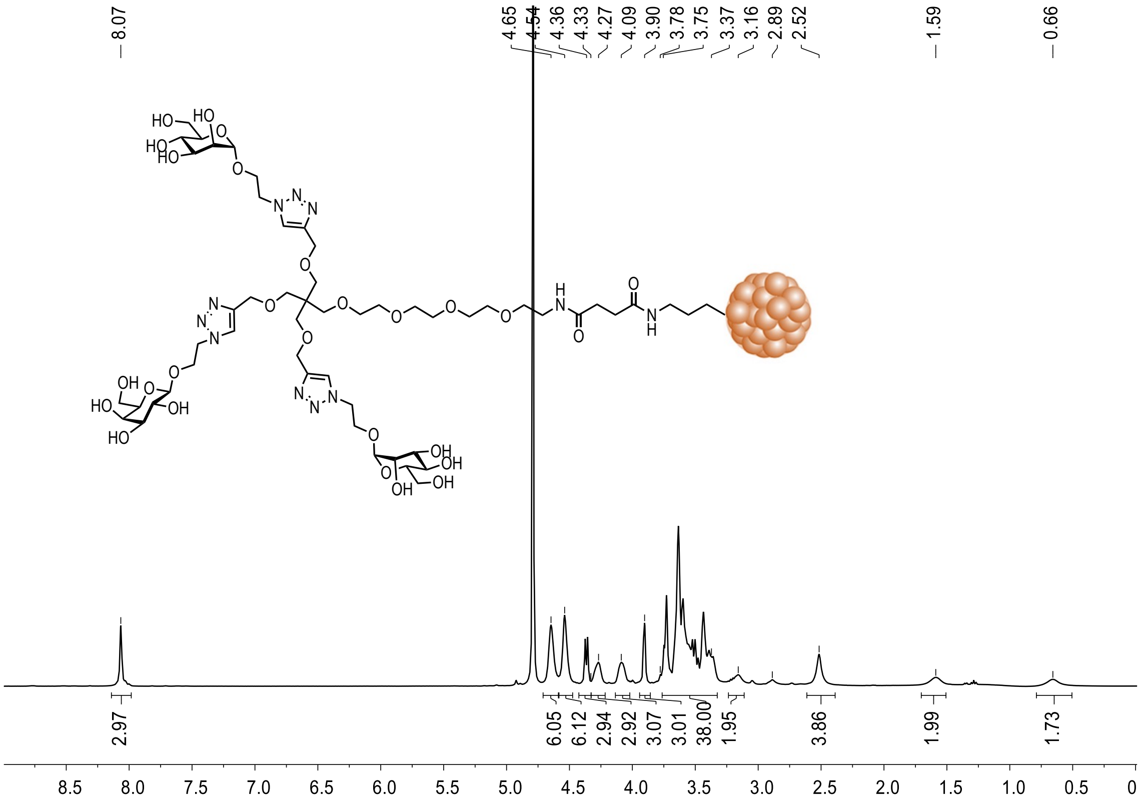

Figure S24. ^1H NMR spectrum of $Gal_3@SiNPs$ **5** (D_2O, 400 MHz).

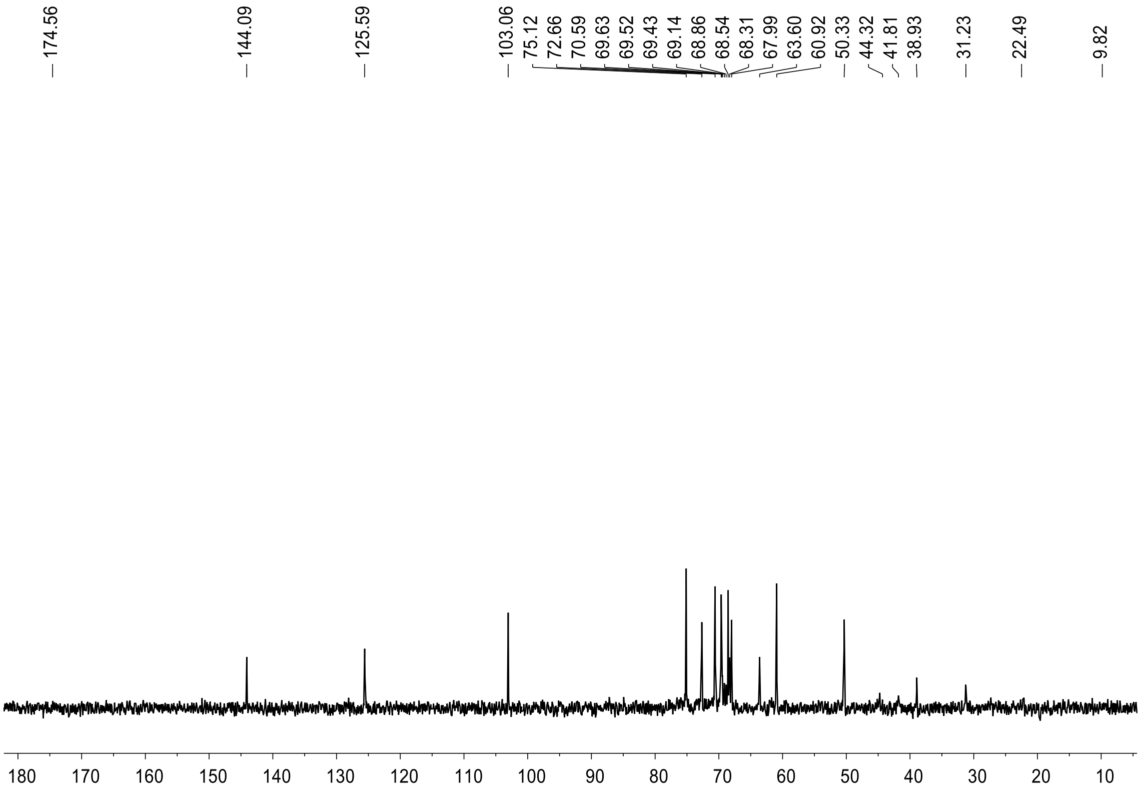

Figure S25. ^{13}C NMR spectrum of $Gal_3@SiNPs$ **5** (D_2O, 100 MHz).

4. Zeta potential

Malvern Nano ZS (Malvern Instruments, U.K.) operating at 633 nm with a 173º scattering angle. instrument was also used to measure the zeta potential. The Helmholtz-Smoluchowski equation was used to correlate the measured electrophoretic mobilities to the zeta potentials. Three replicates of each sample (1 mg/mL in miliQ H_2O) were measured at 298K.

Table S1. Zeta potential distributions

SiNPs	Zeta potential (mV)
**NH_2@SiNPs**	15.7 ± 8.25
**COOH@SiNPs**	-21.5 ± 4.05
**Man@SiNPs (1)**	-9.46 ± 4.30
**diMan@SiNPs (2)**	-12.6 ± 10.0
**Man_3@SiNPs (3)**	-19.4 ± 9.60
**diMan_3@SiNPs (4)**	-25.5 ± 11.70
**Gal_3@SiNPs (5)**	-26.5 ± 7.91

5. DLS

For glycoSiNPs **1-5,** DLS measurements were performed on a Malvern Nano ZS (Malvern Instruments, U.K.) operating at 633 nm with a 173º scattering angle. Measurements were made in a 1 cm path-lenght round quartz cell maintained at 298K. Freshly prepared 1 mg/mL solution samples in MilliQ water were filtered through a 0.45 µm pore size nylon filter before the measurements. Three replicates of each sample were measured.

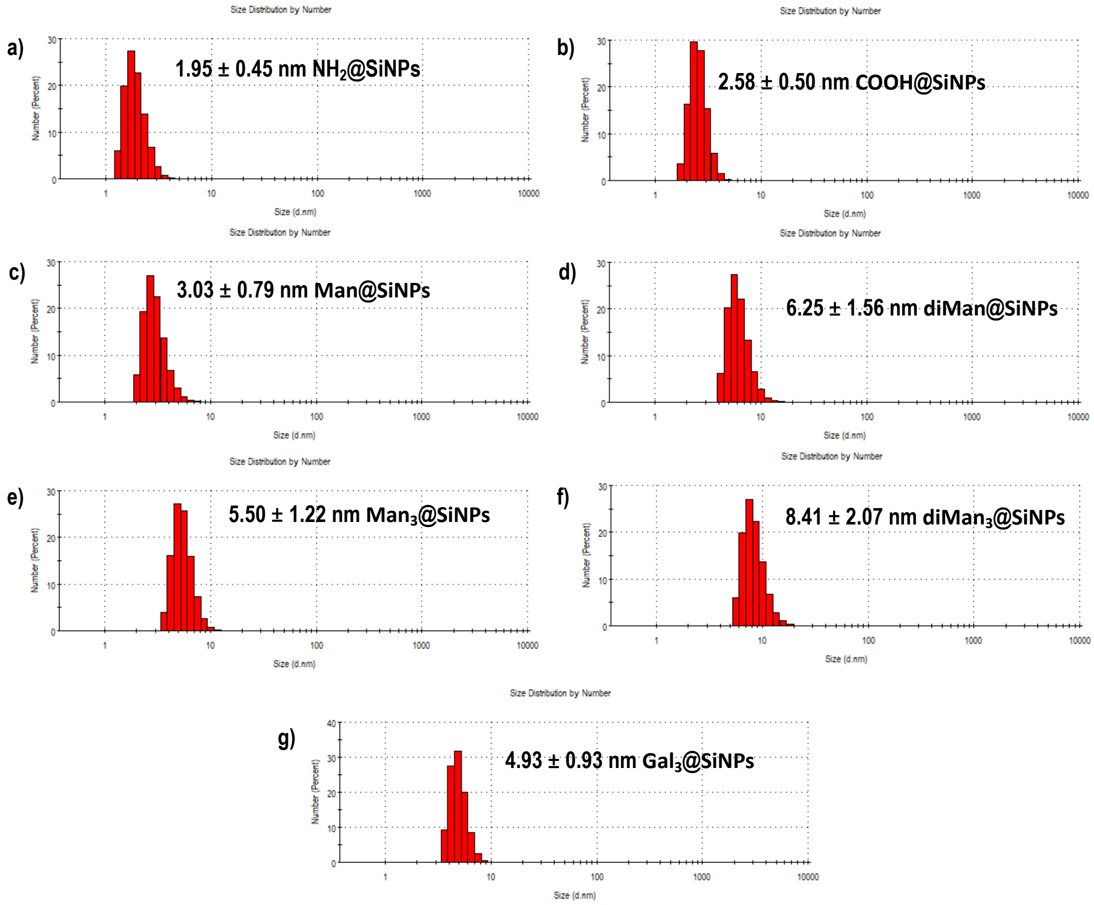

Figure S26. DLS number size histograms of a) NH_2@SiNPs, b) COOH@SiNPs, c) Man@SiNPs **1**, d) diMan@SiNPs **2**, e) Man_3SiNPs **3**, f) diMan_3SiNPs **4** and g) Gal_3SiNPs **5**.

6. TGA analysis

TGA analyses were carried out under nitrogen in a TA/TGA-Q500 apparatus. The sample (~0,5 mg) was introduced inside a platinum crucible and equilibrated at 90 ºC followed by a 10 ºC min^{-1} ramp between 90 and 1000 ºC.

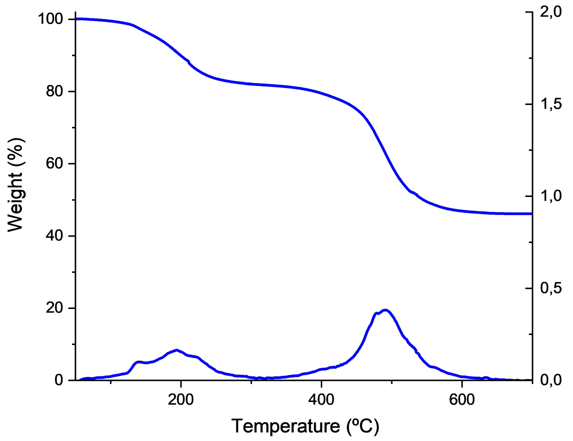

Figure S27. TGA of COOH@SiNPs with a weight loss of 16,8 % at 293 ºC, related with the carboxylic acid functionalities.

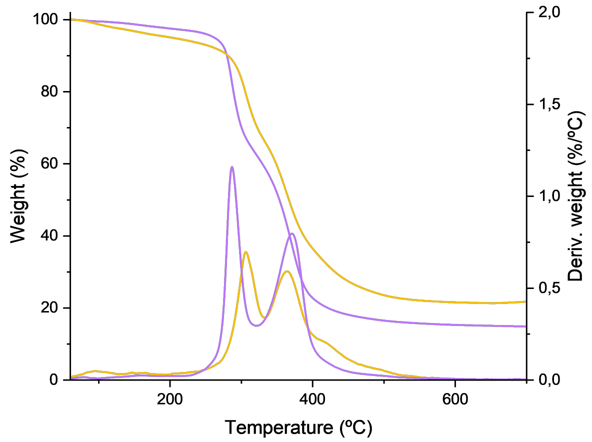

Figure S28. TGA comparative of Man_3@SiNPs mannose **3** (yellow) with Gal_3@SiNPs **5** (purple).

7. Infrared

NH_2@SiNP, COOH@SiNP, Man@SiNPs **1**, Man_3@SiNPs **3** and Gal_3@SiNPs **5** were analysed through a KBr pill using JASCO FT/IR-6200 IRT-5000 instrument. diMan@SiNPs **2** and $diMan_3$SiNPs **4** were directly analysed using a platinum ATR Bruker tensor 27 instrument.

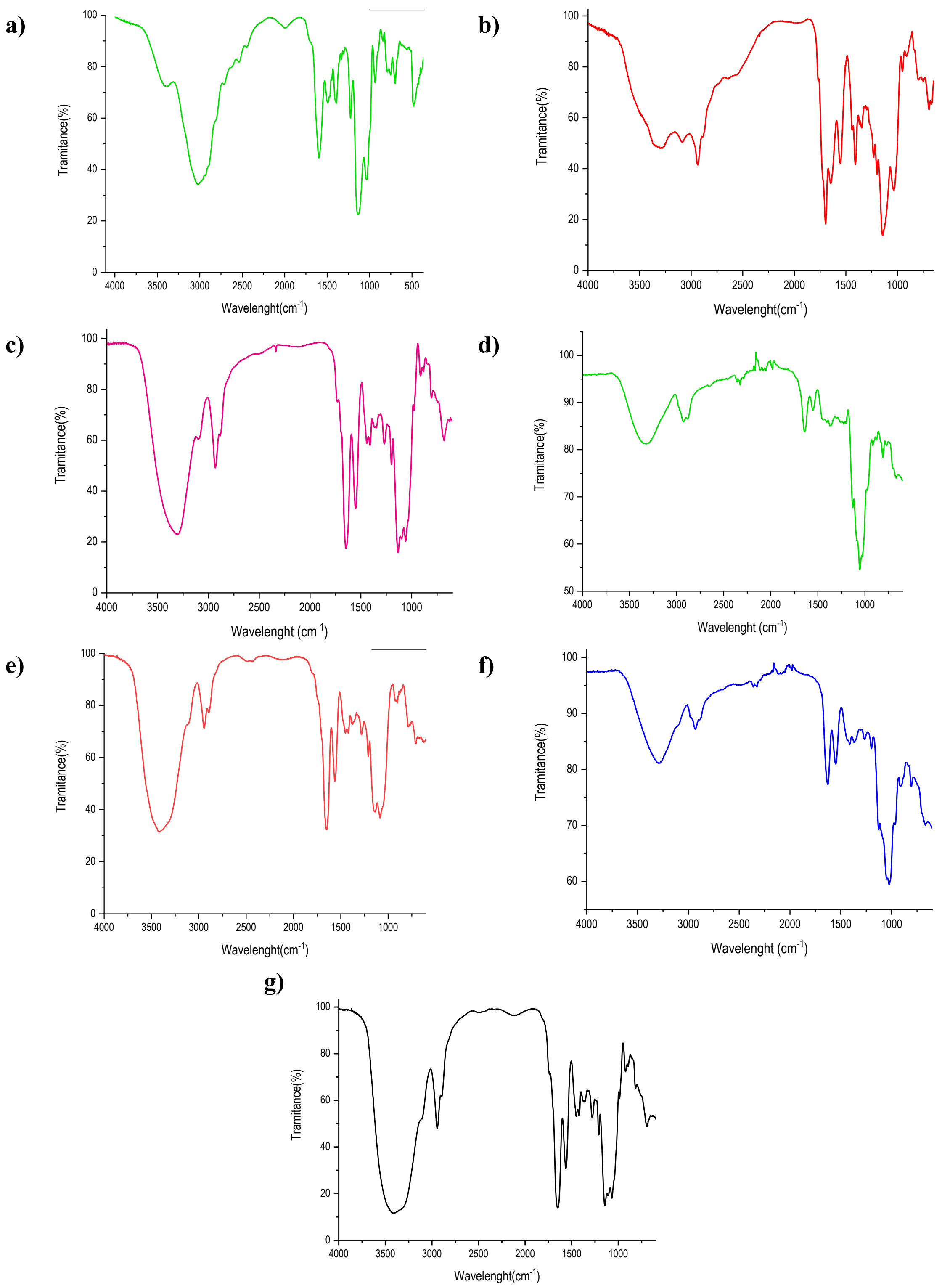

Figure S29. Infrared spectra of a) NH_2@SiNPs b) COOH@SiNPs, c) Man@SiNPs **1**, d) diMan@SiNPs **2**, e) Man_3@SiNPs **3**, f) $diMan_3$@SiNPs **4** and g) Gal_3@SiNPs **5**.

8. XPS analysis

XPS analysis was carried out using a SPECS GmbH (PHOIBOS 150 9MCD) spectrometer operating in the constant analyzer energy mode. A non-monochromatic aluminium X-ray source (1486.61 eV) was used with a power of 200 W and voltage of 12 kV. Pass energies of 75 and 25 eV were used for acquiring both survey and high-resolution spectra, respectively. Survey data were acquired from kinetic energies of 1487-400 eV with an energy step of 1 eV and 100 ms dwell time per point. The high-resolution scans were taken around the emission lines of interest with 0.1 eV steps and 100 ms dwell time per point. SpecsLab Version 2.48 software was used for spectrometer control and data handling. The semi-quantitative analysis was performed from the C 1s (284.6 eV) signal. The samples were introduced as pellets of 8 mm diameter.

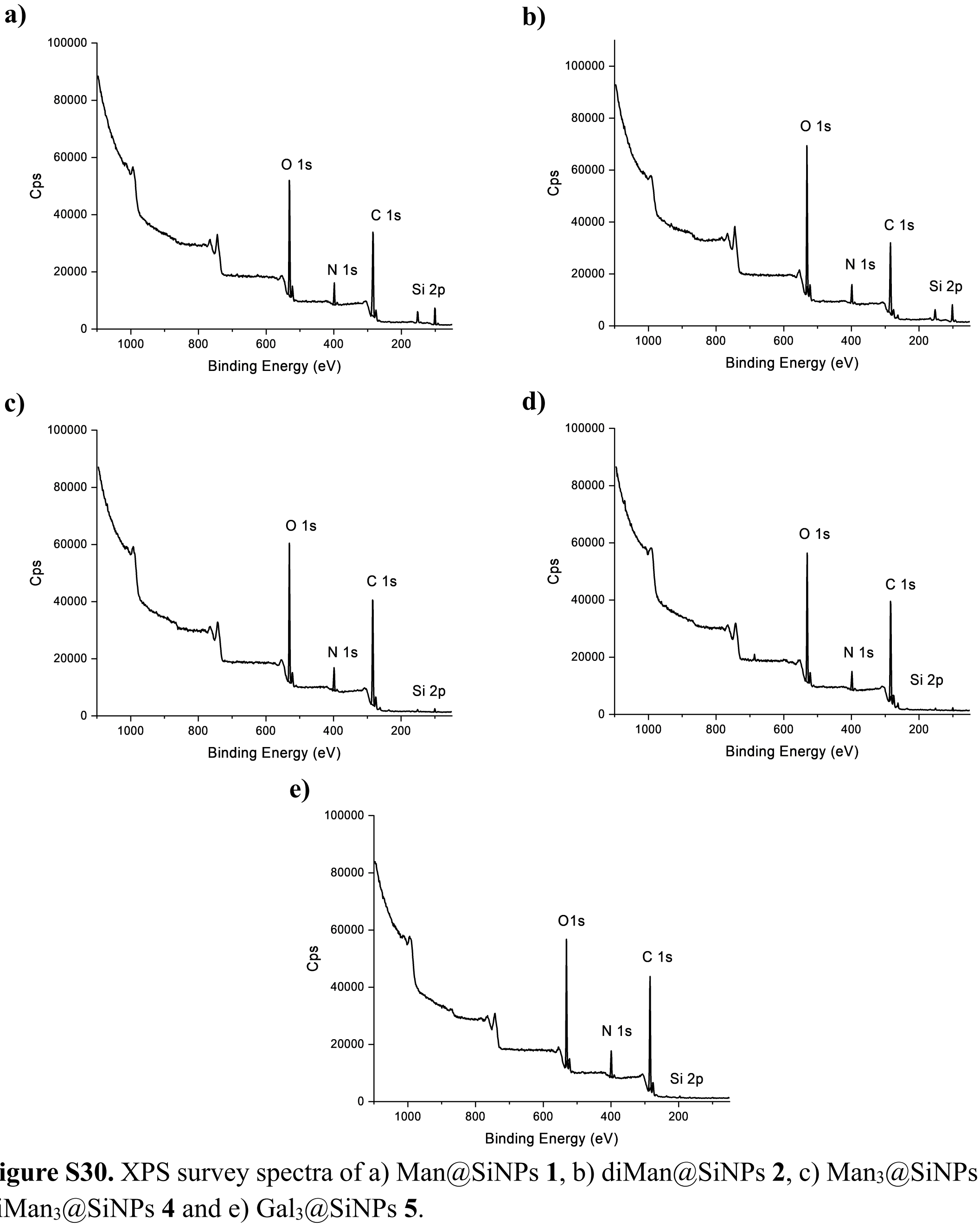

Figure S30. XPS survey spectra of a) Man@SiNPs **1**, b) diMan@SiNPs **2**, c) Man_3@SiNPs **3**, d) $diMan_3$@SiNPs **4** and e) Gal_3@SiNPs **5**.

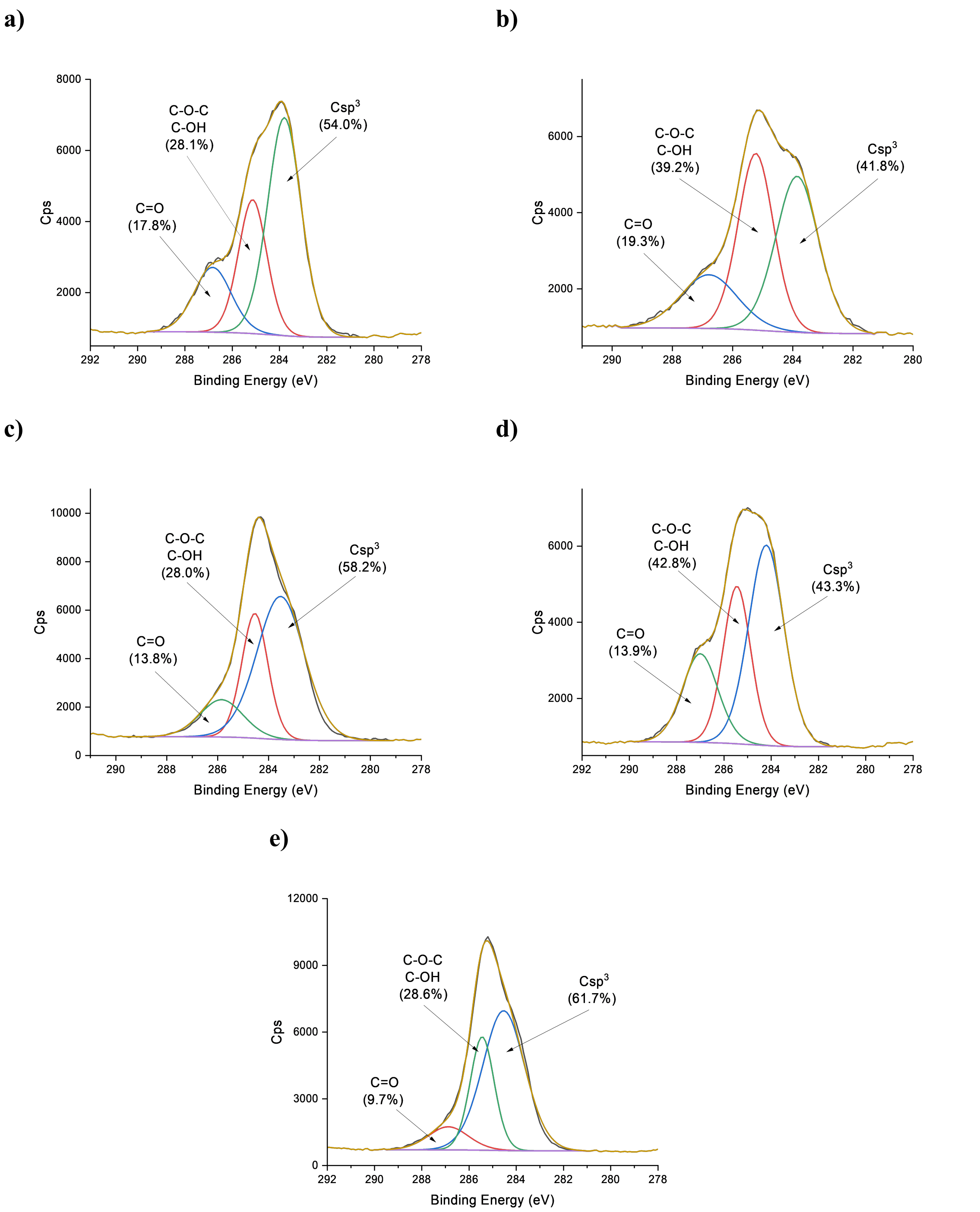

Figure S31. XPS high resolution spectra of a) Man@SiNPs **1**, b) diMan@SiNPs **2**, c) Man_3@SiNPs **3**, d) $diMan_3$@SiNPs **4** and e) Gal_3@SiNPs **5**.

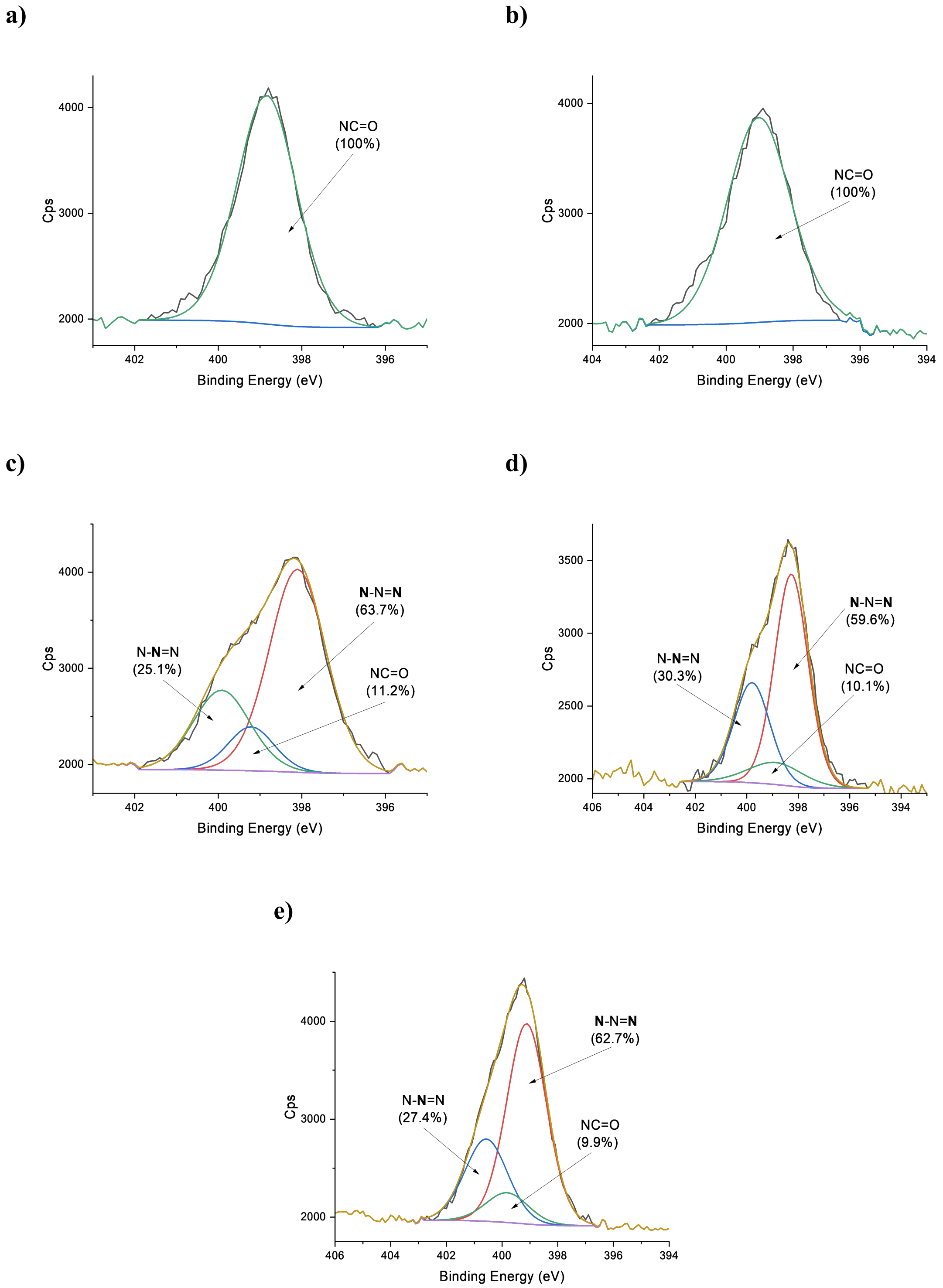

Figure S32. XPS high resolution spectra of N1s of a) Man@SiNPs **1**, b) diMan@SiNPs **2**, c) Man_3@SiNPs **3**, d) diMan_3@SiNPs **4** and e) Gal_3@SiNPs **5**.

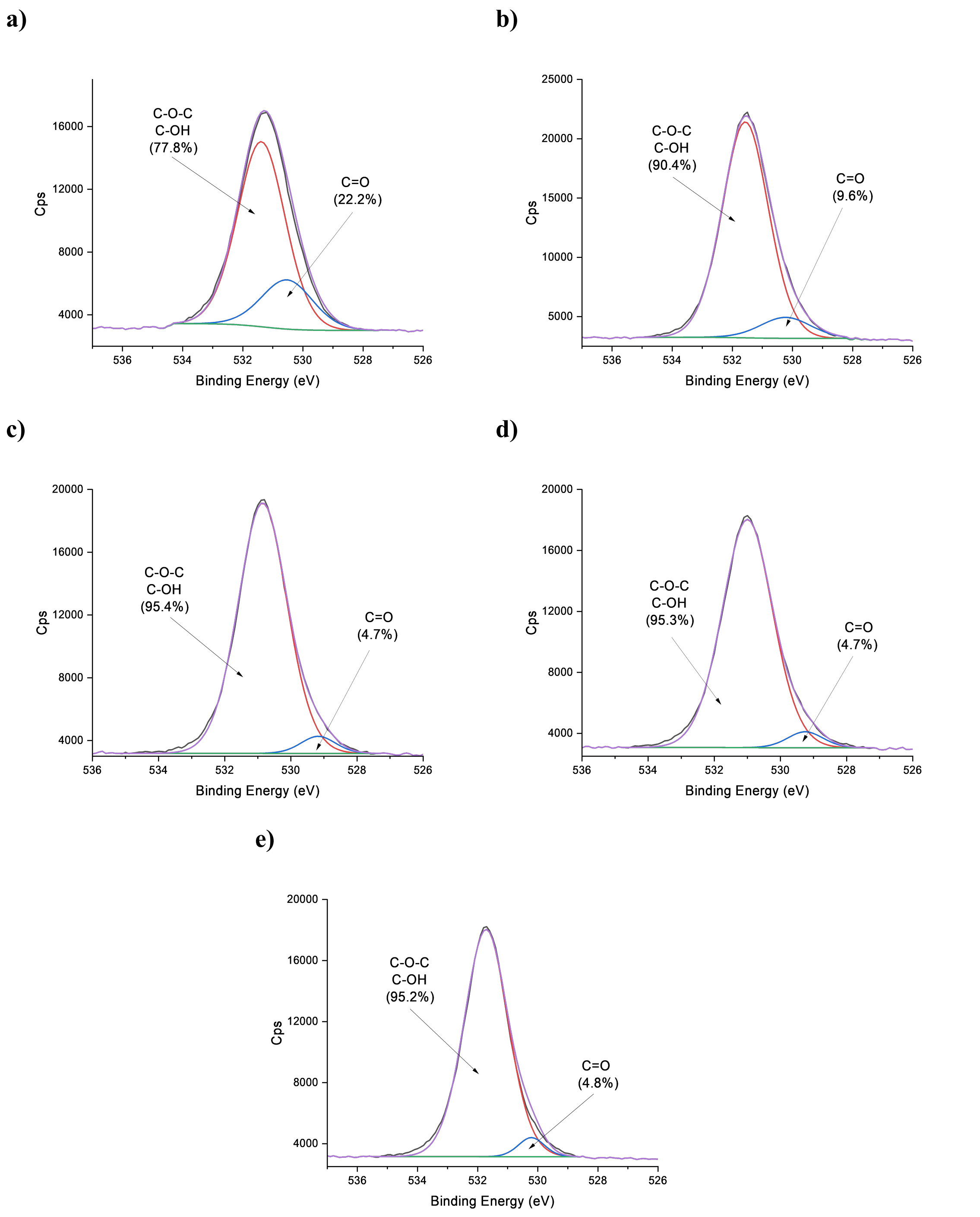

Figure S33. XPS high resolution spectra of O1s of a) Man@SiNPs **1**, b) diMan@SiNPs **2**, c) Man_3@SiNPs **3**, d) $diMan_3$@SiNPs **4** and e) Gal_3@SiNPs **5**.

9. Determination of the amount of carbohydrates in the glycoSiNPs by UV-Visible method. The amount of carbohydrates in the glycoSiNPs was estimated following a procedure described elsewhere.[1] In brief, the amount of carbohydrate could be determined by UV–Vis spectroscopy (Jasco V-650) in H_2SO_4/H_2O (λ = 315 nm) at 25°C after performing a calibration curve for D-mannose and dendron of 3 mannoses **14**. The values obtained correspond to 3 independent experiments with error bars corresponding to the standard errors of the mean

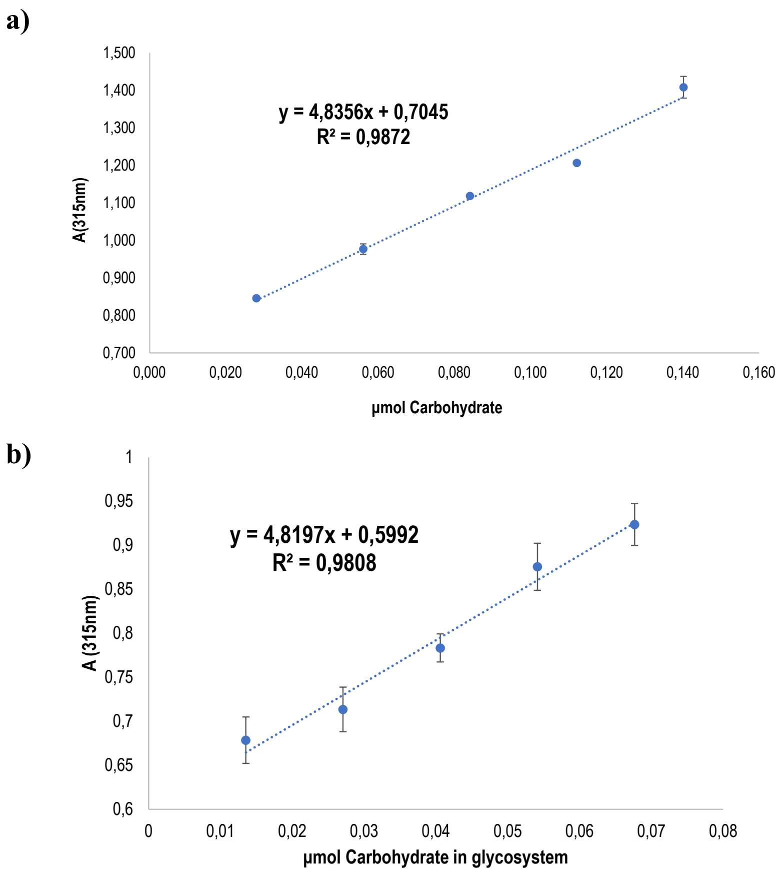

Figure S34. Calibration curve for a) D-mannose and b) trivalent glycodendron of mannose **14.**

10. Lectin (ConA) binding assays by DLS.

ConA agglutination assays were used to investigate the ability of the glycoSiNPs to bind mannose-binding lectins by DLS. DLS measurements were performed on a Malvern Nano ZS (Malvern Instruments, U.K.) operating at 633 nm with a 173° scattering angle. Measurements were made in a semi-micro 10 mm path-lenght PMMA cuvettes maintained at 298K. ConA was dissolved at 0.45 mg/mL in 0.1 M Tris-HCl pH 7.2 containing 0.9 M KCl, 1 mM $MnCl_2$ and 1 mM $CaCl_2$. 350 µL of ConA were placed into a DLS semi-micro cuvette, and DLS measurements for the lectin alone were performed. Afterwards, increasing amounts (6 µL each, see Table S2) of a solution of glycoSiNPs were added and DLS measurements were recorded 5 min after mixing. Three replicates of each sample were measured.

Table S2. Stock solution of glycoSiNPs 1-5 and nmol of carbohydrate added per aliquot (6 µL) in DLS lectin binding assay.

glycoSiNP	glycoSiNPs stock solution (mg/mL x 10^{-2})	nmol of carbohydrate* in each addition (6 µL)
**Man@SiNPs (1)**	1.53	0.1
**diMan@SiNPs (2)**	1.4	0.05
**Man_3@SiNPs (3)**	1.2	0.033
**diMan_3@SiNPs (4)**	1.1	0.015
**Gal_3@SiNPs (5)**	1.2	0.033

*nmol of carbohydrate in the corresponding glycoSiNPs

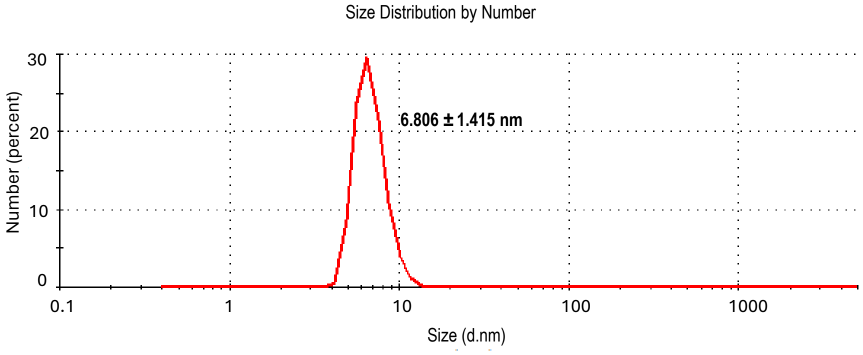

Figure S35. DLS number size histogram of ConA lectin.

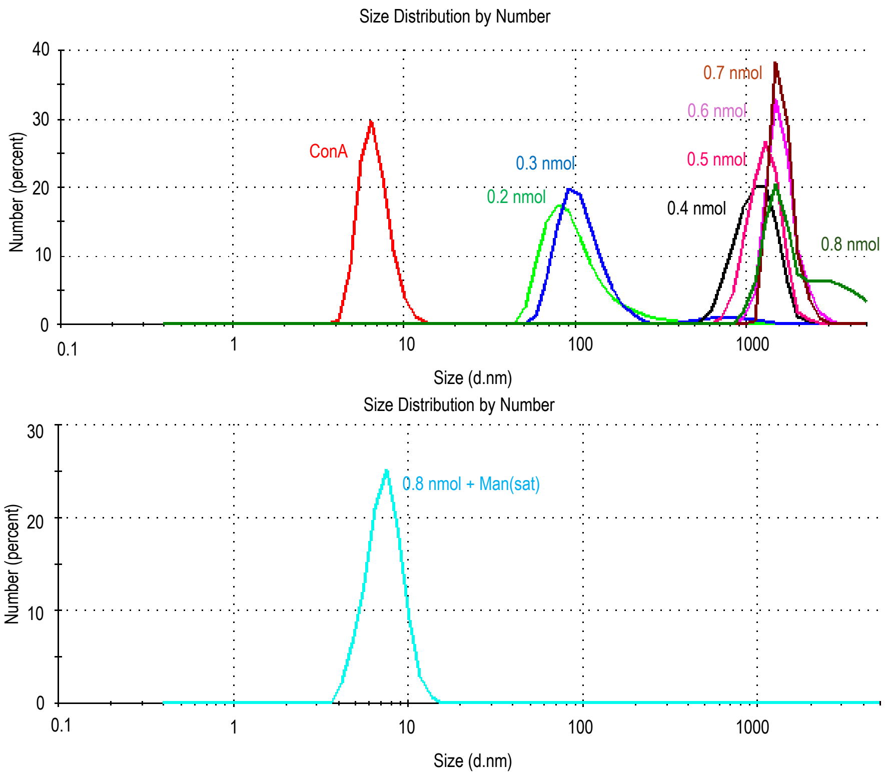

Figure S36. DLS number size histograms of titration of ConA with Man@SiNPs **1** (top) and after addition of a saturated solution of α-D-mannose (down).

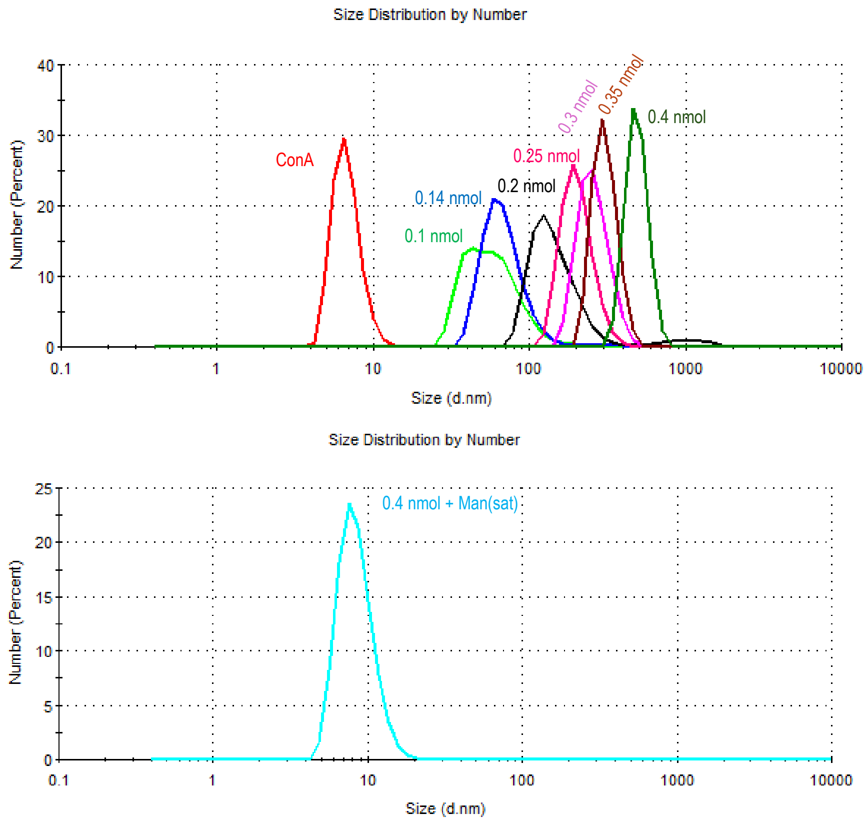

Figure S37. DLS number size histograms of titration of ConA with diMan@SiNPs **2** (top) and after addition of a saturated solution of α-D-mannose (down).

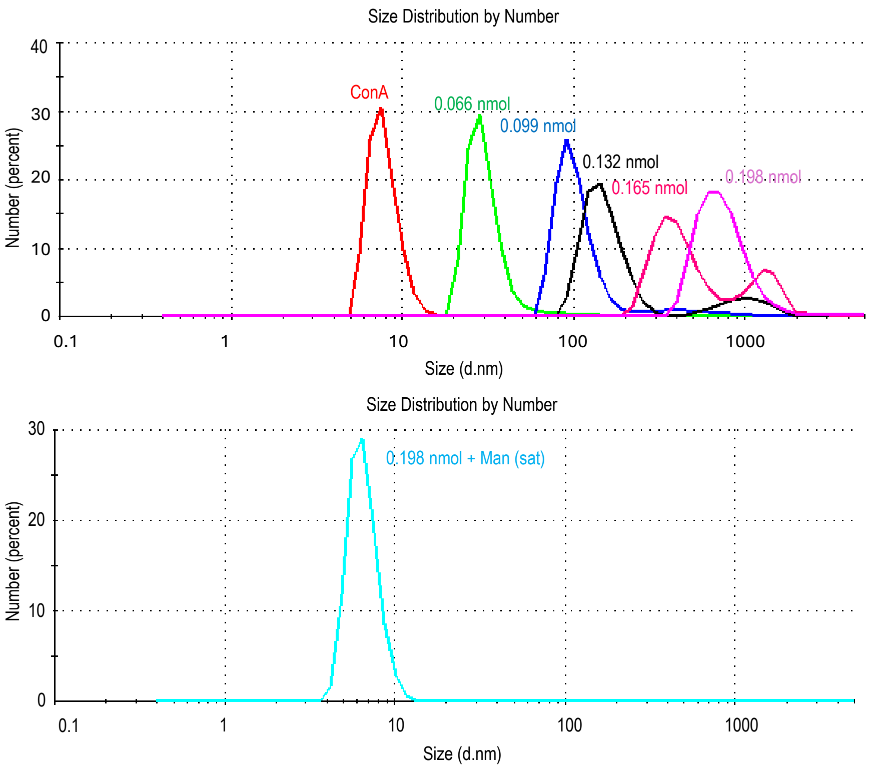

Figure S38. DLS number size histograms of titration of ConA with Man$_3$@SiNPs **3** (top) and after addition of a saturated solution of α-D-mannose (down).

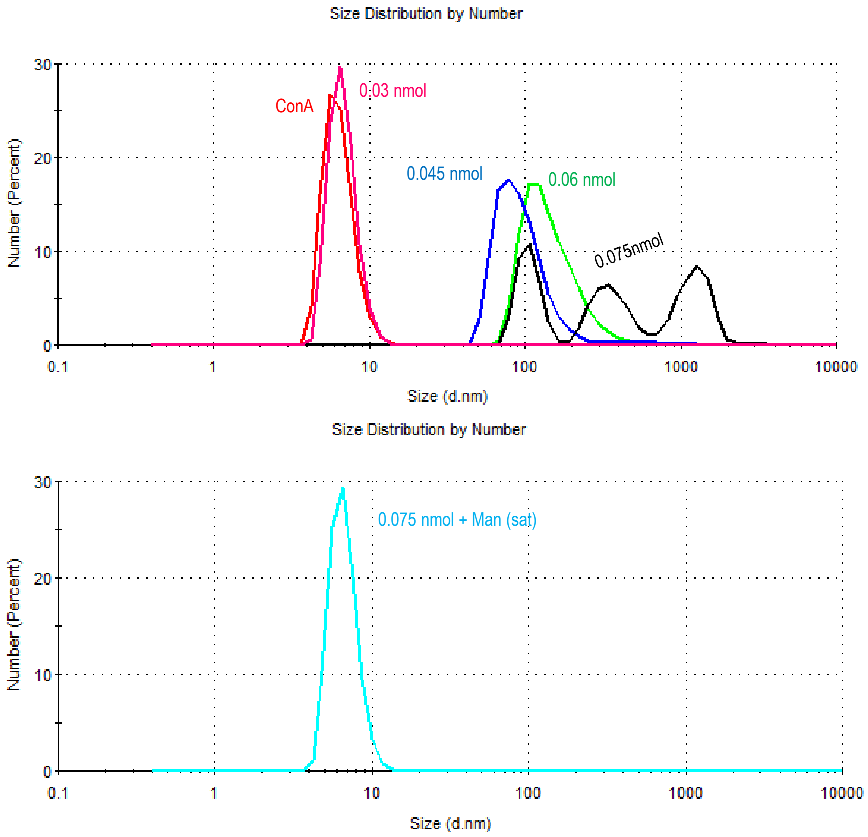

Figure S39. DLS number size histograms of titration of ConA with diMan$_3$@SiNPs **4** (top) and after addition of a saturated solution of α-D-mannose (down).

11. Lectin (ConA) binding assays by turbidimetry assays.

UV-Vis measurements were performed on a Jasco V-650 spectrometer using 1cm path-length quarzt cuvettes. A solution of each glycoSiNPs (142.8 µg/mL in 0.1 M Tris-HCl, 0.9 M KCl, 1 mM $MnCl_2$, 1 mM $CaCl_2$, pH = 7.2) was sequentially added, in 15 µL portions (see Table S3), to a solution of ConA (350 µL, 0.45 mg/mL in the same buffer). After each addition, the resulting mixture was allowed to stand for 5 min before the absorbance was measured at 276 nm and the following glycoSiNPs addition was performed. To determine the most suitable wavelength to measure the ConA-NGs aggregates (276 nm), a complete absorption spectrum was first acquired. The values obtained correspond to 3 independent experiments with error bars corresponding to the standard errors of the mean (Table S4).

Table S3. nmol of carbohydrate added per aliquot (15 µL) in UV-Vis turbidimetry assay.

glycoSiNP	glycoSiNPs stock solution (mg/mL)	nmol of carbohydrate* in each addition (15 µL)
**Man@SiNPs (1)**	0.153	2.6
**diMan@SiNPs (2)**	0.14	1.3
**Man_3@SiNPs (3)**	0.12	0.86
**diMan_3@SiNPs (4)**	0.11	0.45
**Gal_3@SiNPs (5)**	0.12	0.86

*nmol of carbohydrate in the corresponding glycoSiNPs

Table S4. Absorbance values of complex ConA/glycoSiNPs at 276 nm after each addition

Man@SiNPs (1)

nmol Man	Absorbance
0.00	0.00 ± 0.01
2.6	0.07 ± 0.01
5.2	0.11 ± 0.01
7.8	0.15 ± 0.01
10.4	0.20 ± 0.01
13.00	0.25 ± 0.04
15.6	0.30 ± 0.06
18.2	0.28 ± 0.04
20.8	0.26 ± 0.01
23.4	0.23 ± 0.02
26.00	0.19 ± 0.04
28.6	0.16 ± 0.05
31.2	0.13 ± 0.06
33.8	0.10 ± 0.02
36.4	0.10 ± 0.02

diMan@SiNPs (2)

nmol diMan	Absorbance
0.00	0.00 ± 0.00
1.30	0.06 ± 0.01
2.60	0.11 ± 0.02
3.90	0.17 ± 0.02
5.20	0.22 ± 0.02
6.50	0.26 ± 0.02
7.80	0.23 ± 0.02
9.10	0.22 ± 0.02
10.40	0.16 ± 0.00
11.70	0.13 ± 0.01
13.00	0.08 ± 0.02
14.30	0.05 ± 0.01

Man$_3$@SiNPs (3)

nmol Man	Absorbance
0.00	0.03 ± 0.05
0.83	0.15 ± 0.07
1.66	0.25 ± 0.11
2.49	0.34 ± 0.16
3.32	0.42 ± 0.18
4.15	0.38 ± 0.10
4.98	0.33 ± 0.05
5.81	0.26 ± 0.02
6.64	0.18 ± 0.01
7.47	0.11 ± 0.03
8.30	0.09 ± 0.01
9.13	0.08 ± 0.02

diMan$_3$@SiNPs (4)

nmol diMan	Absorbance
0.00	0.00 ± 0.00
0.47	0.07 ± 0.02
0.94	0.15 ± 0.04
1.41	0.20 ± 0.08
1.88	0.30 ± 0.08
2.35	0.39 ± 0.10
2.82	0.48 ± 0.14
3.29	0.48 ± 0.06
3.76	0.41 ± 0.03
4.23	0.32 ± 0.02
4.70	0.26 ± 0.02
5.17	0.20 ± 0.02
5.64	0.14 ± 0.04
6.11	0.08 ± 0.05
6.58	0.07 ± 0.02

Gal$_3$@SiNPs (5)

nmol Gal	Absorbance
0.00	0.00 ± 0.00
0.83	0.03 ± 0.01
1.66	0.06 ± 0.02
2.49	0.07 ± 0.05
3.32	0.10 ± 0.07
4.15	0.10 ± 0.06
4.98	0.10 ± 0.06
5.81	0.10 ± 0.06
6.64	0.10 ± 0.06
7.47	0.10 ± 0.06
8.30	0.10 ± 0.07

Note: nmol of carbohydrate Man, diMan and Gal is referred to the content in the corresponding glycoSiNPs.

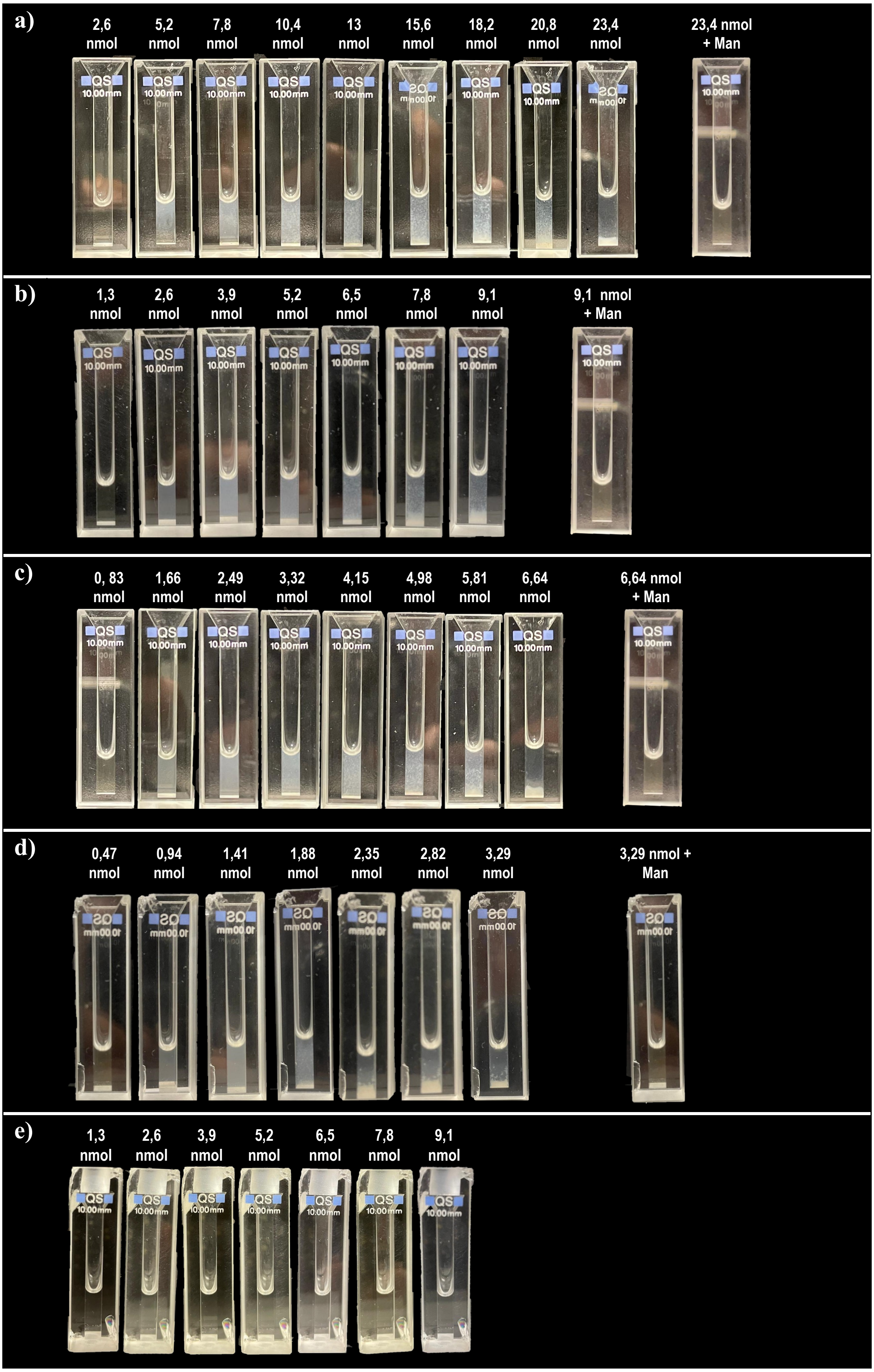

Figure S40. Images of titration of ConA with a) Man@SiNPs **1**, b) diMan@SiNPs **2**, c) Man_3@SiNPs **3**, d) diMan_3@SiNPs **4** and e) Gal_3@SiNPs **5**.

12. Biological assays

Culture cells. Human embryonic kidney cells (293T/17; ATCC-CRL-11268), were cultured in Dulbecco´s modified Eagle medium (DMEM) supplemented with 10% heat-inactivated fetal bovine serum (FBS), 25 μg/mL gentamycin and 2 mM L-glutamine. Jurkat and Jurkat stably expressing DC-SIGN or L-SIGN lectin were cultured in Roswell Park Memorial Institute (RPMI) supplemented with 10% heat-inactivated fetal bovine serum (FBS), 25 μg/mL gentamycin and 2 mM L-glutamine. Cells were maintained at 37ºC in an environment with 5% CO_2.

Production of recombinant viruses. Recombinant lentiviruses were produced according to a transient-transfection protocol using 293T cells. The viral construction was pseudotyped with Ebolavirus (EBOV) envelope glycoprotein (GP) or vesicular stomatitis virus envelope GP (VSV-G) that expressed luciferase as a reporter of the infection. One day before transfection a total of 6x106 293T cells (producer cells) were seeded onto 10 cm plates. Few minutes before transfection, medium was changed to 9 mL DMEM and chloroquine was added to 25μM final concentration. Transfection contains 2M $CaCl_2$, H_2O, 2xHBS (Hepes Buffer Saline) pH 7.00 and 2 μg of EBOV-GP or 6 μg of VSV-G and 18 μg of pNL4-3 luc. HBS/DNA solution was bubbled and then gently dropped onto medium. After 8 hours of incubation at 37° C with 5% CO_2, medium on transfection plates was changed to 10 mL DMEM and once again one day after transfection to 7 mL DMEM. Transfection supernatants were harvested after 48 h, centrifuged at 1200 rpm for 10 minutes at RT to remove cell debris, and stored frozen at -80° C. Infectious titers were estimated by serial dilutions on 293T cells and infectivity of the pseudotype virus was assessed by luciferase activity with the Steady-Glo luciferase assay system (Promega Corporation, Madison, WI).

Infection in cis. Infection was performed on Jurkat cells (CD4+ T-lymphocyte cell line) expressing the receptor DC-SIGN or L-SIGN on its surface. Since Ebola virus does not infect T-lymphocytes, its entry is absolutely dependent on this receptor for infection of Jurkat cells. Jurkat DC/L-SIGN$^+$ cells (1.5 x 10^5) were plated into each well of 96-well plate, incubated at RT for 20 minutes with the carbohydrate-based compounds and then challenged with 5000 Tissue Culture Infective Doses (TCID) of recombinant viruses. After 48 h of incubation cells were washed twice with PBS and lysed for luciferase assay. Initially, a concentration of 100 μg/mL was used and only for those compounds that showed a clear inhibitory activity, lower concentrations were further used (from 1 ng/mL to 100 μg/mL). As a virus control, infection with VSV-G pseudoviruses was performed in the same conditions. Infection with VSV-G is independent of the presence of the DC-SIGN receptor. As a compound control, Gal_3@SiNPs **5** with galactose instead of mannose were included in the experiments.

Statistical analysis. The values of percentage of inhibition of the infection presented on the graph correspond to the mean of 3 independent experiments with error bars corresponding to the standard errors of the mean. The 50% Inhibitory Concentration (IC_{50}) values were estimated using GraphPad Prism v4.0 with a 95% confidence interval and settings for normalized dose-response curves.

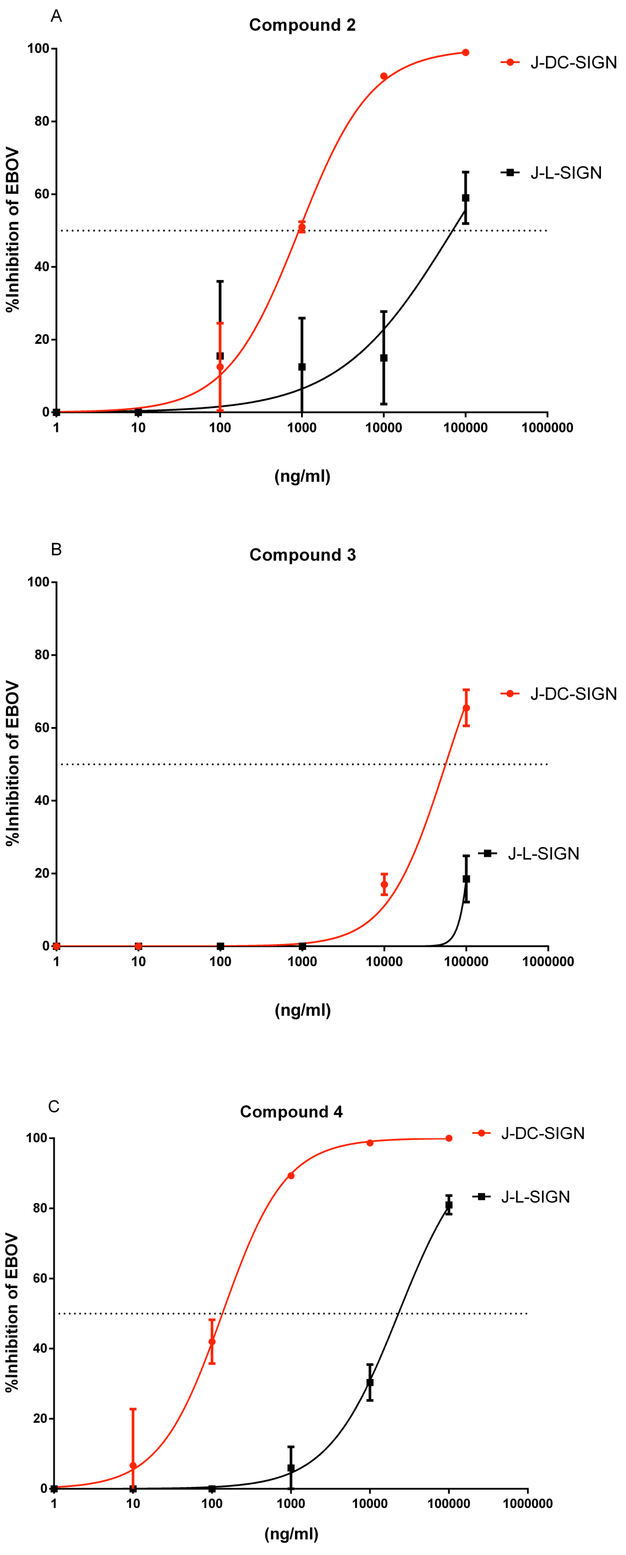

Figure S41. Effects of a) diMan@SiNPs **2**, b) Man_3@SiNPs **3** and c) diMan_3@SiNPs **4** on Ebola-GP pseudotyped virus infection on Jurkat-DC-SIGN (red) or Jurkat L-SIGN (black).

13. Reference

1. Albalasmeh, A. A.; Berhe, A. A.; Ghezzehei, T. A., A new method for rapid determination of carbohydrate and total carbon concentrations using UV spectrophotometry. *Carbohydr Polym* **2013,** *97* (2), 253-61.
